# Baleen boom and bust: a synthesis of mysticete phylogeny, diversity and disparity

**DOI:** 10.1098/rsos.140434

**Published:** 2015-04-15

**Authors:** Felix G. Marx, R. Ewan Fordyce

**Affiliations:** 1 Department of Geology, University of Otago, Dunedin 9054, New Zealand; 2 Department of Geology and Palaeontology, National Museum of Nature and Science, Tsukuba 305-0005, Japan

**Keywords:** Mysticeti, baleen whales, phylogenetics, diversity, disparity, evolutionary rates

## Abstract

A new, fully dated total-evidence phylogeny of baleen whales (Mysticeti) shows that evolutionary phases correlate strongly with Caenozoic modernization of the oceans and climates, implying a major role for bottom-up physical drivers. The phylogeny of 90 modern and dated fossil species suggests three major phases in baleen whale history: an early adaptive radiation (36–30 Ma), a shift towards bulk filter-feeding (30–23 Ma) and a climate-driven diversity loss around 3 Ma. Evolutionary rates and disparity were high following the origin of mysticetes around 38 Ma, coincident with global cooling, abrupt Southern Ocean eutrophication and the development of the Antarctic Circumpolar Current (ACC). Subsequently, evolutionary rates and disparity fell, becoming nearly constant after approximately 23 Ma as the ACC reached its full strength. By contrast, species diversity rose until 15 Ma and then remained stable, before dropping sharply with the onset of Northern Hemisphere glaciation. This decline coincided with the final establishment of modern mysticete gigantism and may be linked to glacially driven variability in the distribution of shallow habitats or an increased need for long-distance migration related to iron-mediated changes in glacial marine productivity.

## 2. Introduction

Baleen whales are the largest animals on the Earth and a major component of the ocean ecosystem as mass predators and large-scale nutrient distributors [1–3]. Mysticete evolution is likely to have been profoundly affected by palaeoenvironmental change [4–6], but our understanding of evolutionary drivers has been hampered by a lack of comprehensive, stable phylogenies, meagre data on macroevolutionary dynamics and a sparse early fossil record. Past studies have fundamentally disagreed on the phylogenetic position, and even monophyly, of many extant and extinct taxa [7–11], with major implications for tree topology and molecular estimates of divergence times. Such problems probably reflect limited taxonomic sampling, which is known to compromise both phylogenetic accuracy [12] and macroevolutionary inferences [13]. Another key problem is the presentation of morphological data as simple character-based scorings, which are usually unsupported by illustrations and hence difficult to comprehend or repeat [14].

**Figure 1.**
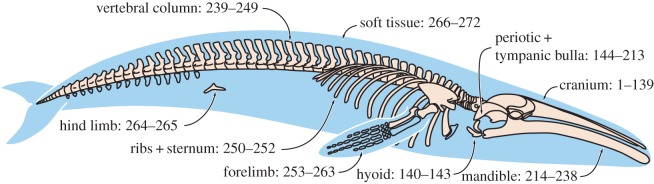
Distribution of morphological characters. Numbers refer to morphological character numbers as stored on MorphoBank (www.morphobank.org), project 687.

We address these issues through a comprehensive investigation of mysticete macroevolutionary dynamics based on the most detailed, illustrated, data matrix of extinct and extant baleen whales. We score 90 taxa—nearly all for which satisfactory material has been described—for 37 646 molecular and 272 morphological characters, covering all parts of the skeleton (figure 1). To promote repeatability and to clarify recognition (homology) of characters, morphological scorings are illustrated by over 4000 annotated specimen images stored on MorphoBank (www.morphobank.org; project 687) [15]. We analyse our data using a recently developed total-evidence dating technique [16] to derive (i) a fully dated phylogeny, and (ii) both per-branch and average (lineage-through-time) estimates of combined phenotypic and genomic rates of evolution. Unlike more traditional molecular clock dating, total-evidence dating explicitly incorporates fossil taxa and can simultaneously infer phylogeny, evolutionary rates and divergence dates for all nodes (both living and extinct) based on a combined molecular/morphological clock. The inclusion of dated extinct taxa largely obviates the need for node-based calibrations, whose phylogenetic position needs to be determined *a priori* [16,17]. Total-evidence dating allows the origin of a particular clade to pre-date its oldest member even if one or both of its most basal lineages (or taxa) are entirely extinct: the more apomorphic the latter (relative to the last common ancestor), the older the inferred age of the clade [18].

To complement our estimate of evolutionary rates, we use the fully dated tree topology and the underlying morphological data to derive phylogenetically informed estimates of mysticete taxonomic and morphological diversity (disparity) over the past 37 Ma. These three measures chart diverse aspects of mysticete evolution during mid-Late Caenozoic from a variety of angles. Finally, we synthesize all of these metrics to derive the first comprehensive overview of mysticete macroevolution and discuss the latter in light of large-scale palaeoenvironmental and biotic change.

## 3. Material and methods

### 3.1 Data compilation

We compiled a data matrix including 90 taxa, scored for 272 morphological and 37 646 molecular characters. Of the 90 taxa, 86 represent mysticetes, 76 of which have been formally described. This constitutes roughly 41% of the described 185 mysticete species listed in the Paleobiology Database [19]. These figures warrant cautious interpretation, because many of the taxa that we did not include are only known from fragmentary and often non-diagnostic material (e.g. various species of *Mesocetus*). A review of all described species is beyond the scope of this study, but see Steeman [20] for a first attempt to deal with a part of this issue. We consider that as much as 20–30% of described species might be *nomina dubia*.

All of our morphological scorings are based on primary observation, and illustrated with one or more of 4228 specimen photographs on MorphoBank (www.morphobank.org; project 687). Twenty-five characters show a morphological transition series in three or more states and were hence ordered (electronic supplementary material). Overlaps with other recent studies are indicated in the online character descriptions (in the ‘Comments’ field). For 18 taxa (*Balaenoptera omurai*; ‘*Balaenoptera*’ *ryani*; *Cephalotropis coronatus*; ‘*Cetotherium*’ *megalophysum*; ChM PV4745; *Llanocetus denticrenatus*; *Morenocetus parvus*; NMNZ MM001630; OCPC 1178; OU 22026, 22044, 22224, 22545, 22705, GS10897; *Peripolocetus vexillifer*; UWBM 84024; ZMT 67), the photographs used to confirm character states are not published online because the particular specimen(s) are under study by other researchers. These images will be made available once the material is formally documented. Molecular data were taken from McGowen *et al.* [21] and pruned to match our taxon sample, following which 37 013 DNA nucleotide and 633 binary characters (552 gap characters and 81 transposons) remained. Even among the remaining binary characters many were invariant, which led to the automatic exclusion of a further 430 gap characters and 16 transposons during the phylogenetic analysis.

### 3.2 Non-clock phylogenetic analyses

We retained the original alignment of the molecular partition [21], but divided the nucleotide data by both gene and codon position. We then used the ‘greedy’ search scheme of PartionFinder v. 1.1.1 [22] to determine the final partitioning scheme and fit a series of models (GTR, GTR+G, GTR+I+G, HKY, HKY+G, HKY+I+G) previously suggested for cetacean nucleotide data [21,23,24], based on the Bayesian Information Criterion. The results suggested a preferred partitioning scheme of 17 sets (plus three sets for the gap characters, transposons and the morphological data, respectively), variously fitting all of the tested models, except GTR (for details see Nexus file in electronic supplementary material). A Bayes Factor (BF) comparison [25], calculated using the stepping stone algorithm implemented in MrBayes v. 3.2.2 [16,26,27], strongly favoured variable rates of change among morphological characters over equal rates of evolution (BF∼277).

Based on these preliminary results, we ran two separate analyses of: (i) the morphological partition only, using the Markov maximum-likelihood model and a gamma parameter to allow for variable rates across traits (Mk+*Γ*); and (ii) the combined morphological/molecular supermatrix, partitioned as described above. For the nucleotide data, we used the substitution models suggested by PartitionFinder, whereas the gap and transposon characters were assigned a binary model, and the morphological data the Mk+*Γ* model, as above. For both analyses, the coding bias for the morphological data was set to ‘informative’ to correct for the absence of constant and autapomorphic characters. Markov Chain Monte Carlo (MCMC) analyses were run in MrBayes v. 3.2.2 for 20 and 50 million generations, respectively, on the Cyberinfrastructure for Phylogenetic Research Science Gateway [28]. Both analyses used three replicate runs (comprising one cold and three heated chains) sampled every 1000 generations, with the first 25% discarded as burn-in. To facilitate chain mixing, the temperature setting was reduced to 0.1. Convergence was judged based on the average standard deviation of split frequencies (all less than 0.01) and AWTY [29]. Finally, the results were summarized in a majority-rule tree drawing on all three independent runs.

### 3.3 Total-evidence dating

**Figure 2.**
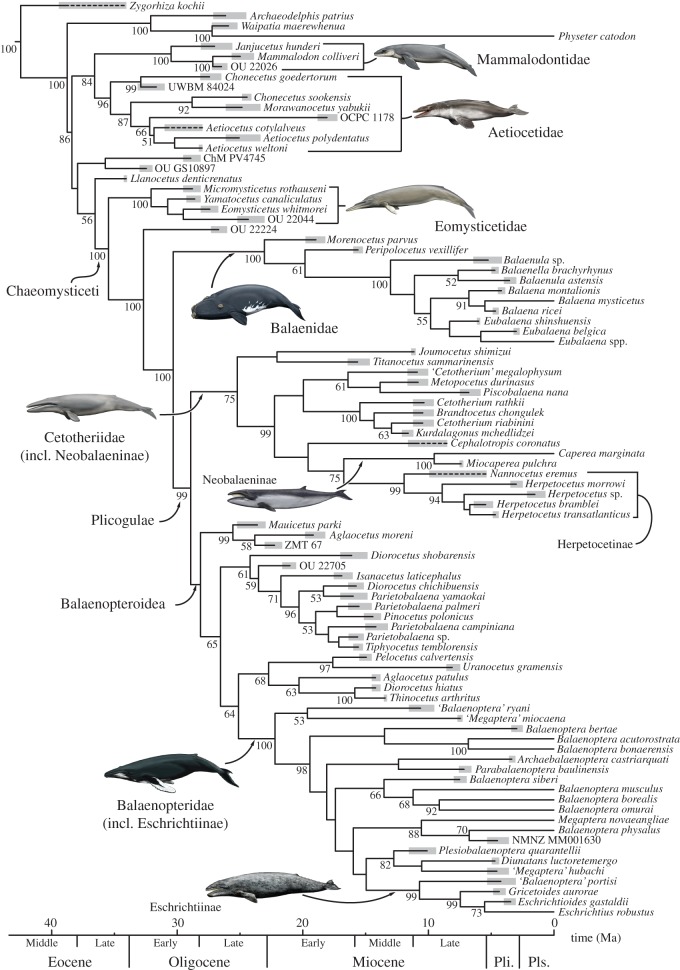
Dated total-evidence tree of mysticete phylogenetic relationships. Majority-rule consensus tree (showing all compatible clades; ‘allcompat’ option in MrBayes) resulting from the combined analysis of the entire dataset (272 morphological, 37 646 molecular characters). Numbers next to nodes represent posterior probabilities (only values more than or equal to 50 are shown). Stippled lines represent fossil material postdating the oldest occurrence of a given species. Thick grey bars represent actual stratigraphic ranges (Material and methods, and electronic supplementary material). See electronic supplementary material, figure S1, for the range of uncertainty (95% HPD) associated with each node. Pli., Pliocene; Pls., Pleistocene.

Our dating analyses follow the methodology of Ronquist *et al*. [16], as implemented in MrBayes. To prepare for the total-evidence dating analysis [16], we reviewed the stratigraphic ranges of all included fossil taxa (electronic supplementary material). We then used this information to calibrate all fossil tips, using uniform distributions to account for uncertainty in their stratigraphic placement [30]. Where extinct taxa were represented by specimens of markedly different ages, only the oldest material was used for calibration, although the younger material (stippled lines in figure 2; electronic supplementary material, figure S1) was taken into account for the diversity and disparity (but not the evolutionary rates) analyses (see below). By contrast, all extant taxa were fixed at 0 (i.e. the present), as they are mostly represented by modern-day DNA.

Following Ronquist *et al*. [16], we furthermore implemented two basal node calibrations using offset exponential distributions: one for the root, with a minimum of 39.5 Ma (based on the oldest pelagicete [31], *Zygorhiza* sp. from New Zealand; electronic supplementary material) and a mean of 54 Ma (based on the oldest cetacean, *Himalayacetus* [32]); and one for Neoceti, with a minimum of 34.2 Ma (based on the oldest neocete, *Llanocetus* [33–36]; electronic supplementary material) and a mean of 39.5 Ma (*Zygorhiza* sp.). The age of the oldest pelagicete remains controversial. A Late Lutetian or Early Bartonian age has been suggested for *Basilotritus wardii* from the Comfort Member of the Castle Hayne Formation of North Carolina and the Piney Point Formation of Virginia [37–41]. However, neither the age of the Castle Hayne Formation nor the horizon that has yielded material of this species is known with confidence [39,42]. To test the potential effects of assuming an earlier origin of Pelagiceti, we repeated our analysis using *B. wardii* instead of *Zygorhiza* sp. for both calibrations, with the former assigned an age of 42.9 Ma (base of calcareous nannoplankton zone NP16 [37,38,42,43]). Both calibrations resulted in similar divergence date estimates (table 1), and thus only the results based on *Zygorhiza* sp. are presented here.

**Table 1.** Divergence dates of major mysticete clades. Mean (*Z*) refers to the analysis calibrated using *Zygorhiza* sp., mean (*B*) that using *B. wardii* (Material and methods). Nodes in bold contain extant members in both descendant lineages and are hence directly comparable to purely molecular divergence date estimates. All dates are reported in Ma. HPD, highest posterior density; A, *Balaenoptera acutorostrata* + *B. bonaerensis*; B, *Balaenoptera* (except *B. acutorostrata* and *B. bonaerensis*) + *Megaptera* + *Eschrichtius*; C, *B. musculus* + *B. borealis* + *B. omurai*; D, *B. borealis* + *B. omurai*; E, *B. physalus* + *Megaptera* + *Eschrichtius*; F, *B. physalus* + *Megaptera.*

	this study				McGowen *et al*. [21]	
node	mean (*Z*)	95% HPD	mean (*B*)	95% HPD	mean	95% HPD
**Neoceti**	**38.80**	**42.26**–**35.02**	**39.38**	**43.31**–**36.00**	**36.36**	**40.14**–**34.24**
Mysticeti	38.42	41.72–34.90	39.00	42.68–35.86	—	—
Aetiocetidae	35.35	38.27–33.02	35.69	38.62–33.19	—	—
Chaeomysticeti	35.51	38.97–32.41	35.90	39.30–32.63	—	—
**crown Mysticeti**	**30.35**	**34.13**–**26.50**	**30.59**	**33.98**–**26.98**	**28.79**	**30.07**–**28.03**
Balaenidae	23.08	27.12–19.53	23.16	27.21–19.64	—	—
**crown Balaenidae**	**9.82**	**13.08**–**7.21**	**9.85**	**13.27**–**7.31**	**5.38**	**9.60**–**2.06**
**Plicogulae**	**28.96**	**32.63**–**25.76**	**29.22**	**32.92**–**26.13**	**22.59**	**28.64**–**15.35**
Cetotheriidae	25.24	29.34–21.42	25.51	29.71–21.75	—	—
Balaenopteroidea	28.18	31.57–25.40	28.48	31.97–25.59	—	—
Balaenopteridae	22.23	26.58–18.15	22.44	26.54–18.13	—	—
**crown Balaenopteridae**	**19.45**	**23.78**–**15.66**	**19.63**	**23.65**–**15.65**	**13.80**	**19.32**–**8.99**
**A**	**6.82**	**10.96**–**3.53**	**4.86**	**9.63**–**1.80**	**4.92**	**8.83**–**1.72**
**B**	**17.42**	**21.73**–**14.06**	**17.55**	**20.94**–**14.14**	**10.21**	**13.89**–**6.82**
**C**	**11.22**	**15.67**–**7.35**	**11.74**	**16.99**–**7.22**	**8.74**	**11.99**–**5.50**
**D**	**9.13**	**14.61**–**5.88**	**9.75**	**13.65**–**3.66**	**6.99**	**10.06**–**4.29**
**E**	**15.99**	**19.96**–**12.98**	**16.08**	**19.21**–**13.04**	**9.04**	**12.69**–**5.30**
**F**	**10.55**	**14.77**–**6.68**	**10.70**	**15.00**–**6.86**	**7.06**	**10.90**–**3.49**

After the implementation of all node and tip calibrations, we ran the analysis in MrBayes following the same settings (i.e. data partitions, substitution models, rates of change among morphological characters and coding bias) as described for the non-clock analyses above. Following Ronquist *et al*. [16] and Pyron [44], we used a single clock for both the molecular and morphological partitions. BFs strongly preferred an uncorrelated (igr) clock model over both an autocorrelated (tk02; BF∼108) and a strict (BF∼194) clock model. We retained the default setting for igrvarpr and set the clockratepr to a normal distribution with a mean of 0.01 and a standard deviation of 0.1. MCMC analyses were run for 50 million generations with the same settings as used for the non-clock analyses, assessed for convergence using the average standard deviation of split frequencies (all less than or equal to 0.12) and AWTY, and their results summarized in a majority-rule consensus tree showing all compatible clades.

### 3.4 Evolutionary rates

**Figure 3.**
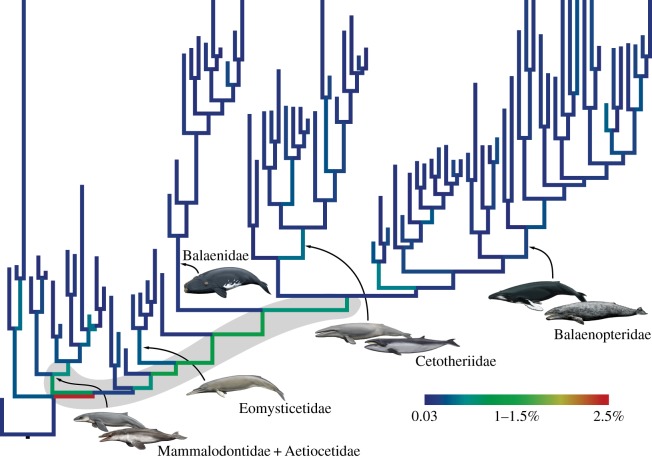
Combined phenotypic/genomic evolutionary rates. Branches are colour-coded by rate, with the fastest change occurring along the stem lineage (highlighted in grey) leading from the mysticete stem branch to all major clades. Topology, time scale and names are the same as in figure 2.

**Figure 4.**
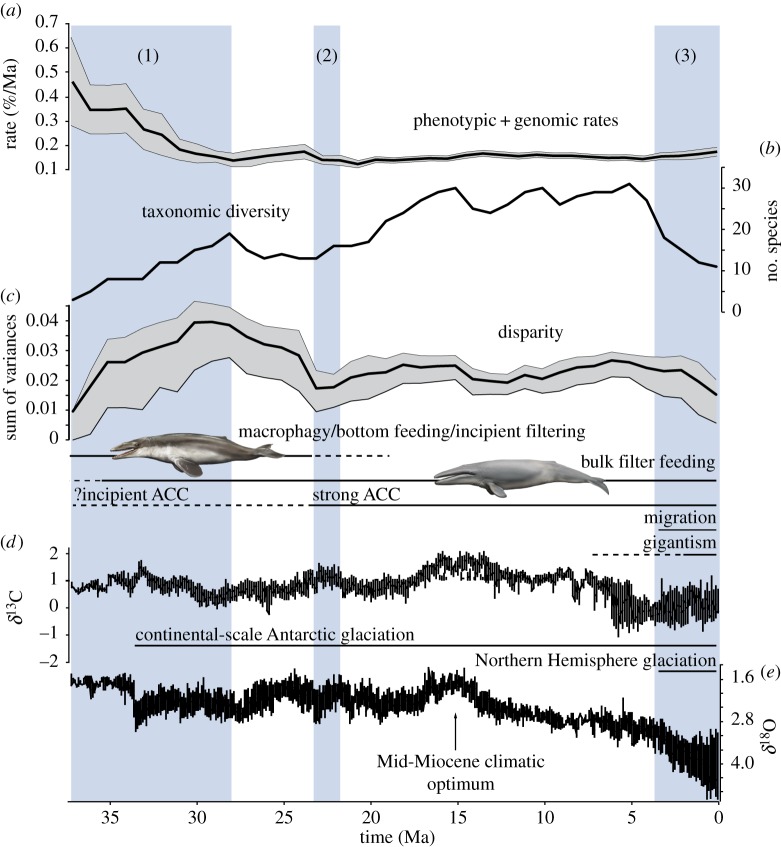
Major events in mysticete evolution driven by palaeoenvironmental change. High rates of phenotypic/genomic evolution (*a*) and an increase in taxonomic diversity (*b*) and disparity (*c*) mark an adaptive radiation during the initial phase of mysticete evolution (1), around the time of development of the ACC and the end of a global cooling trend. Rates and disparity subsequently decreased and then became stable from the Early Miocene onwards as the ACC developed its full strength and bulk filtering became the dominant feeding strategy (2). Diversity continued to rise and then remained stable during the Miocene, but markedly crashed towards the recent as the global climate deteriorated (*d*,*e*) and probably drove the final establishment of gigantism and, possibly, migration (3). Evolutionary rates represent the average of the mean rates for each branch in the dated total-evidence tree. Grey areas around the evolutionary rates and disparity curves represent the standard error of the mean and 95% confidence intervals (based on 1000 bootstrap replicates), respectively. Carbon and oxygen stable isotope data are from [45,46].

Evolutionary rates were calculated for mysticetes only. Because of our use of a single-clock model [16,44], phenotypic and genomic rates were estimated together as a single, averaged rate. Per-branch evolutionary rates quantify the amount of change from the initial divergence of the branch to the appearance of the morphology and/or genomic sequence represented by its tip. As the latter would be present with the oldest occurrence of the species in the fossil record, we did not consider any of the younger material excluded during the initial calibration of the fossil tips (see Total-evidence dating; figure 2). Because the relative rate estimates resulting from the dated total-evidence analysis were positively skewed, we relied on the median rates (as reported in the consensus tree) for comparisons and discussion [18]. We converted these relative rates into absolute ones (given as per cent change/Ma) through multiplying them by the median overall clock rate×100, and then (i) visualized the rate of each branch on the tree itself (figure 3) and (ii) calculated average rates across all lineages over the past 37 Ma in 1 Ma intervals (figure 4).

### 3.5 Diversity and disparity

As with evolutionary rates, diversity and disparity were calculated for mysticetes only. Palaeodiversity was quantified in the form of a lineage-through-time plot (in 1 Ma steps) of the fully dated tree, using the R [47] package Paleotree 2.0 [48]; disparity was calculated based on our morphological data matrix following Wills *et al*. [49] (figure 4). Disparity is a measure of morphological variation, expressed in terms of either the total range of occupied morphospace or the average dissimilarity between forms [49,50]. When explicitly linked to morphofunctional characters (e.g. those related to feeding), disparity can provide direct insights into functional morphology and ecology (e.g. [51]). In this study, we use all available character data to reconstruct disparity as a measure of overall body form [49]. Estimating disparity in this produces estimates that may correlate with, but are not necessarily always tied to, ecologically relevant adaptations. Nevertheless, they provide a reasonable outline of morphological evolution and avoid the need to make *a priori* choices regarding the relevance of particular characters.

To reconstruct mysticete disparity, we used our morphological dataset to derive a Euclidean distance matrix via the software Matrix v. 1.0 [49,50], which we then subjected to principal coordinates analysis (PCoA) in past v. 3.02 [52]. Next, we used the first six PCoA axes, cumulatively accounting for 95.3% of the total variance, to calculate the sum of variances per 1 Ma bin as an indicator of average dissimilarity between forms [49]. Finally, we calculated 95% confidence intervals based on 1000 bootstrap replicates of the same data (figure 4). All disparity calculations were performed in Rare v. 1.1 [49,50]. A variance-based measure of disparity was chosen because of its greater robustness to sample size biases, compared with estimates of range [53]. To test the possible influence of uneven sampling, we subsampled our data to the same level (*n*=8 species) across all 1 Ma time bins (based on 1000 bootstrap replicates). The disparity curve resulting from this reduced sample closely follows that derived from the full dataset (electronic supplementary material, figure S5), thus excluding sample size bias as a source of significant error in this case.

Because the baleen whale fossil record contains substantial gaps (e.g. during the Aquitanian, earliest Miocene, 23–20 Ma) and includes many taxa known only from fragmentary material, we applied three phylogenetic corrections based on our dated topology (figure 2): (i) following Butler *et al*. [53], missing data were replaced with inferred ancestral states; (ii) the range of each taxon was determined based on its duration (=ghost range) in the dated phylogenetic analysis; and (iii) internal branches were treated as hypothetical ancestors (=ghost lineages) and their reconstructed morphologies included in the disparity analysis (see also [54]). Ancestral states for all taxa and ghost lineages were reconstructed under parsimony in Mesquite v. 2.75 [55]. Only unambiguously reconstructed states were included, with the remainder being treated as missing data.

The inclusion of reconstructed character states reduced the amount of missing data from 42.9% (in the original data matrix) to just 6.8% in the phylogenetically adjusted matrix—a result remarkably similar to that of Butler *et al*. [53]. Note, however, that these values are not directly comparable, as the phylogenetically adjusted matrix also contained internal branches, and thus was nearly twice as large. To test the effect of phylogenetically adjusting our data, we also analysed a much less resolved, stage/sub-epoch-level taxic dataset, comprising only the scored taxa according to their sampled stratigraphic ranges (electronic supplementary material) without taking into account ghost ranges/lineages or reconstructed missing data. The pattern of ‘raw’ disparity calculated from this dataset (electronic supplementary material, figure S6) broadly follows that of the phylogenetically adjusted one (figure 4), with an Early Oligocene peak followed by lower levels in the Miocene and Pliocene, although Late Miocene levels in particular are relatively somewhat higher.

## 4. Results

### 4.1 Phylogeny

To assess the effect of forcing our data to evolve under a clock model, we conducted both dated (figure 2; electronic supplementary material, figure S1) and non-dated analyses (electronic supplementary material, figures S2 and S3), with similar results. At the family level, all of the resulting tree topologies are largely consistent with previous molecular studies [5,7,9,11,21,56], independent of whether the morphological data were analysed as part of the total-evidence matrix (figure 2; electronic supplementary material, figures S1 and S2) or on their own (electronic supplementary material, figure S3). The same is, however, not true for balaenopterids (electronic supplementary material, figure S3), where the results of our morphological analysis contradict published molecular hypotheses (e.g. [5,21]). By contrast, the total-evidence analyses recovered a topology that is identical to that proposed by McGowen *et al.* [21], and therefore probably dominated by the molecular data.

In line with some previous studies [10,34], our results recognize at least two basal clades of archaic toothed mysticetes (Aetiocetidae and Mammalodontidae). As also found by Steeman *et al*. [33], *L. denticrenatus*, the oldest reported baleen whale (Late Eocene, Antarctica), is the sister taxon of toothless mysticetes, Chaeomysticeti. The latter comprise a monophyletic Eomysticetidae, an undescribed new species from New Zealand (OU 22224, previously provisionally identified as a balaenid [57]) and crown Mysticeti. The position of two undescribed, archaic toothed mysticetes from Charleston, USA and New Zealand is less stable, with both of them either forming the sister to *Llanocetus*+ Chaeomysticeti (figure 2) or falling close to the base of Mysticeti as a whole (electronic supplementary material, figures S2 and S3), as in previous analyses [9,34].

Balaenidae (right whales) are monophyletic, and basal to the rest of the crown Mysticeti (Plicogulae *sensu* [9]), including balaenopterids (rorquals), eschrichtiids (grey whales) and neobalaenines (pygmy right whales). Within Plicogulae, cetotheriids are monophyletic and include neobalaenines. Eschrichtiids also form a well-defined clade nested within balaenopterids (figure 2), although only the total-evidence analyses point to a potentially paraphyletic crown Balaenopteridae. In addition, our analysis revealed a further two clades of mostly Miocene taxa, comprising: (i) *Mauicetus parki*, *Aglaocetus moreni* and an undescribed new species from New Zealand (ZMT 67); and (ii) *Diorocetus chichibuensis*, *Tiphyocetus temblorensis*, *Isanacetus laticephalus*, as well as four species referred to *Parietobalaena* and an undescribed taxon from New Zealand (OU 22705). A third clade including *Aglaocetus patulus*, *Diorocetus hiatus*, *Thinocetus arthritus*, *Pelocetus calvertensis* and *Uranocetus gramensis* only occurs in the dated analysis (figure 2), although the association of *D. hiatus* and *T. arthritus*, identified earlier [33], remains stable throughout (figure 2; electronic supplementary material, figures S2 and S3). Some of these groupings—especially that including *Parietobalaena*—may indicate the existence of previously unrecognized family-level taxa, clustering with rorquals to form an expanded Balaenopteroidea. Low posterior probabilities, as well as the generally unstable position of these Miocene taxa in different analyses, however, caution against using formal families for now.

### 4.2 Divergence dates

Mysticetes diverged from odontocetes around 38.8 Ma (late Middle Eocene), followed by the divergence of chaeomysticetes from *Llanocetus* around 36.6 Ma (Late Eocene), balaenids from the other members of the crown group around 30.4 Ma and cetotheriids from Balaenopteroidea around 28.96 Ma (late Early Oligocene). Pliocogulae and crown balaenopterids (including eschrichtiines) may have diverged as early as 19.5 Ma, but low branch support makes their origins uncertain (figure 2). On the whole, these figures are consistent for two different basal calibrations for Neoceti (table 1) and pre-date some recent molecular estimates by 2–5 Ma [5,21,56]. However, several studies have reported similar age ranges for Neoceti [23], crown Mysticeti [23] and crown Balaenopteridae [5,58,59]. Our estimates are consistently older than those of McGowen *et al.* [21], but generally fall within, or just outside, the 95% highest posterior density (HPD) ranges of that study. The only exceptions to this are two nested clades within crown Balaenopteridae (table 1, B and E), which are 3–4 Ma older than their equivalent 95% HPD ranges in McGowen *et al.* [21].

### 4.3 Macroevolutionary dynamics

Combined phenotypic/genomic rates of evolution are fastest near the base of the tree and remain relatively high—around five to six times the median overall clock rate (0.18%)—along the entire mysticete stem lineage (grey area in figure 3). This finding parallels similar patterns in birds [60] and arthropods [61]. As in past studies of archosaurs and of other groups of mammals [54], the inclusion of ghost lineages increases the occupied morphospace beyond that defined by the sampled taxa alone (electronic supplementary material, figure S4). Throughout the entire history of the clade, evolutionary rates and disparity are decoupled from taxonomic diversity (figure 4). Evolutionary rates and disparity were high just after the latest Eocene origin of mysticetes. Disparity rose to an early, pronounced peak around 30–29 Ma (late Early Oligocene). Rates and disparity later decreased to a relatively stable level around 23 Ma (earliest Miocene). By contrast, taxonomic diversity increased steadily until 15 Ma (with a minor peak around 28 Ma) and then remained high, albeit fluctuating, until *ca* 4 Ma, before sharply declining towards the present. This pattern sharply contrasts with previously published lineage-through-time plots based on extant taxa only (which can never decrease) [5,62], as well as modelled patterns of diversity (including extinction) inferred from purely molecular phylogenies [63]. These discrepancies highlight the importance of including fossil taxa directly into macroevolutionary analyses.

## 5. Discussion and conclusion

Our phylogeny is largely congruent with the results of previous molecular studies [5,19,52] and highlights the Late Oligocene–Early Miocene as a time of transition from toothed mysticetes and archaic chaeomysticetes to the rise of the modern families. The monophyly of Plicogulae contradicts most previous morphological studies [8,33,64–66], but is well supported by molecular results [5,9,11,21,56], thus lending credence to the previously suggested inclusion of neobalaenines within Cetotheriidae [7,67]. The nesting of eschrichtiids within crown Balaenopteridae is more controversial. Some molecular studies have supported such a relationship [21,56], but others [5,24], and most morphological analyses [8,10,33,64], instead found rorquals to be monophyletic. Nevertheless, all available evidence strongly supports an extremely close relationship between eschrichtiids and rorquals, as is evident from (i) the general dispute about balaenopterid paraphyly, (ii) the lack of support for eschrichtiid monophyly (relative to balaenopterids) in at least one previous study [11]; and (iii) the inclusion of the much more balaenopterid-like ‘*Balaenoptera*’*portisi*[68] within Eschrichtiidae, as suggested by our results (figure 2). If the nesting of grey whales within crown balaenopterids turned out to be wrong, our analysis would still indicate that grey whales are more closely related to living rorquals than certain balaenopterid stem taxa (e.g. ‘*B*.’ *ryani*). Further evidence to support this view comes from the recent description of the bony labyrinth of a range of living and fossil mysticetes, which demonstrated the occurrence of a tympanal recess in eschrichtiids and crown balaenopterids, but not ‘*Megaptera*’*miocaena* [69]. This complements our results, which consistently interpret ‘*M*.’ *miocaena* as basal relative to both living rorquals and grey whales (figures 2; electronic supplementary material, figures S2 and S3). We therefore suggest that Eschrichtiidae should be lowered to the rank of subfamily (hereafter Eschrichtiinae).

Recent comparisons have shown that morphological clock analyses may overestimate divergence dates owing to the generally higher rate heterogeneity of morphological datasets [18]. This has sometimes resulted in divergence dates which were substantially (and sometimes implausibly) younger or older than purely molecular estimates and/or evidence from the fossil record [18,30,61,70], possibly as a result of poorly understood, or inadequately modelled, temporal variations in evolutionary dynamics. Unlike in the latter studies, our estimates are broadly comparable with those of purely molecular (node-calibrated) analyses, and only modestly pre-date the fossil record. In particular, our date estimates are relatively close to those of McGowen *et al*. [21], who originally provided the molecular data analysed here. This may be a sign that the molecular and morphological data are relatively congruent. The greater age of two balaenopterid ‘outlier’ clades is most likely explained by their inclusion of grey whales (figure 2), which are morphologically disparate from other rorquals and thus push back associated morphological clock estimates. Nevertheless, some of our inferred dates imply a substantial unsampled evolutionary history, which might reflect (i) the globally meagre record of named fossil cetaceans from the (Early) Oligocene and earliest Miocene (figure 2); and (ii) the poorly understood origins of Neoceti from within ‘Archaeoceti’, owing to a dearth of morphologically conservative odontocetes that could help to illuminate ancestral neocete morphology. Odontocete and even chaeomysticete material of reported latest Eocene and earliest Oligocene age [71,72] could fill in some of these gaps once the fossils are described formally.

Our phylogeny suggests three major events in mysticete evolution: (i) an early adaptive radiation lasting from *ca* 39 to 28 Ma; (ii) a shift towards bulk filter-feeding as the sole feeding strategy around 23 Ma; and (iii) a marked, climate-driven drop in diversity around 3 Ma. The origin of mysticetes coincides with a long-term cooling phase later in the Eocene, culminating in a rapid temperature drop and initiation of Antarctic glaciation near the Eocene/Oligocene boundary. These events were coeval with the gradual development of a (shallow) precursor of the modern Antarctic Circumpolar Current (ACC) [73]. Our linking of baleen whale evolution and climatic/oceanic change supports previous studies that argued for the ACC as a driver of whale origins through increased ocean mixing, and hence nutrient availability [4,6,74]. However, the time of onset of the ACC remains controversial [75,76], and it is not clear when the Drake Passage and Tasmanian gateway became deep enough to allow the deep mixing that drives ACC-related productivity today [4,77].

As cooling continued, toothed mysticetes diversified and chaeomysticetes originated, at a time of abrupt eutrophication of the Southern Ocean around 37–36 Ma (figure 2)—about 2.5 Ma before the establishment of a permanent, continental-scale Antarctic ice sheet [78]. The initially high phenotypic/genomic rates and a concurrent increase in both diversity and disparity (until about 28 Ma; figure 4) are consistent with an early adaptive radiation, which is further supported by elevated rates of speciation early in cetacean evolutionary history [79]. The relatively gentle rise in diversity during the Early Oligocene is less marked than might be expected for a radiation event, but is likely an artefact of poor sampling [6,80]: the currently minor Oligocene diversity peak will become more pronounced—and older—as more material is described. After 28 Ma, disparity started to decline and rates began to level out, which may reflect the effects of ecological niche filling [81] and the gradual rise to dominance of baleen-assisted filter-feeding. The overall slowdown in phenotypic/genomic rates resembles a similar drop in the speciation rate of extant cetaceans, which may point to a potential correlation of these two variables [82]. We are currently unable to test this idea, because the method used to reconstruct the rate of speciation in living cetaceans does not yet extend to phylogenies including fossils [79].

A slowdown in the rate of morphological evolution over time was also recovered in a previous study that included both mysticetes and odontocetes, but focused on extant taxa and body size only [62]. In addition, this analysis demonstrated a link between body size evolution and feeding strategy, and proposed an early partitioning of the major ecological niches occupied by cetaceans today. Our study suggests that mysticetes probably evolved along similar lines. Thus, the Eocene/Oligocene radiation produced a range of distinct morphotypes and feeding strategies, which led to the coeval Oligocene occurrence of bottom-feeding [34], macrophagous [83] and possibly filter-feeding [11] toothed mysticetes, as well as several toothless forms (eomysticetids, *M. parki* and OU 22224; figure 2). Interestingly, this ecological diversity was accompanied by considerable disparity in body size, as illustrated by the diminutive mammalodontids and aetiocetids, the medium-sized eomysticetids and some rare examples of relatively large taxa (e.g. *Llanocetus*). Further work is needed to test whether this apparent partitioning of size niches early in mysticete evolution indeed reflects ecology, as in the case of extant cetaceans.

Around the time of the Oligocene/Miocene boundary (23 Ma), both evolutionary rates and disparity became essentially static, and largely remained so until the present. Concurrently, the varied Oligocene mysticete assemblage was replaced by a more ‘modern’-looking (i.e. balaenid/stem-balaenopteroid dominated) range of chaeomysticetes, in tandem with increased levels of localized productivity probably associated with a strengthening ACC [75,76]. We speculate that the stabilization of disparity after 23 Ma may be the result of two independent processes: (i) the decline to extinction of toothed mysticetes, which effectively excluded the clade as a whole from niches unrelated to baleen-assisted filter-feeding; and (ii) the persistence of a relatively stable set of niches that mysticetes could exploit, with no major new adaptations that could have resulted in ecological opportunity (unlike in delphinids) [62,79].

While an essentially modern ACC probably created favourable conditions for bulk feeders [4], it is not clear what might have caused the demise of the toothed forms. Competition with other groups, especially odontocetes and pinnipeds, may have played a role, although the different structure of the feeding apparatus in toothed mysticetes, odontocetes and pinnipeds would suggest little direct morphofunctional overlap. Odontocetes in particular may increasingly have been at an advantage as their echolocation abilities continued to evolve [84]. If so, the disappearance of toothed mysticetes could represent the final replacement of the ‘archaeocete’-like (non-filter-feeding, non-echolocating) morphotype, following which pelagic marine ecospace became finally partitioned between filter-feeding baleen whales and echolocating odontocetes. The end of the Eocene/Oligocene mysticete radiation also coincides with the first appearance of fossil pinnipeds [85], which was soon followed by the divergence of phocids from otarioids during the Late Oligocene or earliest Miocene [86,87]. The North Pacific is noteworthy for its diverse record of aetiocetids [88–90], and its near-coeval role as a cradle of early pinniped evolution [91]. A potential ecological overlap of aetiocetids and early pinnipeds deserves study, given the small size of aetiocetids and their continuing reliance on teeth as part of their feeding strategy [11].

Unlike disparity, mysticete diversity continued to increase markedly until about 15 Ma, and then remained relatively stable, with a plateau until 4–3 Ma. The onset of the plateau at 15 Ma reflects the comparatively early diversification of the balaenopterids (figure 2), albeit with poorly supported interrelationships. The speciose but poorly understood clade centred on *Parietobalaena* also contributes to elevated levels of diversity around this time. This is potentially problematic, since most of the included species suffer taxonomic shortcomings. For example, the holotype of *Parietobalaena palmeri* is a juvenile, and many of the referred specimens are insufficiently studied; all described material attributable to *Parietobalaena campiniana* and *Tiphyocetus* is fragmentary and, in the case of *P. campiniana*, juvenile; and finally, the taxonomically informative periotic is insufficiently known in *Parietobalaena* sp. from Japan, *Parietobalaena yamaokai*, *Diorocetus chichibuensis* and *Pinocetus polonicus*. More detailed taxonomic studies may reveal some of these taxa to belong to the same species. This, and/or a younger date of divergence for crown Balaenopteridae (say, 14–13 Ma [21,23]) would flatten the Early Miocene portion of the curve and shift the peak diversity to the Late Miocene–Early Pliocene.

We note that the onset of the diversity plateau coincides with pronounced peaks in the oxygen and carbon isotopic records (*ca* 16–15 Ma) marking the Mid-Miocene Climatic Optimum [45,92]; however, we also caution against literal interpretation of the diversity curve (in the manner of Marx & Uhen [74]) for now, pending further insights into the origins and taxonomy of balaenopterids and *Parietobalaena*-like taxa, as well as more complete sampling of the mysticete fossil record. Of particular interest are the poorly sampled Early Oligocene, which saw the initial diversification of baleen whales, and the earliest Miocene (Aquitanian; *ca* 23–20 Ma). No mysticete species has yet been described from the latter 3 Ma interval (figure 2; electronic supplementary material), even though inferred diversity was high (figure 4). We predict that further discoveries of archaic species from the latest Eocene and Early Oligocene will result in a relative increase in early mysticete diversity and, possibly, disparity, starting around the Eocene/Oligocene boundary. We furthermore anticipate that the Early Miocene transition will ultimately appear less abrupt (but remain distinct), owing to the survival of at least some archaic mysticete lineages (aetiocetids and eomysticetids) past the Oligocene/Miocene boundary [93].

We interpret the sharp decline in diversity around 3 Ma as real, because of (i) its magnitude; (ii) a similar, albeit much smaller, decrease in disparity; and (iii) the concurrent decline or disappearance of small-sized forms (e.g. *Balaenula*, *Balaenella*, herpetocetines and several small balaenopterids; figure 2), which established gigantism as the dominant mysticete size habit after its origin during the Late Miocene [94–96]. It is striking that these changes coincided with the onset of long-term Northern Hemisphere glaciation [97]. The latter plausibly impacted mysticete diversity, disparity, body size and food distribution through changes in continental shelf habitats. It has been suggested that glacial cycles caused repeated loss or reduction of available shelf area [98]. Glacioeustatic cycles might better be seen, however, as causing rapid change in distribution of habitat rather than repeated loss, as each low-stand would not eliminate the shelf but would remobilize sediments to prograde the shelf basin-ward. Such changes would indeed impact more on smaller neritic species than on larger pelagic forms. Further, wind transfer of glacially generated, more bioavailable [99] iron-rich dust into oceanic settings could have fertilized phytoplankton at the base of the mysticete food chain [100]. Increased glacial productivity primarily occurred in the sub-Antarctic Ocean [101,102], whereas other high nutrient, low chlorophyll areas, such as the equatorial Pacific, seem to have experienced no change or even a decline in productivity [103]. The disappearance of small-sized mysticetes and resulting dominance of their larger cousins could therefore be the result of a greater need to migrate between high-latitude feeding and low-latitude breeding habitats, the distance between which repeatedly changed as the polar fronts moved closer to the equator or the poles [104]. This hypothesis may be supported by evidence of baleen whale migration stretching back to at least the Late Pliocene [105].

## Supplementary Material

Supplementary figures, stratigraphic review, list of studied material, supplementary references and list of morphological characters.

## Data accessibility

Morphological and total-evidence data matrices are available from MorphoBank (www.morphobank.org), project 687.
