# Supplementary material for: Baleen boom and bust: a synthesis of mysticete phylogeny, diversity and disparity
Source: R Soc Open Sci. 2015 Apr 15;2(4):140434. doi: 10.1098/rsos.140434 (PMC4448876; doi:10.1098/rsos.140434)
Supplement: Supplementary figures, stratigraphic review, list of studied material, supplementary references and list of morphological characters. [file rsos140434supp1.pdf]

Supplementary Material

Marx FG, Fordyce, RE. Baleen boom and bust – a synthesis of mysticete phylogeny, diversity and disparity. *Royal Society Open Science*

Contents

Figs. S1 to S6

Table S1

Table S2

Review of stratigraphic ranges

Institutional abbreviations

List of studied material

Supplementary references

Morphological characters

Note: The morphological data matrix and all images associated with this paper are available from MorphoBank (www.morphobank.org), project 687. A Nexus file containing the full supermatrix and MrBayes codes for carrying out the total evidence dating analysis are also available, and stored in the "Documents" section of the same project.

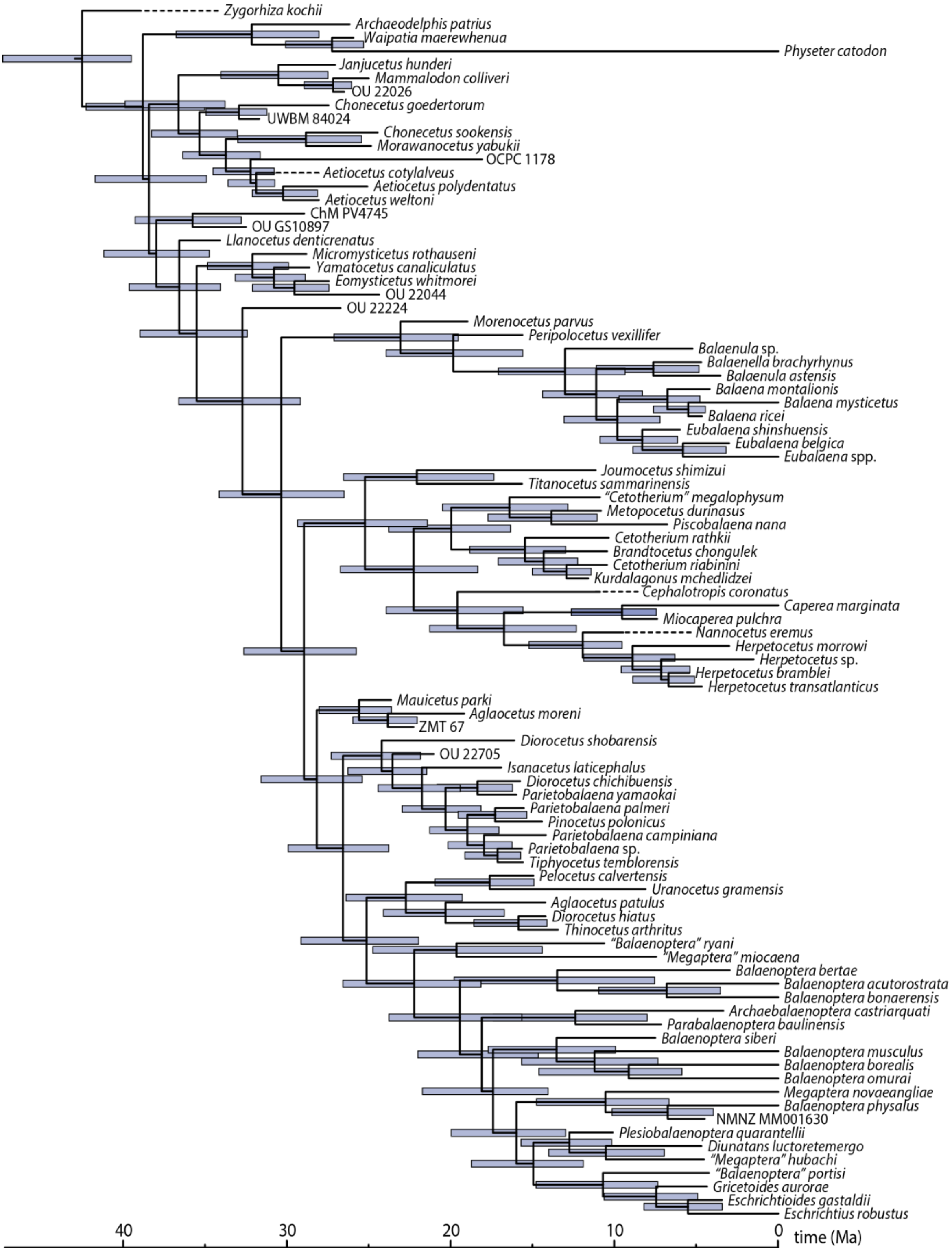

Fig. S1. 95% highest posterior density (HPD) intervals. Majority-rule consensus tree (showing all compatible clades; "allcompat" option in MrBayes) resulting from the total evidence dating analysis. Stippled lines represent fossil material postdating the oldest occurrence of a given species (see Methods and review of stratigraphic ranges below). Error bars indicate the 95% highest posterior density for the age of each node.

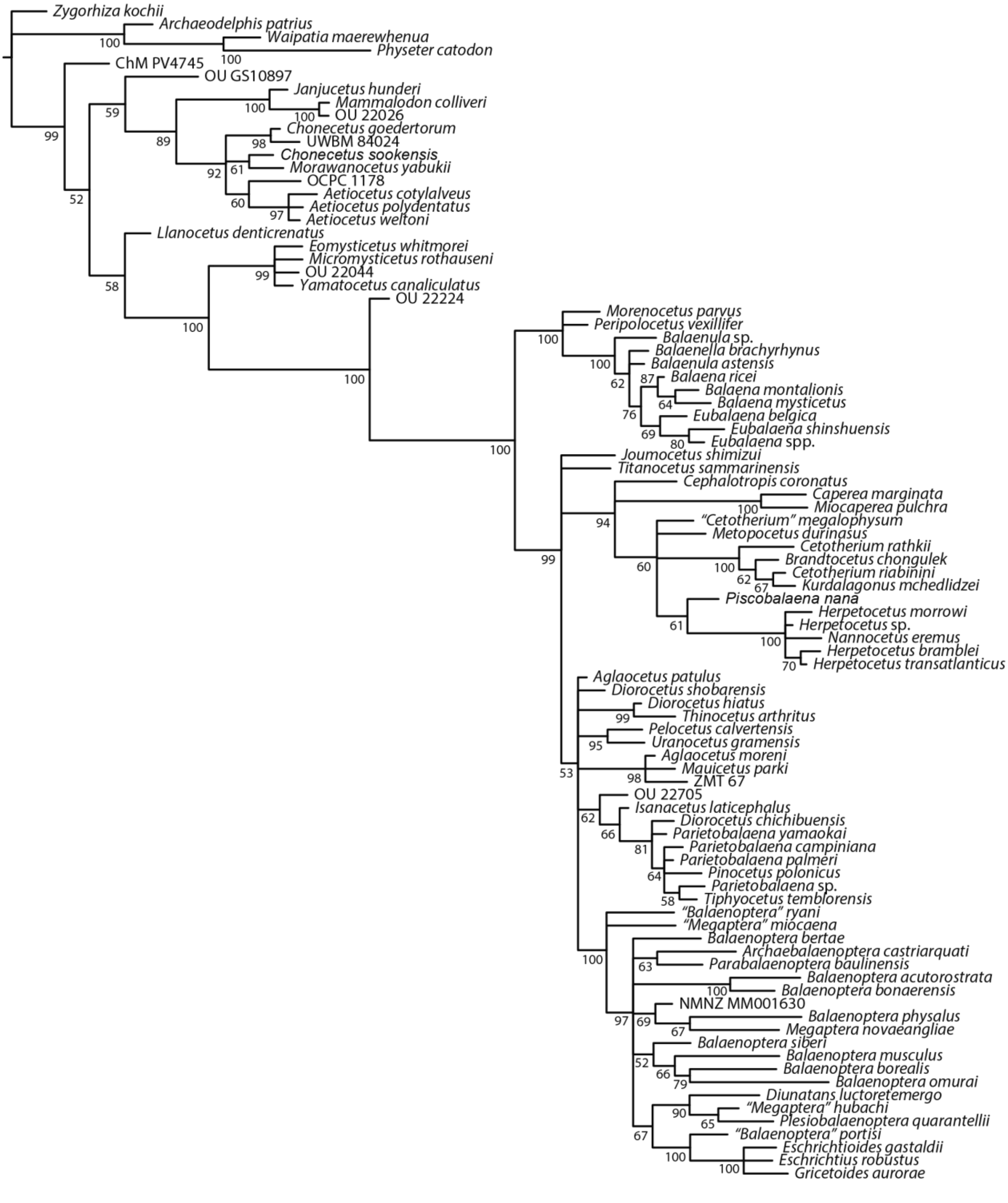

Fig. S2 50%-majority rule consensus tree resulting from the analysis of the supermatrix only, without inferring divergence dates. Numbers next to nodes reflect the posterior probabilities of individual clades.

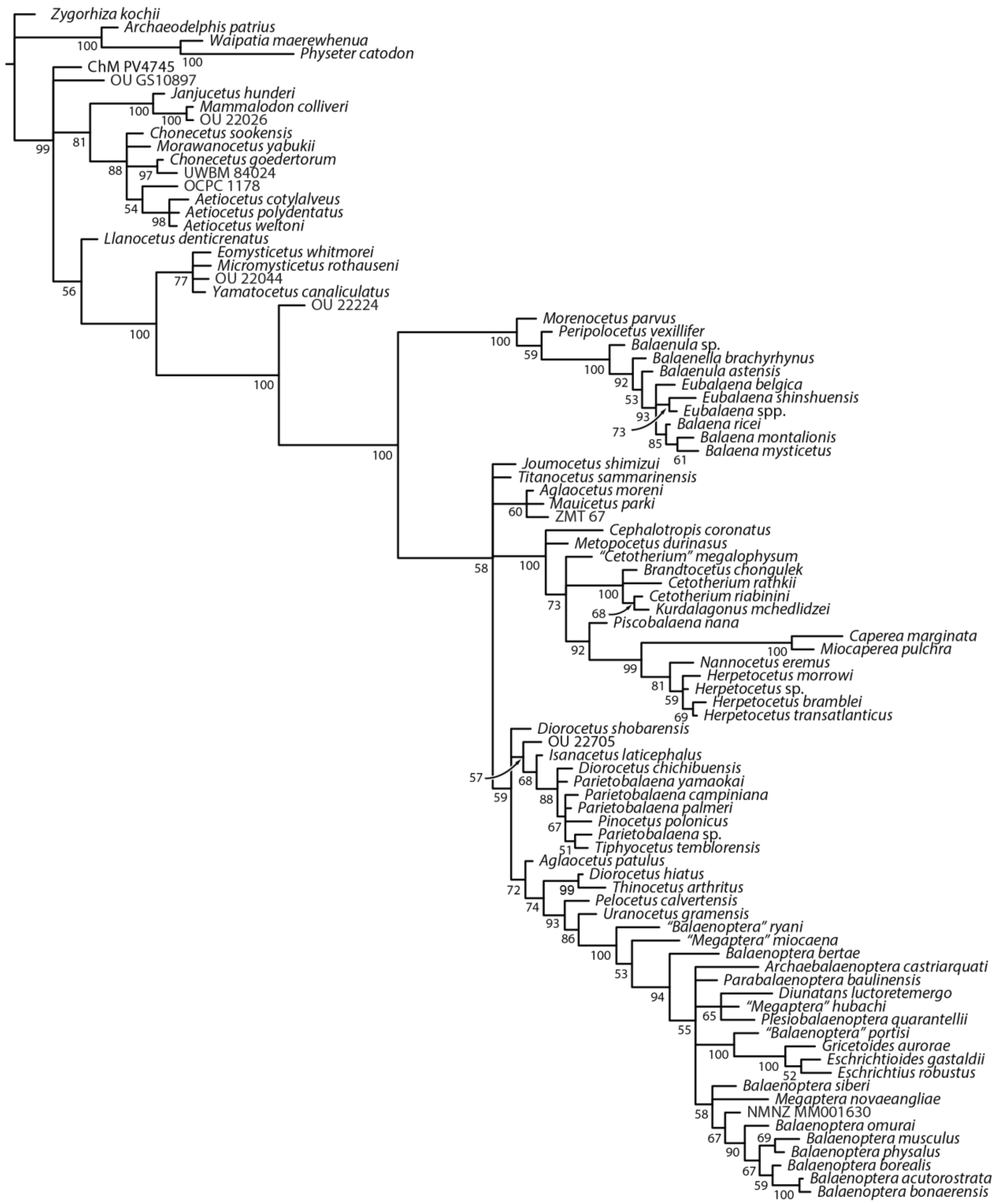

Fig. S3 50%-majority rule consensus tree resulting from the analysis of the morphological data only, without inferring divergence dates. Numbers next to nodes reflect the posterior probabilities of individual clades.

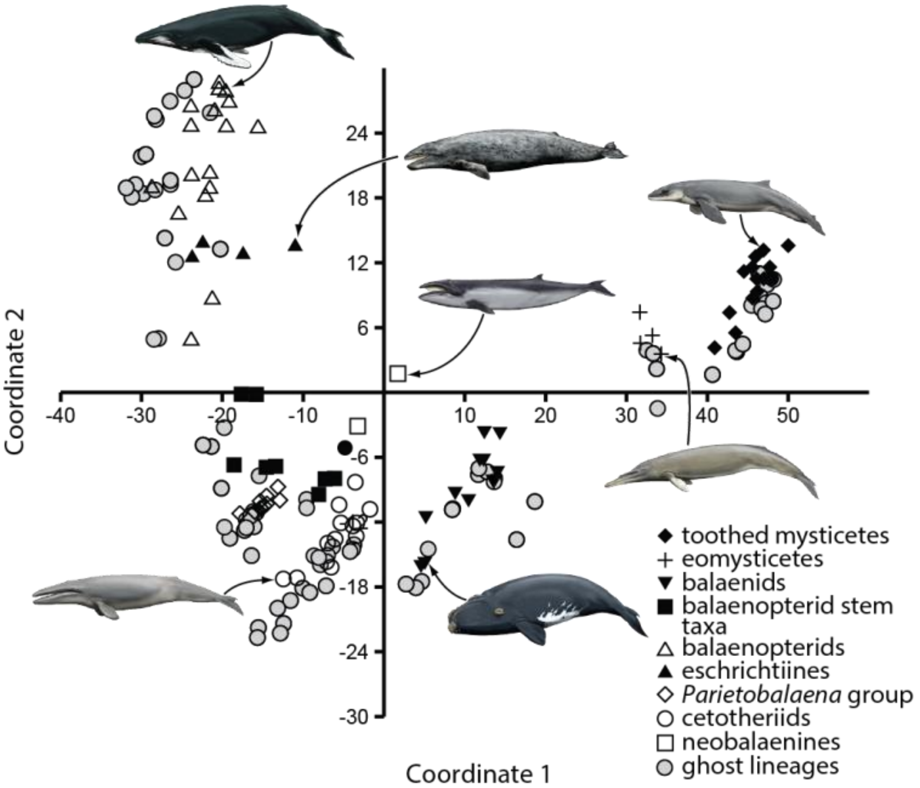

Fig. S4 Baleen whale morphospace. Visualisation of mysticete morphospace as the first 2 axes of a Principal Coordinates Analysis taking into account reconstructed missing data, ghost ranges, and ghost lineages. Note that ghost lineages (hypothetical ancestors) repeatedly fall outside the area defined by the observed taxa themselves, thus increasing the range of sampled morphologies.

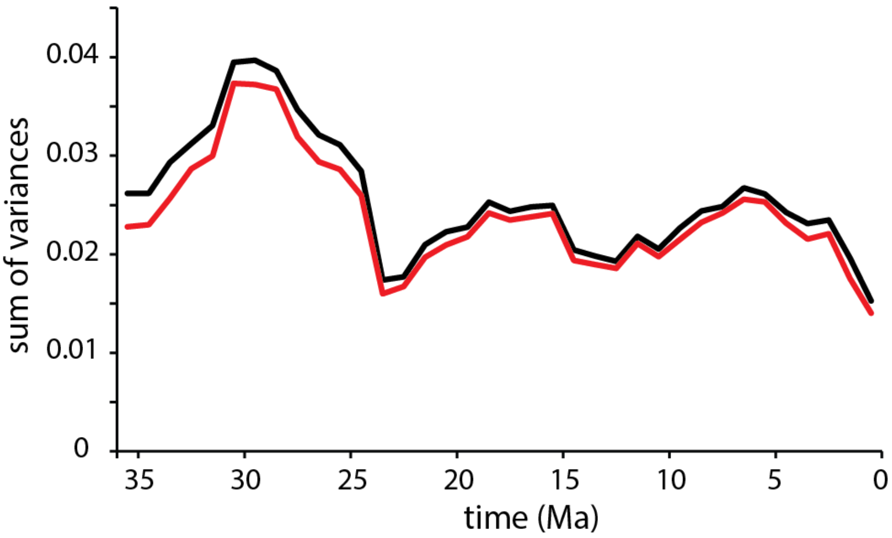

Fig. S5 Comparison of absolute and subsampled disparity. Black line represents disparity calculated based on all available species per 1 Ma time bin. Red line shows disparity subsampled to 8 species per 1 Ma time bin, based on 1000 bootstrap replicates

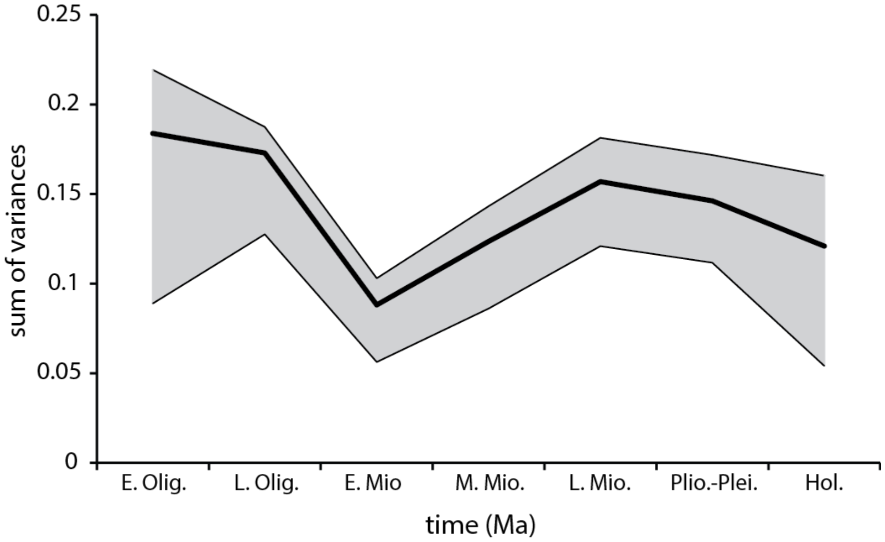

Fig. S6 Raw disparity based on the purely taxic dataset. Disparity was calculated in the same way as for the phylogenetically adjusted data, but based on a less resolved dataset comprising only the scored taxa according to their sampled stratigraphic ranges (Methods; Supplementary Material). Abbreviations: E. Olig., Early Oligocene; L. Olig. Late Oligocene; E. Mio, Early Miocene; M. Mio., Middle Miocene; L. Mio, Middle Miocene; Plio-Plei., Plio-Pleistocene; Hol., Holocene.

Table S1. Stratigraphic ranges of included fossil taxa. Where two ranges are shown, those in parentheses were used for the total evidence dating analysis (see Methods and review of stratigraphic ranges below).

Taxon	Age	Age (Ma)	References
**Outgroup**			
Zygorhiza sp.	late Bartonian–Priabonian	39.5–34.0 (39.5–38.4)	see below
Archaeodelphis patrius	early–late Chattian	27.2–24.5	see below
Waipatia maerewhenua	early–late Chattian (Duntroonian)	27.3–25.2	[1, 2]
**Ingroup**			
Aetiocetus cotylalveus	late Rupelian–early Chattian	31.0–28.0 (31.0–30.6)	see below
Aetiocetus polydentatus	early–late Chattian	26.1–23.3	see below
Aetiocetus weltoni	early Chattian	28.1–28.0	see below
Aglaocetus moreni	early Burdigalian	19.8–18.2	see below
Aglaocetus patulus	Langhian	14.5–13.9	see below
Archaebalaenoptera castriarquati	Piacenzian	3.6–3.1	[3]
Balaena montalionis	Zanclean	4.5–3.9	[4]
Balaena ricei	Zanclean	4.9–4.4	see below
Balaenella brachyrhynus	Zanclean	5.0–4.4	see below
Balaenoptera bertae	Piacenzian	3.4–2.5	[5]
“Balaenoptera” portisi	Zanclean–Piacenzian	5.3–3.0	see below
“Balaenoptera” ryani	early Tortonian	11.6–9.5	see below
Balaenoptera siberi	late Tortonian	8.0–7.0	see below
Balaenula astensis	Zanclean–Piacenzian	4.0–3.0	see below
Balaenula sp.	Messinian–Zanclean	6.4–4.1	see below
Brandtocetus chongulek	early Tortonian	11.2–9.6	see below
Cephalotropis coronatus	early–late Tortonian	11.7–8.5 (11.7–10.0)	see below
“Cetotherium” megalophysum	early Tortonian	11.7–10.0	see below
Cetotherium rathkii	early Tortonian	11.2–9.6	see below
Cetotherium riabinini	early Tortonian	11.2–9.6	see below
ChM PV4745	late Rupelian	29.6–28.1	see below
Chonecetus goedertorum	early Chattian	28.1–26.5	see below
Chonecetus sookensis	late Chattian	24.8–24.1	see below
Diorocetus chichibuensis	late Burdigalian–Langhian	16.4–15.1	see below
Diorocetus hiatus	Langhian	14.5–13.9	see below
Diorocetus shobarensis	late Burdigalian–Langhian	17.0–14.9	see below
Diunatans luctoretemergo	Zanclean	5.0–4.4	[6]
Eomysticetus whitmorei	early Chattian	28.1–26.8	see below
Eschrichtioides gastaldii	Zanclean–Piacenzian	4.0–3.0	see below
Eubalaena belgica	Piacenzian	3.2–2.8	see below
Eubalaena shinshuensis	Messinian	6.1–5.9	see below
Gricetoides aurorae	Zanclean	4.9–3.9	see below
Herpetocetus bramblei	Messinian–Zanclean	6.4–4.9	see below

Herpetocetus morrowi	Piacenzian	3.5–2.5	[7]
Herpetocetus sp.	Pleistocene (incl. Gelasian)	2.1–0.7	[8]
Herpetocetus transatlanticus	Zanclean	4.9–4.4	see below
Isanacetus laticephalus	late Burdigalian	17.5–16.0	see below
Janjucetus hunderi	early? Chattian	28.1–25.6	see below
Joumocetus shimizui	early Tortonian	11.3–11.0	see below
Kurdalagonus mchedlidzei	Serravallian–early Tortonian	12.1–11.2	see below
Llanocetus denticrenatus	late Priabonian	34.2–34.0	see below
Mammalodon colliveri	late Chattian	25.7–23.9	see below
Mauicetus parki	late Chattian	25.2–23.0	see below
"Megaptera" hubachi	Zanclean	5.3–3.6	see below
"Megaptera" miocaena	late Tortonian	7.6–7.3	see below
Metopocetus durinasus	early Tortonian	11.7–10.0	see below
Micromysticetus rothauseni	late Rupelian	29.6–28.1	see below
Miocaperea pulchra	late Tortonian	7.5–7.3	see below
Morawanocetus yabukii	early–late Chattian	26.1–23.3	see below
Morenocetus parvus	early Burdigalian	19.8–18.2	see below
Nannocetus eremus	late Tortonian–Messinian	10.0–5.3 (10.0–9.0)	see below
NMNZ MM001630	Zanclean	5.3–3.6	see below
OCPC 1178	early-late Burdigalian	18.8–17.2	see below
OU 22026	early Chattian	27.3–26.0	see below
OU 22044	late Chattian	25.4–25.3 (25.2–23.0)	see below
OU 22224	early Chattian	27.3–26.0	see below
OU 22705	Aquitanian	21.7–20.5	see below
OU GS10897	early Rupelian	33.0–32.0	see below
Parabalaenoptera baulinensis	Late Tortonian–Messinian	7.6–6.7	see below
Parietobalaena campiniana	Langhian–Serravallian	15.0–13.2	[9]
Parietobalaena palmeri	late Burdigalian–Langhian	16.4–14.5	see below
Parietobalaena sp.	late Burdigalian–Langhian	16.4–15.1	see below
Parietobalaena yamaokai	late Burdigalian–Langhian	17.0–14.9	see below
Pelocetus calvertensis	Langhian	15.5–14.5	see below
Peripolocetus vexillifer	Langhian	16.0–15.2	see below
Pinocetus polonicus	Langhian	15.1–13.8	see below
Piscobalaena nana	late Tortonian–Messinian	7.5–5.9	see below
Plesiobalaenoptera quarantellii	early Tortonian	11.6–9.4	see below
Thinocetus arthritus	Serravallian	13.5–13.4	see below
Tiphyocetus temblorensis	Langhian	16.0–15.2	see below
Titanocetus sammarinensis	late Burdigalian–Langhian	16.4–14.7	see below
Uranocetus gramensis	late Tortonian	8.6–7.5	see below
UWBM 84024	early Rupelian	33.2–31.0	see below
Yamatocetus canaliculatus	late Rupelian	29.2–28.1	see below
ZMT 67	Aquitanian	23.0–21.7	see below

Review of stratigraphic ranges

(1) Outgroup

Archaeodelphis patrius

Specimen: MCZ 15749 (holotype) [10].

Locality and horizon: Charleston, South Carolina, USA: Tiger Leap Formation [10, 11].

Stratigraphic range: early–late Chattian (27.2–24.5 Ma).

Comments: Sediment preserved with the skull has yielded a dinoflagellate assemblage including *Saturnodinium pansum* and *Pentadinium imaginatum* [11; M. Uhen 2012, pers. comm.]. The overlap of these two species is defined by the first occurrence of *P. imaginatum* at the base of NW European dinoflagellate cyst zone D15c (ca. 27.2 Ma) [12] and the last occurrence of *S. pansum* at Northern Hemisphere mid-latitudes around 24.5 Ma [13].

***Zygorhiza* sp.**

Specimens: OU 22100, 22222-1, 22242 [14]; note that this list only applies to *Zygorhiza* sp. from New Zealand, and does not represent a comprehensive account of all specimens referred to the genus.

Locality and horizon: Waihao Forks, near Waimate, South Canterbury, New Zealand: Lower Greensand Member of the Waihao Greensand [14].

Stratigraphic range: late Bartonian–Priabonian (39.5–34.0 Ma).

Comments: At least OU22242, and likely all of the specimens, originate from a horizon located immediately below a phosphatised, concretionary layer marking the top of the Lower Greensand Member [14]. The age of this deposit is constrained by the occurrence of the benthic foraminiferan *Bulimica bortonica* in roughly the same horizon as the fossil itself [14], and that of *Rectuvigerina prisca* ca. 1.8 m below the phosphatised band [15]. In a relatively nearby section located at Hampden, North Otago, the overlap of these two species is extremely short, with *B. bortonica* disappearing at or just before the upper boundary of the local Bortonian stage (38.4 Ma [2]), whereas *R. prisca* first appears during the uppermost Bortonian (ca. 39.5 Ma) [16]. Together, these occurrences constrain the age of *Zygorhiza* sp. to ca. 39.5–38.4 Ma (late Bartonian).

The Waihao Forks specimens represent the oldest known material referred to *Zygorhiza*. The youngest specimens referred to this genus are known from latest Priabonian deposits, such as the Pachuta Marl Member of the Yazoo Formation [17], correlating with the *Turborotalia cerroazulensis* foraminiferal interval zone [18]/Zone P17 [19], dated to 34.6–34.0 Ma [20].

(2) Ingroup

Aetiocetus cotylalveus

Specimens: USNM 25210 (holotype) [21]; USNM 256593 [22].

Localities and horizons: USNM 25210, near mouth of Deer Creek, Lincoln County, Oregon, USA (UO Locality 2503): near top of Yaquina Formation [21]; USNM 256593, Newport, Oregon, USA: uppermost portion of Alsea Formation [22, 23].

Stratigraphic range: early Rupelian–early Chattian (31.0–28.0 Ma).

Comments: Originally thought to be Late Oligocene in age [21, 24], later work on Pacific coast chronostratigraphy [25] proposed a mostly Rupelian age for the Yaquina Formation (30.6–28.0 Ma), with only the uppermost section dating to the earliest Late Oligocene (Chattian). The holotype of *A. cotylalveus* was found near the top of the formation [21], and is therefore likely early Chattian, or possibly latest Rupelian in age (minimum 28.0 Ma [22, 25]). The referred specimen, USNM 256593, was originally reported to have come from the Toledo Formation [22, 23]. The Oligocene part of this formation has been re-designated as Alsea Formation [26], and dated to the early-mid Rupelian, spanning latest Chron C13r to the middle of Chron C12n (ca. 33.7–30.6 Ma [25, 27]). USNM 256593 was derived from the uppermost portion of the Alsea Formation [D. Bohaska 2013, pers. comm.], close to its contact with the overlying Yaquina Formation, which implies an age closer to Chron C12n (31.0–30.6 Ma). This is consistent with the early Chattian holotype, and gives a total age estimate of 31.0–28.0 Ma. Later referrals of a number of vertebrae and ribs from the Late Oligocene ‘Butte Creek beds’ (Scott Mills Formation) [28, 29] may extend the stratigraphic range of *A. cotylalveus* to the late Chattian [30-32], but in the absence of skull material or ear bones cannot currently not be confirmed.

Aetiocetus polydentatus

Specimen: AMP 12 (holotype) [24].

Locality and horizon: South fork of the Morawan River, Morawan, Ashoro, Hokkaido, Japan: upper part of the Upper Tuffaceous Siltstone (UTS) Member of the Morawan Formation [24].

Stratigraphic range: early-late Chattian (26.1–23.3 Ma).

Comments: Biostratigraphy indicates a mid-Rupelian age for the lowermost (Lower Hard Shale) member of the Morawan Formation [33, 34], as well as an earliest Chattian age for the bottom of the overlying Kiroro Formation [35, 36]. Together, these estimates constrain the age of *A. polydentatus* to the latest Rupelian or earliest Chattian. However, this is difficult to reconcile with markedly younger radiometric estimates, which suggest an age of 23.3 ± 0.7 Ma for the bottom of the Kiroro Formation, 28.6 ± 0.7 Ma for the Honbetsuzawa Formation underlying the Morawan Formation, and 26.1 ± 0.7 Ma for the upper part of the Middle Hard Shale Member of the Morawan Formation, which underlies the UTS [37, 38].

Until further data are available to resolve this issue, the age of *A. polydentatus* is therefore here assumed to be 26.1–23.3 Ma (early–late Chattian).

Aetiocetus weltoni

Specimen: UCMP 122900 (holotype) [24].

Locality and horizon: Near mouth of Deer Creek, Lincoln County, Oregon, USA (UCMP locality V79013): uppermost part of the Yaquina Formation, Oregon, USA [22, 24].

Stratigraphic range: early Chattian (28.1–28.0 Ma).

Comments: UCMP 122900 was found on the same beach as the holotype of *A. cotylalveus*, and may either have originated from the same stratigraphic horizon (about 28 Ma), or from a slightly younger layer [22, 24]. Although originally thought to date from the latest Oligocene, the uppermost part of the Yaquina formation has now been re-dated to the earliest Chattian (28.1–28.0 Ma [25]; see discussion of *A. cotylalveus*).

Aglaocetus moreni

Specimens: MLP 5-1 (holotype) [39]; MLP 5-14 [40] ; FMNH P13407 [41].

Localities and horizons: MLP 5-1, 5-14, Castillo, Trelew, Chubut, Argentina: lower part of the Gaiman Formation [39, 40]; FMNH P13407, southwest of Pico Salamanca and north of Commodoro Rivadavia, Chubut, Argentina: Patagonian Marine Formation (Gaiman or Monte León formations) [41].

Stratigraphic range: early Burdigalian (19.8–18.2 Ma).

Comments: The holotype of *A. moreni* was found in “a mixture of sand and clay, including a stratum of sand capped by volcanic sand.” ([39]:1). The original description further explained that ”an apparently contemporaneous bed in Santa Cruz, also containing cetacean bones, has yielded oysters of an extinct species (*Ostrea patagonica*), and one skull from Chubut has one of the same oysters attached” [39: 1-2]. MLP 5-14 was later discovered at the same locality, described as “Castillo, frente a Trelew, Chubut” [40: 369], and reported to have come from the Patagonian Marine Formation (“formación patagónica marina”, [40: 364]). There are no data on the lithology of the source horizon of FMNH P13407, although this specimen was reportedly also recovered from the Patagonian Marine Formation [41].

The Patagonian Marine Formation corresponds to the partially correlated Monte León and Gaiman formations, exposed in Santa Cruz (e.g. Garn Bajo de San Juan) and the lower Chubut river valley near Trelew (e.g. at Bryn Gwyn/ Loma Blanca), respectively [42]. The Gaiman Formation consists of layers of marine sandstones and mudstones, as well as tuffs and tuffaceous sandstones. In addition, the lower part also contains occasional thin layers of *Ostrea* and bones of marine vertebrates [43]. Thus, the portion of the Gaiman Formation exposed near Trelew provides a likely match for the unit that yielded *A. moreni* [39, 40, 44].

In the area around Trelew, the lower part of Gaiman Formation overlies the continental Sarmiento Formation, which contains a mammal fauna correlating with the Colhuehuapian South American Land Mammal Age [43, 45]. The latter has been $^{40}Ar/^{39}Ar$ dated to 20.9–19.8 Ma [46], thus providing a maximum age of 19.8 Ma (earliest Burdigalian) for the lower part of the Gaiman Formation in this area. Since virtually all vertebrate material recovered so far originated from the lowermost levels of the Gaiman Formation, close to its contact with the Sarmiento Formation [Cozzuol 2010, pers. comm.], an early Burdigalian age (19.8–18.2 Ma) is here assumed for *A. moreni*.

Aglaocetus patulus

Specimens: USNM 23690 (holotype); USNM 13472, 23049 [47].

Localities and horizons: USNM 23690, near mouth of Pope's Creek, Stratford Bluffs, Westmoreland County, Virginia, USA; USNM 13472, Kenwood Beach cliff, near road end at Governor Run, Calvert County, Maryland, USA; USNM 23049, near mouth of Parker Creek, Calvert County, Maryland, USA; all specimens come from bed 14 of the Calvert Formation [47].

Stratigraphic range: Langhian (14.5–13.9 Ma).

Comments: The base of bed 14 has been dated to ca. 13.1 Ma (Serravallian) based on strontium dating [48], but was recently reassigned to the late Langhian (ca. 14.5 Ma) based on palaeoclimatic considerations, such as the bed predating the expansion of the East-Antarctic ice sheet around 13.9 Ma [49].

Balaena ricei

Specimen: USNM 22553 (holotype) [50].

Locality and horizon: Rice's Pit, Hampton, Virgina, USA: ?Morgarts Beach Member (see comments below) of the Yorktown Formation [50].

Stratigraphic range: Zanclean (4.9–4.4 Ma).

Comments: The layer of the Yorktown Formation that yielded the specimen reportedly falls into planktonic foraminiferal zone N19 [51] and the *Orionina vaughani* ostracod assemblage zone [52, 53]. Whereas zone N19 correlates with the latest Messinian–early Zanclean [54], the *Orionina vaughani* zone is seemingly restricted to the Zanclean (starting at 5.3 Ma), and correlates with the uppermost part of nannofossil zone NN12 and zones NN13–15 on the one hand, and foraminiferal zones N19–20 [53: fig. 4] on the other. K-Ar dating of a sample typical of the *Orionina vaughani* zone indicates an age of 4.4 ± 0.2 Ma [53], roughly coincident with the top of zone N19 [54]. Strontium dating suggests an age of 4.9 Ma for the base of the Yorktown 4.9 Ma [55]. Taken together, these observations indicate an age bracket of 4.9–4.4 Ma for *B. ricei*. Note that, while the exact position of the horizon that

yielded *B. ricei* within the Yorktown Formation is unknown, Westgate and Whitmore speculated that the specimen was recovered from the Morgarts Beach Member [50]. The latter has been strontium dated to 3.0–2.8 Ma [55], thus seemingly precluding its correlation with zone N19 – although the top of N19 still falls within the range of uncertainty of the Sr dates. The type specimen might therefore have been derived from the somewhat older Rushmere Member of the Yorktown Formation, which has been strontium dated to 4.9–4.6 Ma [55] and is also exposed in Rice's pit.

Balaenella brachyrhynus

Specimen: NMB 42001 (holotype) [56].

Locality and horizon: First Channel Dock, Kallo, Antwerp, Belgium: Kattendijk Formation [56].

Stratigraphic range: Zanclean (5.0–4.4 Ma).

Comments: The Kattendijk Formation has been dated to 5.0–4.4 Ma based on its dinoflagellate assemblage [57].

"Balaenoptera" portisi

Specimens: MRSN PU13808 (holotype) [58]; UCMP 219135 [5]; MCZ 17882 (holotype of *Balaenoptera floridana* Kellogg, 1944); SDNHM 21507, 65769, 68698 [59].

Localities and horizons: MRSN PU13808, Montafia, Piedmont, Italy: Sabbie d'Asti Formation [58]; UCMP 219135, San Gregorio, California, USA (UCMP locality V99840): Purisima Formation [5]; MCZ 17882, American Chemical Company phosphate pit, Pierce, Polk County, Florida, USA: Bone Valley Formation (Palmetto Fauna [60]) [59]; SDNHM 21507, SDNHM locality 3068, San Diego, San Diego County, California, USA; SDNHM 65769, SDNHM locality 3782, Chula Vista, San Diego County, California, USA; SDNHM 68698, SDNHM locality 4198, Chula Vista, San Diego County, California, USA; all SDNHM specimens are from the San Diego Formation [59].

Stratigraphic range: Zanclean-Piacenzian (5.3–3.0 Ma).

Comments: The molluscan assemblage occurring within the Sabbie d'Asti Formation falls within Mediterranean Pliocene Molluscan Unit 1 (5.3–3.0 Ma) [61-64], whereas its foraminiferal assemblage correlates with biozone MPl4a (4.0–3.6 Ma) [65]. Taken together, these estimates indicate an age range of 4.0–3.0 Ma for the holotype. By contrast, the Palmetto Fauna has been correlated with the latest Hemphillian (5.8–4.9 Ma [54]), and likely the earliest Pliocene (starting at 5.3 Ma) based on sea-level data [60]. Similarly, the portion of the Purisima Formation that yielded UCMP 219135 is likely to be Zanclean or, at least, earliest Piacenzian in age (minimum of 3.4–3.3 Ma) [5], thus indicating a combined age range of 5.3–3.0 Ma. The horizons within the San Diego Formation which yielded the

remaining referred specimens are currently unknown, but could potentially extend the range of this taxon to the Late Pliocene or earliest Pleistocene [66].

"Balaenoptera" ryani

Specimen: CAS 1733 [67].

Locality and horizon: Ca. 5 miles east of Monterey, California, USA: upper part (Canyon del Rey Diatomite Member) of the Monterey Formation [67, 68].

Stratigraphic range: early Tortonian (11.6–9.5 Ma).

Comments: The original description states that the "top stratum" of the section in which the specimen was found "is loose and scarcely consolidated, characters which accompany the upper part of the Monterey shale member whenever definitely exposed" [67: 238]. Barron [68] placed a rock sample (CAS 866) from the type locality, collected at the same time as the CAS 1733, into the Canyon del Rey Diatomite Member. Fission-track dating of associated ash layers place the base of this unit at 11.3 ± 0.9 Ma [69]. A second ash horizon from within the Canyon del Rey Diatomite Member has been dated to 8.4 ± 0.8 Ma, but it is unclear whether the holotype was retrieved from above or below this horizon The rock sample (CAS 866) apparently associated with the holotype has yielded the diatom *Denticulopsis lauta* [68], which last appears around 9–10 Ma [70, 71]. Together, these observations imply an early Tortonian (11.6–9.5 Ma) age for *B. ryani*.

Balaenoptera siberi

Specimens: SMNK no number (holotype) [72]; SMNS 47307 [73].

Localities and horizons: Agua de Lomas, Peru: Pisco Formation [72, 73].

Stratigraphic range: late Tortonian (8.0–7.0 Ma).

Comments: The Pisco Formation at Agua de Lomas has been dated to 8.0–7.0 Ma based on K-Ar dating [74, 75].

Balaenula astensis

Specimens: MSNTUP I12555 [4, 76].

Locality and horizon: Portacomaro d'Asti, Piedmont, Italy: Sabbie d'Asti Formation [4].

Stratigraphic range: Zanclean-Piacenzian (4.0–3.0 Ma).

Comments: The molluscan assemblage occurring within the Sabbie d'Asti Formation falls within Mediterranean Pliocene Molluscan Unit 1 (5.3–3.0 Ma) [61-64], whereas its foraminiferal assemblage correlates with biozone MPl4a (4.0–3.6 Ma) [65, 77]. Taken together, these estimates indicate an age range of 4.0–3.0 Ma.

***Balaenula* sp.**

Specimen: SMAC 1309 [78, 79].

Locality and horizon: Fukagawa, Hokkaido, Japan: Chippubetsu (Chichibubetsu) Formation [79].

Stratigraphic range: Messinian–Zanclean (6.4–4.1 Ma).

Comments: The Chippubetsu Formation, which forms part of the Fukagawa Group exposed in Hokkaido, Japan [80]. The specimen was found in a horizon below a tuff level (“T2”) likely correlative with the S1 tuff of the Takikawa Formation [81], which has been dated to 4.1 ± 0.6 Ma based on fission-track dating [82]. The age of the base of the Chippubetsu Formation is currently unknown. However, the lower part of this formation has been correlated with the upper part of the Horokaoshirarika Formation based on palynological data [80], with the latter falling into the *Neodenticula kamtschatica* diatom zone [83], dated to ca. 6.4–3.5 Ma [84]. Together, these estimates imply an approximate age range of 6.4–4.1 Ma.

Brandtocetus chongulek

Specimens: TNU skulls 2, 4, A

Locality and horizon: Southern coast of Tobechik Lake, Kerch Peninsula, Crimea: Chersonian Formation [85].

Stratigraphic range: early Tortonian (11.2–9.6 Ma).

Comments: The Chersonian Formation has been dated to the late Sarmatian sensu lato, based on the occurrence of numerous *Mactra* (*Chersonimactra*) *caspia* [85]. In the region of the Central Paratethys, the Sarmatian Stage (sensu stricto) as a whole has been assigned the Middle Miocene [86, 87]. By contrast, the upper part of the Sarmatian as found in the area of the eastern Paratethys correlates with the early Tortonian (11.2–9.6 Ma) [88, 89].

Cephalotropis coronatus

Specimens: USNM 9352 (holotype) [90]; USNM 489194 [7]; *C.* cf. *coronatus*, MMG A3 (holotype of *Cephalotropis nectus*) [91, 92].

Localities and horizons: USNM 9352, Chesapeake Bay, USA: likely St Mary’s Formation [90, 93]; USNM 489194, Calvert County, Maryland: Little Cove Point Member of the St Mary’s Formation [23]; MMG A3, Adiça, Lower Tagus Basin, Portugal: Cotter’s lithostratigraphic zone VIIb [91, 92].

Stratigraphic range: early–late Tortonian (11.7–8.5 Ma).

Comments: USNM 9352 lacks detailed stratigraphic information, although Cope believed that it may have been derived from the “Yorktown Formation” [90: 145] – later corrected to St Mary Formation by Kellogg [93]. Strontium dating indicates the Little Cove Point Member of the St Mary’s Formation to be almost exclusively early Tortonian (11.7–10.0 Ma) [48, 49].

Note that de Verteuil and Norris [94] estimated the relevant portion of the St Mary's Formation to be younger (ca. 10.4–8.8 Ma). However, their assignment of these strata to dinoflagellate zone DN8 for the most part does not preclude the older strontium dates [94: fig. 3]. Cotter's lithostratigraphic zone VIIb [95] includes the youngest Miocene rocks present in the Lower Tagus Basin. At Adiça, the exposed rock seems to date to the middle or late Tortonian (9.5–8.5 Ma) [96, 97]. Together, this indicates a total age range of 11.7–8.5 Ma.

"Cetotherium" megalophysum

Specimens: USNM 10593 (holotype) [98]; USNM 22962, 205510, 241531 [7].
Localities and horizons: USNM 10593, Chesapeake Bay, USA: formation not stated [98]; USNM 22962, Charles County, Maryland, USA: formation not stated; USNM 205510, Saint Mary's County, Maryland, USA: St Mary's Formation; USNM 241531, Chesapeake Bay, USA: St Mary's Formation.
Stratigraphic range: early Tortonian (11.7–10.0 Ma).
Comments: USNM 10593 lacks detailed stratigraphic information [98]. However, a number of undescribed, preliminarily identified specimens from the Little Cove Point Member of the St. Mary's Formation are likely referable to *C. megalophysum* [23], and indicate an almost exclusively early Tortonian (11.7–10.0 Ma) age for this taxon [48, 49]. See *Cephalotropis coronatus* for further details.

Cetotherium rathkii

Specimen: PIN 1840/1 (holotype) [99].
Locality and horizon: Zhelezny Rog Cape, Taman Peninsula, Russia: formation unknown, late Sarmatian sensu lato [99, 100].
Stratigraphic range: early Tortonian (11.2–9.6 Ma).
Comments: In the region of the Central Paratethys, the Sarmatian Stage (sensu stricto) as a whole has been assigned the Middle Miocene [86, 87]. By contrast, the upper part of the Sarmatian as found in the area of the eastern Paratethys correlates with the early Tortonian (11.2–9.6 Ma) [88, 89]. The age of the late Sarmatian deposits on the Taman Peninsula are still a matter of debate. Fission-track dating places their lower boundary at ca. 11.2 ± 0.7 Ma, while chronostratigraphy suggest an upper limit of roughly 9.6 Ma (or possibly somewhat younger, based on an estimate of 9.5 ± 0.9 Ma for the upper Sarmatian deposits of the nearby Kerch Peninsula) [88]. By contrast, biostratigraphic data suggest an age range of 8.9 Ma to 7.9–7.7 Ma [89]. Until further data are available to resolve this conflict, we assume the age of *C. rathkii* to be 11.2–9.6 Ma, in line with the radiometric evidence.

Cetotherium riabinini

Specimens: NMNH-P 668/1 (holotype) [100].

Locality and horizon: Outskirts of Nikolaev, Ukraine: possibly Chersonian Formation [100].

Stratigraphic range: early Tortonian (11.2–9.6 Ma).

Comments: See discussion of *Brandtocetus chongulek*.

ChM PV4745

Locality and horizon: Dorchester County, South Carolina, USA: Ashley Formation [101, 102].

Stratigraphic range: late Rupelian (29.6–28.1 Ma).

Comments: The Ashley Formation falls within calcareous nannoplankton zone NP24 [103], ranging from 29.6–26.8 Ma [12, 20]. The presence of the dinoflagellate *Chiropteridium lobospinosum* indicates a minimum age of 28.1 Ma [103, 104]. Together, these constraints imply a late Rupelian age (29.6–28.1 Ma) for the Ashley Formation, which is further supported by a strontium date of 29.1 Ma [105].

Chonecetus goedertorum

Specimens: LACM 131146 (holotype); LACM 138027 [24].

Locality and horizon: Twin River Quarry, Clallam County, Washington, USA (LACM locality 5930): Pysht Formation [24].

Stratigraphic range: early Chattian (28.1–26.5 Ma).

Comments: Samples of the Pysht Formation from either side of Twin Rivers correlate with Chrons C9r–C8r (28.1–26.5 Ma) [106].

Chonecetus sookensis

Specimen: CMN FV64443 [107].

Locality and horizon: Between the mouths of Bonilla Creek and Carmanah Creek, southwest coast of Vancouver Island, British Columbia, Canada: Sooke Formation [107].

Stratigraphic range: late Chattian (24.8–24.1 Ma)

Comments: Based on magnetostratigraphic evidence and associated mollusc and mammal data, the Sooke Formation has been correlated with Chron C6Cr (24.8–24.1 Ma) [108].

Diorocetus chichibuensis

Specimens: SMNH VeF68 (holotype); SMNH VeF19 [109].

Locality and horizon: Chichibu, Saitama Prefecture, Japan: Nagura Formation [109].

Stratigraphic range: late Burdigalian-Langhian (16.4–15.1 Ma).

Comments: The Nagura Formation forms the lowermost part of the Chichibumachi Group, which correlates with foraminiferal zone N8 [51, 110, 111] and thus spans the Burdigalian-Langhian boundary (16.4–15.1 Ma) [20, 54].

Diorocetus hiatus

Specimens: USNM 16783 (holotype); USNM 16567, 16871, 23494 [112].

Localities and horizons: USNM 16567, 16783, near mouth of Parker Creek, Calvert County, Maryland, USA: bed 14 of the Calvert Formation; USNM 16871, 23494, near road end at Governor Run, Calvert County, Maryland: Calvert Formation (USNM 23494 is from bed 14) [112].

Stratigraphic range: Langhian (14.5–13.9 Ma).

Comments: The base of bed 14 of the Calvert Formation has been dated to ca. 13.1 Ma (Serravallian) based on strontium dating [48], but was recently reassigned to the late Langhian (ca. 14.5 Ma) based on palaeoclimatic considerations, such as the bed predating the expansion of the East-Antarctic ice sheet around 13.9 Ma [49].

Diorocetus shobarensis

Specimens: HMN F00005–00009 and further associated postcranial material (holotype; all specimens are part of the same individual); HMN F00063 [113].

Localities and horizons: HMN F00005–00009 Monde-cho; HMN F00063, Nishihonmachi; both localities are situated along the Saijyo River, Shobara City, Hiroshima Prefecture, Japan: Korematsu Formation [113].

Stratigraphic range: late Burdigalian–Langhian (17.0–14.9 Ma)

Comments: The Korematsu Formation in the Shobara area underlies the Itabashi Formation, and together with latter forms the middle/upper portion of the Bihoku Group [114]. Calcareous nannoplankton correlates the Korematsu Formation and the lower part of the Itabashi Formation with the upper part of nannoplankton zone NN4 [114], suggesting a latest Burdigalian or early Langhian age with a minimum of 14.9 Ma [20, 54]. A further study [115] specifically correlated the Korematsu and Itabashi formations exposed along the stretch of the Saijyo River that yielded the whale fossils with the top of nannoplankton zone NN4, and the beginning of the "Mid-Neogene Climatic Optimum" (ca. 16–15 Ma [49, 116]). By contrast, diatom-based biostratigraphic data place the upper part of the Bihoku Group almost entirely in the late Burdigalian (17.0–16.0 Ma) [35, 36, 117, 118]. Together, these estimates amount to an age range of 17.0–14.9 Ma.

Eomysticetus whitmorei

Specimen: ChM PV4253 (holotype) [119].

Locality and horizon: Chandler Bridge Creek, Dorchester County, South Carolina, USA: uppermost portion (bed 3) of the Chandler Bridge Formation [119, 120].

Stratigraphic range: early Chattian (28.1–26.8 Ma).

Comments: There are no published calcareous nannoplankton or foraminiferal age estimates for the Chandler Bridge Formation, but the unit has been speculated to correlate with nannoplankton zone NP24 [119, 121], ranging from 29.6–26.8 Ma [12, 20]. Dinoflagellates suggest a correlation of the lower portion of the Chandler Bridge Formation (bed 1) with dinocyst zone D14 [121], variously defined to terminate around 30 Ma (middle Rupelian) [12] around the Rupelian/Chattian boundary (ca. 28 Ma; [104]). In particular, the occurrence of *Chiropteridium lobospinosum* in bed 1 [121] suggests a minimum age of ca. 28.1 Ma [104]. No dinoflagellates have been reported from the uppermost portion of the formation that yielded the holotype of *E. whitmorei*. However, given (i) the latest Rupelian age of the underlying Ashley Formation [105] and, possibly, bed 1, (ii) the relatively high stratigraphic position of type horizon (bed 3) and (ii) the latest Oligocene age of the overlying Edisto Formation [105], we here assume an early Chattian age for *E. whitmorei* (28.1–26.8 Ma).

Eschrichtioides gastaldii
Specimen: MRSN PU13802 (holotype) [62].
Locality and horizon: Cortandone, Piedmont, Italy: Sabbie d'Asti Formation [62].
Stratigraphic range: Zanclean–Piacenzian (4.0–3.0 Ma)
Comments: See *Balaenula astensis* for details.

Eubalaena belgica
Specimen: IRSNB M879 (holotype) [122].
Locality and horizon: Antwerp, Belgium: Kruisschans Sand Member of the Lillo Formation [122].
Stratigraphic range: Piacenzian (3.2–2.8 Ma).
Comments: The Kruisschans Sand Member has been dated to 3.2–2.8 Ma based on dinoflagellate cyst and sequence stratigraphy [57].

Eubalaena shinshuensis
Specimen: SFM CV0024 (holotype) [78].
Locality and horizon: Shinshushinmachi, Nagano Prefecture, Japan: lowermost part of the Gonda Formation [78].
Stratigraphic range: Messinian (6.1–5.9 Ma).
Comments: The Gonda Formation is frequently referred to as the lowermost member of the Shigarami Formation [123]. A K-Ar date of 6.1 Ma for the Koso Tuff, which forms part of the underlying Ogawa Formation, and 5.9 Ma for a lava deposit forming part of the upper Shigarami Formation [124] constrain the age of *E. shinshuensis* to the Messinian.

Gricetoides aurorae

Specimens: USNM 182921 (holotype); USNM 25762, 183004, 244468 [125].

Locality and horizon: Lee Creek Mine, Aurora, North Carolina, USA: lower part of the Yorktown Formation [125].

Stratigraphic range: Zanclean (4.9–3.9 Ma).

Comments: Strontium dating places the base of the Yorktown Formation at 4.9 Ma [55]. The top of the Yorktown Formation at Lee Creek Mine is no younger than the middle of the *Orionina vaughani* ostracod assemblage zone, originally dated to ca. 3.7 Ma [53]. This is roughly in agreement with the top of nannoplankton zone NN14 (3.9 Ma) [20, 54], the second of the three nannoplankton zones correlating with the *Orionina vaughani* zone [53]. Taken together, these observations indicate an age bracket of 4.9–3.9 Ma.

Herpetocetus bramblei

Specimens: UCMP 82465 (holotype) [126]; UCMP 219079 [5], 219111, 219112 [127].

Localities and horizons: UCMP 82465, Opal Cliffs, Santa Cruz, California, USA (UCMP locality V6875): Crab Marker Horizon [128], lower portion of the Purisima Formation [5, 126]; UCMP 219079, San Gregorio, California, USA (UCMP locality V99854): lowermost portion of the Purisima Formation [5]; UCMP 219111, 219112, California, USA: unspecified horizons of the Purisima Formation [129].

Stratigraphic range: Messinian–Zanclean (6.4–4.9 Ma).

Comments: Powell et al. [130] chemically correlated an ash bed from the basal portion of the Purisima formation below the Crab Marker Bed with similar deposits elsewhere in California, which have been dated to between 5.6–5.0 and 4.7 Ma. However, this estimate is contradicted by the occurrence of the diatom *Thalassiosira miocenica*, as well as the apparent absence of *T. oestrupii,* in the Opal Cliff section, implying its assignment to the upper part of subzone b of the *Nitzschia reinholdii* zone (6.4-5.5 Ma) [128, 130]. Given the absence of direct dating evidence for the ash layer in the basal Purisima Formation, w here assume a lower age boundary of 6.4 Ma for *H. bramblei*. This is further supported by the occurrence of UCMP 219079 in a similarly aged horizon (based on diatom data) near the bottom of the San Gregorio section north of Santa Cruz [5]. Magnetostratigraphic correlations recognise a depositional hiatus above the Opal Cliff section, lasting from 4.9–3.7 Ma [128, 130]. Taken together, these observations imply an age range of 6.4–4.9 Ma.

Herpetocetus transatlanticus

Specimens: USNM 182962 (holotype); USNM 183074, 183075, 183077, 299652–299656, 312542–312544 [126].

Locality and horizon: Lee Creek Mine, Aurora, North Carolina, USA: Yorktown Formation (USNM 182962 may have been derived from the lowermost 3 metres of the formation) [126].
Stratigraphic range: Zanclean (4.9–4.4 Ma).
Comments: In the Lee Creek Mine, the *Orionina vaughani* ostracod assemblage zone (dated to at least 4.4 Ma based on K-Ar dating [53]) starts about 3.5 metres above the base of the Yorktown [52]. Strontium dating indicates an age of 4.9 Ma for the base of the Yorktown Formation [55], thus indicating an age bracket of 4.9–4.4 Ma for the type specimen.

Isanacetus laticephalus
Specimens: MFM 28501 (holotype); MFM 18004 [131].
Localities and horizons: MFM 28501, Ayama District, Mie Prefecture, Japan: Hiramatsu Member of the Awa Group; MFM 18004, Mizunami City, Gifu Prefecture, Japan: Yamanouchi Member of the Akeyo Formation [131].
Stratigraphic range: late Burdigalian (17.5–16.0 Ma).
Comments: Planktonic foraminiferal data place the Hiramatsu Member of the Awa Group in zone N7 [132, 133], ranging from 17.5–16.4 Ma [20, 54]; and the Yamanouchi Member of the Ayeko Formation in the upper part of zone N7 and the lower part of zone N8 [134], with zone N8 ranging from 16.4–15.1 Ma [20, 54]. Fission-track dating of a sample from the base of the Yamanouchi Member indicates an age of 17.3 ± 1.4 Ma [135]. Although the termination of zone N8 at 15.1 Ma could imply a partially Langhian range, we here constrain the age of *I. laticephalus* to the late Burdigalian (17.5–16.0 Ma) based on (i) the confirmed Burdigalian age of MFM 28501; (ii) the fact that the lower boundary of the Yamanouchi Member predates the Burdigalian/Langhian boundary by as much as 1 Ma, or possibly more; and (iii) the fact that the Yamanouchi Member was only correlated with the *lower* part of zone N8.

Janjucetus hunderi
Specimens: NMV P216929 (holotype) [136]; NMV P229455/ USNM 534009 (cast of a specimen held in a private collection) [137].
Locality and horizon: Jan Juc Beach, near Torquay, Victoria, Australia: Jan Juc Marl [136, 137].
Stratigraphic range: early? Chattian (28.1–25.6 Ma; **preliminary assessment**)
Comments: The Jan Juc Marl is placed mostly into the Chattian by both biostratigraphy and radiometric dating, with a minimum age of 23.9 (based on the overlying Puebla Formation) and a maximum age exceeding 27.2 Ma [138]. However, the exact position of the known material within the Jan Juc Marl is unknown. Several recent cladistic analyses, including the present study, have proposed a close relationship of *Janjucetus* and *Mammalodon* [102,

139]. Since the two taxa must have separated prior to the first occurrence of *Mammalodon* and its relatives during the early Chattian (see discussion of OU 22026 for further details), we here assume an early Chattian (28.1–25.6 Ma) age for *Janjucetus*, pending the discovery of temporally better constrained specimens.

Joumocetus shimizui

Specimen: GMNH PV2401 (holotype) [140].

Locality and horizon: Kabura River, Yoshii, Takasaki, Gunma Prefecture, Japan: Haraichi Formation [140].

Stratigraphic range: early Tortonian (11.3–11.0 Ma).

Comments: The Haraichi Formation is bracketed by the underlying Baba and the overlying Kamikoizawa tuffs [140]. $^{40}Ar/^{39}Ar$ dating places the Baba Tuff at 11.3 Ma [141]. The Kamikoizawa Tuff has not yet been dated directly, but an earliest late Tortonian age (less than 11.0 Ma) has been suggested on the basis of sedimentation rates [142].

Kurdalagonus mchedlidzei

Specimen: NMRA 10476 [143].

Locality and horizon: Belaya River, Maikop, Adygea, Russia: lower part of the Blinovo Formation [143].

Stratigraphic range: Serravallian–early Tortonian (12.1–11.2 Ma).

Comments: Mollusc biostratigraphy indicates the lower part of the Blinovo Formation to fall into the Middle Sarmatian [144], which in the region of the Eastern Paratethys spans the Serravallian/early Tortonian boundary, and ranges from ca. 12.1–11.2 Ma [88].

Llanocetus denticrenatus

Specimen: USNM 183022 [145, 146].

Locality and horizon: Seymour Island, Antarctica: top of Telm 7, La Meseta Formation [145-147].

Stratigraphic range: late Priabonian (34.2–34.0 Ma).

Comments: Strontium dating indicates Telm 7 to have been deposited between 39.1 and 34.0 Ma [148]. Samples from near the top of the formation fall closest to the presumed fossil horizon [145, 147], thus indicating an age of 34.2–34.0 Ma [148, 149].

Mammalodon colliveri

Specimens: NMV P199986 (including NMV P17535) (holotype); *Mammalodon* cf. *colliveri*, NMV P173220, P199587 [102].

Localities and horizons: NMV P199986, Jan Juc Beach, near Torquay, Victoria, Australia: upper 5 m of the Jan Juc Marl [102]; NMV P199587, Bells Beach, near Torquay, Victoria,

Australia: Point Addis Limestone [136]; NMV P173220, locality and horizon have not been stated [102].

Stratigraphic range: late Chattian (25.7–23.9 Ma).

Comments: Biostratigraphic evidence and foraminiferal $^{87}Sr/^{86}Sr$ isotope ratios [150-152], indicate and age of 25.7–23.9 Ma for the holotype [102]. The base of the Point Addis Limestone has been dated to 24.2 Ma (+1.3, -1.2 Ma) [138], whereas the age of its top is the same as for the Jan Juc Marl and constrained by the contact with the overlying Puebla Formation (23.9 Ma) [151].

Mauicetus parki

Specimens: OU 11573 (holotype) [153, 154]; OU 22545 [153].

Localities and horizons: OU 11573, possibly Milburn Quarry, South Otago, New Zealand: Milburn Limestone [153, 154]; OU 22545, Hakataramea Valley, South Canterbury, New Zealand: Otekaike Limestone [153].

Stratigraphic range: late Chattian (25.2–23.0 Ma).

Comments: The precise stratigraphic context of OU 11573 is currently unknown, but likely falls within the early Waitakian (Oligocene, 25.2–23.0 Ma [2]). This estimate is supported by s strontium date of 23.8 ± 0.7 Ma for the horizon that yielded OU 22545.

"Megaptera" hubachi

Specimen: MB Ma28570 (holotype) [155].

Locality and horizon: Near Coquimbo, Chile: formation not stated, but likely to be Coquimbo Formation (see comments) [155].

Stratigraphic range: Zanclean (5.3–3.6 Ma).

Comments: In his original description, Dathe [155] did not specify the formation that has yielded the specimen, but stated that the fossil had been found at the western end of the "Bahia de Guayacan", southwest of Coquimbo. He furthermore speculated that the fossil had come from a sandstone of Early Pliocene age, although he could not exclude the possibility of a Middle Miocene age. Dathe's figure of the type locality [155: fig. 10] shows primarily the Bay of Tongoy, which is dominated by exposures of the Miocene–Pliocene Coquimbo Formation [156, 157]. The latter is divided into 16 lithostratigraphic units, 5 of which (units 3, 4 and 11–13) have yielded cetaceans remains [156, 158]. Of the latter, only units 4 and 11–13 contain sandstone, with only unit 11 being dominated by this lithology. Strontium dating and biostratigraphic evidence place units 3 and 4 in the Tortonian (11.9–11.2 Ma), and thus make them much older than the Early Pliocene age suggested by Dathe [155]. By contrast, units 11–13 have been dated to the earliest Pliocene based on biostratigraphic grounds, as well as Sr dates of 5.2 ± 0.7 Ma for the base of unit 12, 4.9 ± 0.7 Ma 2 m above the base of

unit 12, and 2.2 ± 0.5 Ma 6 m below the top of unit 14 (inferred mean ages for units 12 and 13 are 5.0 and 4.3 Ma, respectively) [156]. Considering the coincidence of (i) the occurrence of numerous cetacean fossils in units of the correct lithology and age; and (ii) the broad exposure of these units in the type area of "*M. hubachi*", we suggest that the holotype of this species was recovered from the upper, Early Pliocene portion of the Coquimbo Formation. As a result, we follow Dathe [155] in assigning an Early Pliocene (Zanclean, 5.3–3.6 Ma) age to the holotype. Note, however, that Deméré *et al.* [59] suggested a Piacenzian (3.6–2.6 Ma) age for this specimen.

"Megaptera" miocaena

Specimen: USNM 10300 (holotype) [159].

Locality and horizon: Lompoc, California: Monterey Formation [159].

Stratigraphic range: late Tortonian (7.6–7.3 Ma).

Comments: According to the original description, the skull was found by workers of the Celite Products Company quarrying diatomaceous earth, "about 140 feet below the exposed surface of the bed" ([159: 3]). Recent studies focusing on the diatomaceous deposits quarried near Lompoc reported that roughly 218 metres of diatomite, belonging to the upper siliceous member of the Monterey Formation, are exposed at the Celite diatomite quarry [160]. These deposits range from 8.2–7.3 Ma, with the latter date denoting the upper limit of the economically mineable deposits [160]. A similar date is suggested by the diatom assemblage contained in the matrix adhering to the holotype specimen (*Rouxia californica* Zone/ NPD7A; 7.6–6.6 Ma [161, 162]) [23; N. Kohno 2015, pers. comm.]. Together, these data suggest a latest Tortonian age (7.6–7.3 Ma) for *M. miocaena*. Note that additional specimens possibly referable to "*M.*" *miocaena* have also been reported from the Late Miocene–Pliocene Purisima, San Mateo and San Diego formations (California, USA). Pending further study, these specimens may extend the range of the lineage by up to 5 Ma.

Metopocetus durinasus

Specimen: USNM 8518 (holotype) [90, 163].

Locality and horizon: Near the mouth of the Potomac River: the formation has not been specified, but may be the St Mary's Formation (see comments) [90]

Stratigraphic range: early Tortonian (11.7–10.0 Ma).

Comments: USNM 8518 was collected from "a Miocene marl from near the mouth of the Potomac river" [90: 143]. Subsequent authors interpreted this description of the type locality to refer to either the Calvert Formation [93, 163] or the St. Mary's Formation [164], implying either a Langhian or a Serravallian age, respectively. To date, no other cetotheriid remains from the Calvert Formation have been described, but at least two cetotheriids (*Cephalotropis*

coronatus and "*Cetotherium*" *megalophysum*; see above) occur in the Little Cove Point Member of the St Mary's Formation. This, together with the fact that the suggestion of the St Mary's formation as a potential source unit was published just eight years after the original description of the species, might support the origin of *M. durinasus* from the Little Cove Point Member. The latter has been strontium dated to the early Tortonian (11.7–10.0 Ma) [48, 49], which is the age assumed here pending the emergence of better-constrained occurrence data.

Micromysticetus rothauseni

Specimen: ChM PV4844 (holotype) [165].

Locality and horizon: Chandler Bridge Creek, Dorchester County, South Carolina, USA: near top of the Ashley Formation [165].

Stratigraphic range: late Rupelian (29.6–28.1 Ma)

Comments: See ChM PV4745 for details.

Miocaperea pulchra

Specimen: SMNS 46978 (holotype) [166].

Locality and horizon: Aguada de Lomas, Peru: Pisco Formation [166].

Stratigraphic range: late Tortonian (7.5–7.3 Ma).

Comments: The Pisco Formation exposed at Aguada de Lomas, Peru [166], which has been dated to 7.5–7.3 Ma based on K-Ar and $^{87}Sr/^{86}Sr$ dating [74, 75, 167].

Morawanocetus yabukii

Specimens: AMP 1 (holotype) [24]; AMP 14 [168].

Locality and horizon: Morawan, Ashoro, Hokkaido, Japan: Upper Tuffaceous Siltstone Member of the Morawan Formation [24].

Stratigraphic range: early–late Chattian (26.1–23.3 Ma).

Comments: See *Aetiocetus polydentatus* for details.

Morenocetus parvus

Specimens: MLP 5-11 (holotype); MLP 5-15 [40].

Locality and horizon: Castillo, Trelew, Chubut, Argentina: lower part of the Gaiman Formation [40].

Stratigraphic range: early Burdigalian (19.8–18.2 Ma).

Comments: Both MLP 5-11 and 5-11 were originally reported to have come from the Patagonian Marine Formation [40]. The latter corresponds to the partially correlated Monte León and Gaiman formations, exposed in Santa Cruz (e.g. Garn Bajo de San Juan) and the lower Chubut river valley near Trelew (e.g. at Bryn Gwyn/ Loma Blanca), respectively [42]. In

the area around Trelew, the lower part of Gaiman Formation overlies the continental Sarmiento Formation, which contains a mammal fauna correlating with the Colhuehuapian South American Land Mammal Age [43, 45]. The latter has been $^{40}Ar/^{39}Ar$ dated to 20.9–19.8 Ma [46], thus providing a maximum age of 19.8 Ma (earliest Burdigalian) for the lower part of the Gaiman Formation in this area. Since virtually all vertebrate material recovered so far originated from the lowermost levels of the Gaiman Formation, close to its contact with the Sarmiento Formation [Cozzuol 2010, pers. comm.], an early Burdigalian age (19.8–18.2 Ma) is here assumed for *M. parvus*.

Nannocetus eremus

Specimens: UCMP 26502 (holotype) [169]; UCMP 108558 [126].

Locality and horizon: UCMP 26502, south of Humphreys, San Gabriel Mountains, Los Angeles County, California, USA (UCMP locality V2202): basal portion of the Towsley Formation; UCMP 108558, De Laveaga Park, Santa Cruz, Santa Cruz County, California, USA (UCMP locality V6200): upper portion of the Santa Margarita Formation [126, 170, 171].

Stratigraphic range: late Tortonian-Messinian (10.0–5.3 Ma).

Comments: Though originally reported as having been recovered from the Early Pliocene Pico Formation [169], the holotype skull more likely derives from the basal part of the Towsley Formation [126], which correlates with the Delmontian benthic foraminiferal stage (7.7–5.2 Ma) [70, 172]. Strontium dates from near the base of the formation range give a best estimate of 6.3 ± 0.3 Ma, whereas samples from the overlying Pico Formation provide a minimum age estimate of 5.2 ± 0.3 or 5.5 ± 0.4 Ma [70]. Together, these dates imply late Messinian (6.3–5.3 Ma) for the holotype. The horizon of the Santa Margarita Formation that yielded UCMP 108558 likely dates to about 10–9 Ma, based on its associated mammal fauna [173: 26-27]. Together, these occurrences indicate an approximate age range of 10.0–5.3 Ma.

NMNZ MM001630

Locality and horizon: Taihape, North Island, New Zealand: Waiouru Formation [174].

Stratigraphic range: Zanclean (5.3–3.6 Ma).

Comments: The Waiouru Formation has been assigned to the Opoitian (5.3–3.6 Ma [54]), based on molluscan biostratigraphy [175].

OCPC 1178

Locality and horizon: Laguna Canyon, Orange County, USA: Vaqueros Formation [176]

Stratigraphic range: early–late Burdigalian (18.8–17.2 Ma).

Comments: The portion of the Vaqueros Formation that yielded this specimen has been correlated with chrons C5Er–C5En (18.8–18.1 Ma), or C5Dr–C5Dn (18.1–17.2 Ma [54]) based on magnetostratigraphic data and the occurrence of early Hemingfordian mammals in the underlying Sespe Formation [177]. This results in a total possible age range of 18.8–17.2 Ma, straddling the early–late Burdigalian boundary.

OU 22026

Locality and horizon: Hakataramea Valley, South Canterbury, New Zealand: lower to middle portion of the Kokoamu Greensand [178].

Stratigraphic range: early Chattian (27.3–26.0 Ma; **preliminary assessment**).

Comments: The Kokoamu Greensand largely correlates with the early Duntroonian [179], starting at 27.3 Ma [2]. The age of the top of the Kokoamu Greensand is constrained by an Sr date of ca. 26.0 Ma for the base of the overlying Otekaike Limestone [179].

OU 22044

Locality and horizon: “Earthquakes” locality near Duntroon, North Otago, New Zealand: lower part of the Maerewhenua Member of the Otekaike Limestone [180].

Stratigraphic range: late Chattian (25.4–25.3 Ma).

Comments: At the time of the analysis, the specimen was estimated to have been derived from an early Waitakian horizon of the Otekaike Limestone, indicating an age of roughly 25.2–23.0 Ma [2], and this was the age range use to calibrate the fossil tip. A subsequent analysis of the foraminifera associated with the specimen, however, suggests a somewhat older, Duntroonian age [180]. The lower portion of the Maerewhenua Member correlates with the uppermost Duntroonian, with strontium dates from the nearby Trig Z section indicating a narrow age range of ca. 25.4–25.3 Ma.

OU 22224

Locality and horizon: near Duntroon, North Otago, New Zealand: lower to middle portion of the Kokoamu Greensand [181].

Stratigraphic range: early Chattian (27.3–26.0 Ma; **preliminary assessment**).

Comments: The Kokoamu Greensand largely correlates with the early Duntroonian [179], starting at 27.3 Ma [2]. The age of the top of the Kokoamu Greensand is constrained by an Sr date of ca. 26.0 Ma for the base of the overlying Otekaike Limestone [179]

OU 22705

Locality and horizon: Bluecliffs section, Otaio River, South Canterbury, New Zealand: Mount Harris Formation.

Stratigraphic range: Aquitanian (21.7–20.5 Ma).

Comments: The fossil was recovered from near the top of the "cliff section", which is constrained by strontium age estimates of 21.7 Ma and 20.5 Ma [182].

OU GS 10897
Locality and horizon: near Oamaru, North Otago, New Zealand: lower Ototara Limestone [147].
Stratigraphic range: early Rupelian (33.0–32.0 Ma**; preliminary assessment**).
Comments: Originally referred to as a "prosqualodontid" [183], the specimen was later reassigned to *Llanocetus* sp. [147]. Based on its relative position within the Ototara Limestone, the horizon that yielded the specimen likely correlates with the early Whaingaroan, ca. 33.0–32.0 Ma [184].

Parabalaenoptera baulinensis
Specimen: CAS 66660 (holotype) [185].
Locality and horizon: Bolinas Point, Point Reyes Peninsula, California, USA: Santa Cruz Mudstone [185].
Stratigraphic range: late Tortonian–Messinian (7.6–6.7 Ma)
Comments: Diatoms recovered from the same stratum as the holotype correlate with "subzone a" of the *Nitzschia reinholdii* Zone [185], indicating an age range of 7.6–6.7 Ma [130, 172].

Parietobalaena palmeri
Specimens: USNM 10688 (holotype) [186, 187]; USNM 7424, 10677, 10909, 11535, 12697, 13874, 13903, 15576, 16119, 16568, 16570, 16667, 16838, 20376, 23015, 23022, 23055, 23203, 23448, 23725 [187].
Localities and horizons: USNM 23725, Holland Cliff, Patuxent River, near Deep Landing, Calvert County, Maryland, USA: bed 10 of the Calvert Formation; USNM 10677, 10909, 12697, 16667, 16838, near Plum Point Wharf, Calvert County, Maryland, USA: bed 10 of the Calvert Formation; USNM 11535, 13874, as before, but from bed 11 of the Calvert Formation; USNM 7424, 10688, near Dare's Wharf, Calvert County, Maryland, USA: bed 11 of the Calvert Formation; USNM 20376, as before, but bed unknown; USNM 13903, 16119, 16568, 16570, 23015, near mouth of Parker Creek, Calvert County, Maryland, USA: bed 12 of the Calvert Formation; USNM 23022, 23055, near road end at Governor Run, Calvert County, Maryland, USA: bed 13 of the Calvert Formation; USNM 23448, as before but from bed 14 of the Calvert Formation; USNM 23203, near mouth of Pope's Creek, Westmoreland County, Virginia, USA: Calvert Formation; USNM 15576, Calvert Beach, Calvert County, Maryland, USA: bed 16 of the Choptank Formation [187].
Stratigraphic range: late Burdigalian–Langhian (16.4–14.5 Ma)

Comments: Strontium dating indicates the age of bed 10 to be 16.2–15.8 Ma, or possibly somewhat older (16.4 Ma) [48, 49]. Bed 13 has been dated to span the Langhian–Serravallian boundary (ca. 14.4–13.7 Ma) [48] based on strontium dating, but was recently reassigned to the late Langhian (15.5–14.5 Ma), just after the end of the peak warmth associated with the Middle Miocene Climate Optimum [49]. Together, these estimates indicate an age range of 16.4–14.5 Ma. USNM 23448 and 15576 (from beds 14 and 16) only consist of incomplete postcranial material and an isolated tympanic bulla, and hence are not included here.

***Parietobalaena* sp.**

Specimen: SMNH VeF62 [188].
Locality and horizon: Hannya, Saitama Prefecture, Japan: Nagura Formation [188].
Stratigraphic range: late Burdigalian-Langhian (16.4–15.1 Ma)
Comments: The Nagura Formation forms the lowermost part of the Chichibumachi Group, which correlates with foraminiferal zone N8 [51, 110, 111] and thus spans the Burdigalian-Langhian boundary (16.4–15.1 Ma) [20, 54].

Parietobalaena yamaokai

Specimens: HMN F00022–00024 (holotype); HMN F00042–00054 (one individual); HMN F00127 and various other, assorted cranial and postcranial material [113]; HMN F00640 [189]; *Parietobalaena* cf. *yamaokai*, HMN-F00004 [190].
Localities and horizons: HMN F00022–24, 00042–00054, 00640 Monde-cho [113, 189]; HMN F00127, Nishihonmachi; other specimens are from these localities and Suketou, Kawate-cho; all localities are situated along the Saijyo River, Shobara City, Hiroshima Prefecture, Japan: Korematsu Formation [113]; HMN F00004, Saijyo River, Shobara, Hiroshima Prefecture, Japan: lower part of the Itabashi Formation [190].
Stratigraphic range: late Burdigalian–Langhian (17.0–14.9 Ma).
Comments: See *Diorocetus shobarensis* for details.

Pelocetus calvertensis

Specimens: USNM 11976 (holotype); USNM 14693, 21306, 23058 [191].
Localities and horizons: USNM 11976, near road at Governor Run, Calvert County, Maryland, USA: bed 13 or Calvert Formation; USNM 14593, near Chesapeake Beach Wharf, Calvert County, Maryland, USA: bed 13 of the Calvert Formation; USNM 21306, near mouth of Parker Creek, Calvert County, Maryland, USA: bed 17 of the Choptank Formation; USNM 23058, Stratford Cliffs, Westmoreland County, Virginia, USA: Calvert Formation [191].
Stratigraphic range: Langhian (15.5–14.5 Ma).

Comments: Bed 13 of the Calvert Formation has been strontium dated to the Langhian–Serravallian boundary (ca. 14.4–13.7 Ma) [48], but was recently reassigned to the late Langhian (15.5–14.5 Ma), just after the end of the peak warmth associated with the Middle Miocene Climate Optimum [49]. The referred specimens are likely non-diagnostic (e.g. USNM 23106 consists only of an anterior end of a mandible), and hence not included here. A further specimen, USNM 23059, was originally referred to *P. calvertensis* by Kellogg [191], but was subsequently identified as a different taxon related to *Diorocetus* [192].

Peripolocetus vexillifer

Specimen: CAS 905.22 (formerly CAS 4379; holotype); CAS 4358, 4387, 4389 [93].
Locality and horizon: Sharktooth Hill, Kern County, California, USA: Sharktooth Hill bone bed of the Round Mountain Silt/ Temblor Formation [93].
Stratigraphic range: Langhian (16.0–15.2 Ma).
Comments: The Round Mountain Silt has been dated to 16.1–14.5 Ma based on magnetostratigraphy, strontium dating, and diatoms indicative of the *Denticulopsis lauta* A biozone (16–15 Ma). More specifically, the middle portion of Round Mountain Silt (including the bone bed) has been correlated with Chron C5Br, indicating an age range of 16.0–15.2 Ma [193].

Pinocetus polonicus

Specimen: MZ VIII_Vm-750 (holotype) [194].
Locality and horizon: Nowa Wieś, near Pińczów, Poland: Pińczów Formation [194].
Stratigraphic range: Langhian (15.1–13.8 Ma).
Comments: The Pińczów Formation has been assigned to the early–middle Badenian based on foraminiferal data [195, 196], and is constrained by the occurrence of *Orbulina suturalis* (first appearing at 15.1 Ma [20]) and its correlation with the Lagenidae benthic foraminiferal zone [197], which terminates at or near the Langhian-Serravallian boundary [198], i.e. 13.8 Ma [54].

Piscobalaena nana

Specimens: SMNK PAL4050 (holotype); MNHN SAS892, 1616–1618, 1623, 1624; MNHN PPI259, 260 [74]; SMNS, no number (Pilleri collection no. 88) [199]
Localities and horizons: SMNK PAL4050, MNHN SAS892, 1616–1618, 1623, 1624, SMNS no number, Sud-Sacaco, Peru; MNHN PPI259, 260, Aguada de Lomas, Peru; all specimens are from the Pisco Formation [74].
Stratigraphic range: late Tortonian-Messinian (7.5–5.9 Ma)
Comments: Though originally thought to date to the Early Pliocene [74], recent work assigned Sud-Sacaco to the Late Miocene (7.1–5.9 Ma, Messinian) based on $^{87}Sr/^{86}Sr$

dating [167]. Agua de Lomas is somewhat older (7.5–7.3 Ma, based on K-Ar and $^{87}Sr/^{86}Sr$ dating [74, 75, 167]), resulting in a total age range of 7.5–5.9 Ma.

Plesiobalaenoptera quarantellii

Specimen: MPST/SBAER 240505 (holotype) [200].

Locality and horizon: Western portion of a bend in the Stirone River, approximately 2 km northwest of Salsomaggiore Terme, Italy: formation not stated [200].

Stratigraphic range: late Tortonian (11.6–9.44 Ma).

Comments: The holotype was recovered from grey, clayey marls bearing invertebrate fossils indicative of a Tortonian age [200]. According to Artoni et al. [201: fig. 2], the rocks exposed along this section of the river where the specimen was found form part of a middle Eocene–early Tortonian Epiligurian succession incorporated into an “Intra-Messinian Chaotic Unit”. Pending the publication of further data on the exact age of these strata, *P. quarantellii* is therefore here assumed to be early Tortonian (11.6–9.44 Ma).

Thinocetus arthritus

Specimen: USNM 23794 (holotype) [202].

Locality and horizon: Mud Cliffs, south shore of Potomac River, near Maryland/Virginia border, Westmoreland County, Virginia, USA: bed 17 of the Choptank Formation [202].

Stratigraphic range: Serravallian (13.5–13.4 Ma)

Comments: The Choptank Formation as a whole falls into dinoflagellate zone DN6 [94], which in turn partially correlates with foraminiferal zones N10–11 (14.2–13.4 Ma [20]) and nannoplankton zone NN6 (13.5–11.9 Ma [20]) [94]. The overlap of these zones defines a narrow age range of 13.5–13.4 Ma, which fits well with strontium dates of ca. 13 Ma [48, 49], and the 13.9–13.8 Ma expansion of the East Antarctic Ice Sheet interpreted to mark the transition between the Calvert and Choptank formations [49].

Tiphyocetus temblorensis

Specimens: CAS 905.01 (formerly CAS 4355, holotype); CAS 4353, 4354, 4356, 4357, 4373–4375, 4376, 4378, 4388, 4390, 4391, 4393, 4425, 4436, 4438–4440, 4442, 4443 [93].

Locality and horizon: Sharktooth Hill, Kern County, California, USA: Sharktooth Hill bone bed of the Round Mountain Silt/ Temblor Formation [93].

Stratigraphic range: Langhian (16.0–15.2 Ma)

Comments: See *Peripolocetus vexillifer* for details.

Titanocetus sammarinensis

Specimen: MGB 1CMC172 9073 (holotype) [203].

Locality and horizon: Near top of Monte Titano, Republic of San Marino: Monte Fumaiolo Formation [203].
Stratigraphic range: late Burdigalian–Langhian (16.4–14.7 Ma)
Comments: The Monte Fumaiolo Formation has been correlated with two foraminiferal zones [204, 205]: (1) the *Praeorbulina glomerosa* zone (subzone *P. sicana*, starting at 16.4 Ma [20, 206]); and (ii) the *Orbulina suturalis-Globorotalia* (*Fohsella*) *peripheroronda* (subzone *O. suturalis*, 15.1–14.7 Ma [20, 206]), giving a total age range of 16.4–14.7 Ma.

Uranocetus gramensis
Specimen: MSM p813 (holotype) [207].
Locality and horizon: Gram Clay Pit, Gram, South Jutland, Denmark: uppermost portion of the Gram Formation [207].
Stratigraphic range: late Tortonian (8.6–7.5 Ma)
Comments: The uppermost portion of the Gram Formation correlates with foraminiferal zone N17 (8.6–5.7 Ma [20, 54]) [208] and – within the clay pit –North Sea foraminiferal zone NSB 13b [209] and dinoflagellate zone D19b (8.7–7.5 Ma [54]), as judged from the last appearance of *Spiniferites pseudofurcatus* [208, 210]. Together, these dates imply a late Tortonian age (8.6–7.5 Ma).

UWBM 84024
Locality and horizon: west of Shipwreck Point, Clallam County, Olympic Peninsula, Washington, USA: Jansen Creek Member of the Makah Formation, or strata just below [211].
Stratigraphic range: early Rupelian (33.2–31.0 Ma).
Comments: Based on magnetostratigraphic, foraminiferal, and molluscan evidence, the marine mammal-bearing part of the Makah Formation (Jansen Creek Member and adjacent layers) has been correlated with the early Zemorrian Chron C12r [212], indicating an age of 33.3–31.0 Ma [12].

Yamatocetus canaliculatus
Specimen: KMNH VP000017 [213].
Locality and horizon: Tominohana Cape, Wakamatsu Ward, Kitakyushu City, Kyushu, Japan: Jinnobaru Formation [213].
Stratigraphic range: late Rupelian (29.2–28.1 Ma).
Comments: There is no direct dating evidence for the Jinnobaru Formation. However, fission track dating places the underlying Norimatsu Formation at 30.3 ± 1.2 Ma [214]. Foraminifera sampled above and below the Jinnobaru Formation place the latter in zone P21a/ subzone O4 [215-217], thus implying a latest Rupelian age (29.2–28.1 Ma [12, 20]).

ZMT 67

Locality and horizon: Hakataramea Valley, South Canterbury, New Zealand: upper Otekaike Limestone.

Stratigraphic range: Aquitanian (23.0–21.7 Ma)

Comments: Associated foraminifera indicate that this specimen falls into the *Globigerina woodi connecta* zone (at or shortly above the Oligocene–Miocene boundary, ca. 23.0 Ma [182, 218]), but predates the appearance of *Ehrenbergina marwicki* at 21.7 Ma [182].

Institutional abbreviations

ALMNH, Alabama Museum of Natural History, Tuscaloosa, USA; AMNH, American Museum of Natural History, New York, USA; AMP, Ashoro Museum of Paleontology, Ashoro, Hokkaido, Japan; CAS, California Academy of Sciences, San Francisco, USA; ChM, The Charleston Museum, Charleston, USA; CMN, Canadian Museum of Nature, Ottawa, Canada; FMNH, The Field Museum of Natural History, Chicago, USA; GMNH, Gunma Museum of Natural History, Tomioka, Japan; HMN, Hiwa Museum of Natural History, Hiwa, Japan; IFAW(CCSN), International Fund for Animal Welfare Marien Rescue and Research Team, formerly Cape Cod Stranding Network, Massachusetts, USA; IRSNB, Institut Royal des Sciences Naturelles de Belgique, Brussels, Belgium; KMNH, Kitakyushu Museum of Natural and Human History, Kitakyushu, Kyushu, Japan; LACM, Natural History Museum of Los Angeles County, Los Angeles, USA; MB, Museum für Naturkunde, Berlin, Germany; MCZ, Museum of Comparative Zoology (Harvard University), Cambridge, USA; MFM, Mizunami Fossil Museum, Gifu, Japan; MGB, Museo Geopalaeontologico G. Capellini, Bologna, Italy; MGPC, Museo Geopaleontologico 'Giuseppe Cortesi', Castell'Arquato, Italy; MLP, Museo de La Plata, La Plata, Argentina; MNHN, Museum National d`Histoire Naturelle, Paris, France; MPST, Museo Paleontologico di Salsomaggiore Terme, Italy; MRSN, Museo Regionale di Scienze Naturali, Turin, Italy; MSM, Museum Sønderjylland, Gram, Denmark; MSNTUP, Museo di Storia Naturale e del Territorio, Università di Pisa, Pisa, Italy; MZ, Museum of the Earth, Polish Academy of Sciences, Warsaw, Poland; NBM, New Brunswick Museum, Saint John, Canada; NEAQ, New England Aquarium, Boston, USA; NHG, Natuurhistorische collectie van het Zeeuws Genootschap der Wetenschappen, Middelburg, the Netherlands; NHMUK, The Natural History Museum, London, United Kingdom; NMB, Natuurmuseum Brabant, Tilburg, The Netherlands; NMNH, Academician V. A. Topachevsky Paleontological Museum (National Museum of Natural History, National Academy of Sciences of Ukraine), Kiev, Ukraine; NMNS, National Museum of Nature and Science, Tokyo/Tsukuba, Japan; NMNZ, Museum of New Zealand Te Papa Tongarewa, Wellington,

New Zealand; NMV, Museum Victoria, Melbourne, Australia; NMW, National Museum of Wales Amgueddfa Cymru, Cardiff, Wales, United Kingdom; OCPC, The Cooper Center, Orange County, USA; OM, Otago Museum, Dunedin, New Zealand; Osaka Museum of Natural History, Osaka, Japan; OU, Geology Museum, University of Otago, Dunedin, New Zealand; PIN, Paleontological Institute, Moscow; SBAER, Soprintendenza per i Beni Archeologici dell'Emilia Romagna; SDNHM, San Diego Natural History Museum, San Diego, USA; SFM, Shinshushinmachi Fossil Museum, Shinshushinmachi, Japan; SMAC, Sapporo Museum Activities Center, Sapporo, Japan; SMNH, Saitama Museum of Natural History, Saitama, Japan; SMBP, Mystic Aquarium, Mystic, USA; SMNK, Staatliches Museum für Naturkunde, Karlsruhe, Germany; SMNS, Staatliches Museum für Naturkunde, Stuttgart, Germany; TNU, Department of Zoology, Taurida National University, Simferopol, Ukraine; UCMP, University of California Museum of Paleontology, Berkeley, USA; UNCW, University of North Carolina Wilmington, Wilmington, USA; UO, Museum of Natural History, University of Oregon, Eugene, Oregon, USA; USNM, United States National Museum of Natural History, Washington DC, USA; UWBM, Burke Museum of Natural History and Culture, Seattle, USA; VMSM, Virginia Marine Science Museum, Virginia Beach, USA; VMW, Sierra College Natural History Museum, Rocklin, USA; ZMT, Fossil mammals catalogue, Canterbury Museum, Christchurch, New Zealand.

List of studied material

In the following list, a species or specimen marked with an asterisk was scored for the phylogenetic analysis but not illustrated on MorphoBank owing to its ongoing description or re-description by other workers.

Outgroup:

Archaeodelphis patrius Allen, 1921: NMNS PV22239 (cast of MCZ 15749)

Physeter catodon Linnaeus, 1758: AMNH 34872; MSNTUP M266; NBM 001850; NMNS M24821, M32579, M34048; OMNH M3000; USNM 253051

Waipatia maerewhenua Fordyce, 1994: OU 22095

Zygorhiza kochii Carus, 1847: ALMNH 2000.1.2.1; USNM 4678, 4679, 11962, 12063, 16438, 16639, 537887

Zygorhiza sp.[14] OU 22100, 22222-1, 22242

Ingroup:

Aetiocetus cotylalveus Emlong, 1966: USNM 25210; photos of USNM 25210 provided by E. Fitzgerald

Aetiocetus polydentatus Sawamura, 1995: AMP 12

Aetiocetus weltoni Barnes & Kimura, 1995: UCMP 122900

Aglaocetus moreni Lydekker, 1894: FMNH P13407

Aglaocetus patulus Kellogg, 1968c: USNM 13472, 23690

Archaebalaenoptera castriarquati Bisconti, 2007a: MGPC/SBAER 240536

Balaena montalionis Capellini, 1904: MSNTUP I12357

Balaena mysticetus Linnaeus, 1758: IRSNB 1532; LACM 54479, 072159, 072490b; NHMUK 1934.10.10.1; NMNS M25893; USNM 15695, 63300, 63301, 291101, 255992

Balaena ricei Westgate and Whitmore, 2002: USNM 22553

Balaenella brachyrhynus Bisconti, 2005: NMB 42001

Balaenoptera acutorostrata Lacepede, 1804: IFAW(CCSN) 04-152; ChM CM1183; MSNTUP M260; NMNS M5152, M24357, M25927; SDNHM 23642; USNM 61715

Balaenoptera bertae Boessenecker, 2013: UCMP 21907

Balaenoptera bonaerensis Burmeister, 1867: NMNS M19792; OM VT3057, VT3060

Balaenoptera borealis Lesson, 1828: LACM 054505; NMNS M03536, M25908; SMNS 26443; USNM 236680, 486174, 504244, 504699, 571340

Balaenoptera musculus Linnaeus, 1758: MSNTUP M250; NMNS M25900, M29617; USNM 49786, 124326, 239280, 267614

**Balaenoptera omurai* Wada, 2003: NMNS M32505, M32992

Balaenoptera physalus Linnaeus, 1758: MSNTUP M251; NEAQ 12-187 Bp; NMNS M25904; OM VT2570; OMNH M1000, no number; SMBP 0448; USNM 16039, 49780, 237566, A16045

“Balaenoptera” portisi Sacco, 1890: MRSN PU13808; UCMP 219135

**”Balaenoptera” ryani* Hanna and McLellan, 1924: CAS 1733

Balaenoptera siberi Lacepede, 1804: SMNK no number (holotype); SMNS 47307

Balaenula astensis Trevisan, 1942: MSNTUP I12555

**Balaenula* sp. [79]: SMAC 1309

Brandtocetus chongulek Gol'din & Startsev, 2014: TNU skulls 2, 4, A

Caperea marginata Gray, 1846b: NMNZ MM002064, MM002119, MM002232, MM002235, MM002254, MM002262, MM002898, MM002900, TMP011345; NMV C28531; OM VT227

**Cephalotropis coronatus* Cope, 1896: USNM 489194

"Cetotherium" megalophysum Cope, 1895: USNM 10593, 22962, 205510

Cetotherium rathkii Brandt, 1843: PIN 1840/1

Cetotherium riabinini Hofstein, 1948: NMNH P668/1

Chonecetus goedertorum Barnes and Furusawa, 1995: LACM 131146, 138027

Chonecetus sookensis Russell, 1968: CMN FV64443

Diorocetus chichibuensis Yoshida, Kimura & Hasegawa, 2003: SMNH VeF19, VeF68

Diorocetus hiatus Kellogg, 1968: USNM 16783, 23494

Diorocetus shobarensis Otsuka & Ota, 2008: HMN F00005–00058 (one individual), F00063

Diunatans luctoretemergo Bosselaers and Post, 2010: NHG 22279, 22347

Eomysticetus whitmorei Sanders and Barnes, 2002: ChM PV4253

Eschrichtioides gastaldii Strobel, 1881: MRSN PU13802

Eschrichtius robustus Van Beneden & Gervais, 1868: AMP R09; NMNS M15940, M25899, M34025; USNM 13803, 364973, 364975

Eubalaena belgica Abel, 1941: IRSNB M879a-f

Eubalaena spp.

(*E. australis* Desmoulins, 1822): NMNZ MM000226, MM002239; NMV C28603; OU, no number; USNM 267612

(*E. glacialis* Müller, 1776): FMNH 15559; MSNTUP M264; USNM A23077, 504886, no number; UNCW KLC 022; VMSM 2004-1004

(*E. japonica* Lacépède, 1818): NMNS 33436

Eubalaena shinshuensis Kimura, Narita, Fujita & Hasegawa, 2007: SFM CV0024

Gricetoides aurorae Whitmore and Kaltenbach, 2008: USNM 25762, 182921, 183004, 244468

Herpetocetus bramblei Whitmore and Barnes, 2008: UCMP 82465, 219079, 219111, 219112

Herpetocetus morrowi El Adli, Deméré & Boessenecker, 2014: SDNHM 23057, 32138, 35294, 38689, 63096, 63690, 65781, 83694, 90484, 130390; UCMP 124950

Herpetocetus sp.[8]: VMW 65

Herpetocetus transatlanticus Whitmore and Barnes, 2008: USNM 182962

Isanacetus laticephalus Kimura and Ozawa, 2002: MFM 18004, 28501

Janjucetus hunderi Fitzgerald, 2006: NMV P216929, P229455; USNM 534009

Joumocetus shimizui Kimura and Hasegawa, 2010: GMNH PV2401

Kurdalagonus mchedlidzei Tarasenko and Lopatin, 2012: NMRA 10476

**Llanocetus denticrenatus* Mitchell, 1989: USNM 183022

Mammalodon colliveri Pritchard, 1939: NMV P199986

Mauicetus parki Benham, 1937: OU 11573, *22545

"Megaptera" hubachi Dathe, 1983: MB Ma28570

"Megaptera" miocaena Kellogg, 1922: USNM 10300

Megaptera novaeangliae Brisson, 1762: IFAW(CCSN) 07-181, 10-188Mn; MSNTUP M263; NEAQ 05604; NMNS M33734; NMNZ MM000228; NMW Z.1982.058; USNM 13656, 269982, 301636

Metopocetus durinasus Cope, 1896: USNM 8518

Micromysticetus rothauseni Sanders and Barnes, 2002a: ChM PV4844

Miocaperea pulchra Bisconti, 2012: SMNS 46978

Morawanocetus yabukii Kimura and Barnes, 1995: AMP 01, 14

**Morenocetus parvus* Cabrera, 1926: MLP 5-11, 5-15

Nannocetus eremus Kellogg, 1929: UCMP 26502

Parabalaenoptera baulinensis Zeigler, Chan & Barnes, 1997: CAS 66660

Parietobalaena campiniana Bisconti, Lambert & Bosselaers, 2013: IRSNB M.399-R.4018

Parietobalaena palmeri Kellogg, 1924: USNM 10668, 10677, 11535, 16119

Parietobalaena sp. [188]: SMNH VeF62

Parietobalaena yamaokai Otsuka and Ota, 2008: HMN F00022, F00042, F00044, F00054, F00064/65, F00127, F00640

Pelocetus calvertensis Kellogg, 1965: USNM 11976

**Peripolocetus vexillifer* Kellogg, 1931: CAS 905.22

Pinocetus polonicus Czyźewska & Ryziewicz, 1976: MZ VIII/Vm-750

Piscobalaena nana Pilleri and Siber, 1989: MNHN SAS892, SAS1616, SAS1617, SAS1618; SMNK Pal4050

Plesiobalaenoptera quarantellii Bisconti, 2010: MPST/SBAER 240505

Thinocetus arthritus Kellogg, 1969: USNM 23794

Tiphyocetus temblorensis Kellogg, 1931: CAS 905.01

Titanocetus sammarinensis Bisconti, 2006: MGB 1CMC172 9073, 1CMC172 9072 (cast of MGB 1CMC172 9073)

Uranocetus gramensis Steeman, 2009: MSM p813

Yamatocetus canaliculatus Okazaki, 2012: KMNH VP000017

<u>Undescribed taxa:</u>

*ChM PV4745, archaic toothed mysticete [101]

*NMNZ MM001630, balaenopterid [174]

*OCPC 1178, aetiocetid [176]

*OU 22026, *Mammalodon* sp. [178]

*OU 22044, eomysticetids [219]

*OU 22224, Oligocene balaenid [181]

*OU 22705, archaic chaeomysticete

*OU GS10897, archaic toothed mysticete, “protosqualodont” of Keyes [183], and *Llanocetus* sp. of Fordyce [147]

*UWBM 84024, aetiocetid

*ZMT 67, archaic chaeomysticete [220]

Supplementary References

1 . Fordyce, RE. 1994 *Waipatia maerewhenua*, new genus and new species (Waipatiidae, new family), an archaic Late Oligocene dolphin (Cetacea: Odontoceti: Platanistoidea) from New Zealand. *Proc San Diego Soc Nat Hist*. **29**, 147–176.

2 . Hollis, CJGNSS. 2010 Calibration of the New Zealand Cretaceous-Cenozoic timescale to GTS2004. *GNS Sci Rep*. **2010/43**, 1-20.

3 . Bisconti, M. 2007 A new basal balaenopterid whale from the pliocene of northern Italy. *Palaeontology*. **50**, 1103-1122. (10.1111/j.1475-4983.2007.00696.x)

4 . Bisconti, M. 2000 New description, character analysis and preliminary phyletic assessment of two Balaenidae skulls from the Italian Pliocene. *Palaeontogr Ital*. **87**, 37-66.

5 . Boessenecker, RW. 2013 A new marine vertebrate assemblage from the Late Neogene Purisima Formation in Central California, part II: Pinnipeds and Cetaceans. *Geodiversitas*. **35**, 815-940.

6 . Bosselaers, M, Post, K. 2010 A new fossil rorqual (Mammalia, Cetacea, Balaenopteridae) from the Early Pliocene of the North Sea, with a review of the rorqual species described by Owen and Van Beneden. *Geodiversitas*. **32**, 331-363. (10.5252/g2010n2a6)

7 . El Adli, JJ, Deméré, TA, Boessenecker, RW. 2014 *Herpetocetus morrowi* (Cetacea: Mysticeti), a new species of diminutive baleen whale from the Upper Pliocene (Piacenzian) of California, USA, with observations on the evolution and relationships of the Cetotheriidae. *Zool J Linn Soc Lond*. **170**, 400-466. (10.1111/zoj.12108)

8 . Boessenecker, RW. 2013 Pleistocene survival of an archaic dwarf baleen whale (Mysticeti: Cetotheriidae). *Naturwissenschaften*. **100**, 365-371. (10.1007/s00114-013-1037-2)

9 . Bisconti, M, Lambert, O, Bosselaers, M. 2013 Taxonomic revision of *Isocetus depauwi* (Mammalia, Cetacea, Mysticeti) and the phylogenetic relationships of archaic cetothere' mysticetes. *Palaeontology*. **56**, 95-127. (10.1111/j.1475-4983.2012.01168.x)
10 . Allen, GM. 1921 A new fossil cetacean. *Bull Mus Comp Zool*. **65**, 1-13.
11 . Uhen, MD, Fordyce, RE, Barnes, LG. 2008 Odontoceti. In *Evolution of Tertiary Mammals of North America*. (ed.^eds. C. M. Janis, G. F. Gunnell, M. D. Uhen), pp. 566–606. Cambridge: Cambridge University Press.
12 . Vandenberghe, N, Hilgen, FJ, Speijer, RP. 2012 The Paleogene Period. In *The Geologic Time Scale 2012*. (ed.^eds. F. M. Gradstein, J. G. Ogg, M. Schmitz, G. Ogg), pp. 855–921. Oxford: Elsevier.
13 . Williams, GL, Brinkhuis, H, Pearce, MA, Fensome, RA, Weegink, JW. 2004 Southern Ocean and global dinoflagellate cyst events compared: index events for the Late Cretaceous–Neogene. *Proc Ocean Drill Program Sci Results*. **189**, 1–98.
14 . Köhler, R, Fordyce, RE. 1997 An archaeocete whale (Cetacea: Archaeoceti) from the Eocene Waihao Greensand, New Zealand. *J Vertebr Paleontol*. **17**, 574-583. (10.1080/02724634.1997.10011004)
15 . Srinivasan, MS. 1966 Foraminifera and age of the type section of the Tahuian Stage. *N Z J Geol Geophys*. **9**, 504-512. (10.1080/00288306.1966.10422495)
16 . Morgans, HEG. 2009 Late Paleocene to middle Eocene foraminiferal biostratigraphy of the Hampden Beach section, eastern South Island, New Zealand. *N Z J Geol Geophys*. **52**, 273-320. (10.1080/00288306.2009.9518460)
17 . Uhen, MD. 2013 A review of North American Basilosauridae. *Bull Alabama Mus Nat Hist*. **31**, 1-45.
18 . Mancini, EA, Waters, LA. 1986 Planktonic foraminiferal biostratigraphy of upper Eocene and lower Oligocene strata in southern Mississippi and southwestern and south-central Alabama. *The Journal of Foraminiferal Research*. **16**, 24-33. (10.2113/gsjfr.16.1.24)
19 . Berggren, WA, Kent, DV, Swisher, CC, III, Aubry, MP. 1995 A revised Cenozoic geochronology and chronostratigraphy. *SEPM Spec Publ*. **54**, 129-212.
20 . Anthonissen, DE, Ogg, JG. 2012 Cenozoic and Cretaceous biochronology of planktonic foraminifera and calcareous nannofossils. In *The Geologic Time Scale 2012*. (ed.^eds. F. M. Gradstein, J. G. Ogg, M. Schmitz, G. Ogg), pp. 1083–1127. Oxford: Elsevier.
21 . Emlong, D. 1966 A new archaic cetacean from the Oligocene of Northwest Oregon. *Bull Mus Nat Hist Univ Oregon*. **3**, 1-51.
22 . Deméré, TA, Berta, A. 2008 Skull anatomy of the Oligocene toothed mysticete *Aetioceus weltoni* (Mammalia; Cetacea): Implications for mysticete evolution and functional anatomy. *Zool J Linn Soc Lond*. **154**, 308-352. (10.1111/j.1096-3642.2008.00414.x)

23 . USNM. Museum Collection Records. 2014 [cited; Available from: http://collections.mnh.si.edu/search/mammals/
24 . Barnes, LG, Kimura, M, Furusawa, H, Sawamura, H. 1995 Classification and distribution of Oligocene Aetiocetidae (Mammalia; Cetacea; Mysticeti) from western North America and Japan. *Isl Arc*. **3**, 392-431. (10.1111/j.1440-1738.1994.tb00122.x)
25 . Prothero, DR, Bitboul, CZ, Moore, GW, Niem, AR. 2001 Magnetic stratigraphy and tectonic rotation of the Oligocene Alsea, Yaquina, and Nye Formations. *Pacific Section SEPM Book*. **91**, 184–194.
26 . Snavely, PD, MacLeod, NS, Rau, WW, Addicott, WO, Pearl, JF. 1975 Alsea Formation, an Oligocene marine sedimentary sequence in the Oregon Coast Range. *US Geol Surv Bull*. **1395-F**, 1–21
27 . Prothero, DR. 2003 Pacific coast Eocene-Oligocene marine chronostratigraphy: a review and an update. In *From Greenhouse to Icehouse - The Marine Eocene-Oligocene Transition*. (ed.^eds. D. R. Prothero, L. C. Ivany, N. E. Nesbitt), pp. 1–13. New York: Columbia University Press.
28 . Orr, WN, Faulhaber, JA. 1975 Middle Tertiary cetacean from Oregon. *Northwest Sci*. **49**, 174–181.
29 . Orr, WN, Miller, PR. 1983 Fossil Cetacea (whales) in the Oregon Western Cascades. *Oreg Geol*. **45**, 95–98.
30 . Miller, PR, Orr, WN. 1986 The Scott Mills Formation: mid-Tertiary geologic history and paleogeography of the central Western Cascade Range, Oregon. *Oreg Geol*. **48**, 139–151.
31 . Miller, PR, Orr, WN. 1988 Mid-Tertiary transgressive rocky coast sedimentation: central Western Cascade Range, Oregon. *J Sediment Petrol*. **58**, 959–968.
32 . Retallack, JG, Orr, NW, Prothero, RD, Duncan, AR, Kester, RP, Ambers, PC. 2004 Eocene-Oligocene extinction and paleoclimatic change near Eugene, Oregon. *Geol Soc Am Bull*. **116**, 817–839.
33 . Saito, T, Barron, JA, Sakamoto, M. 1988 An early Late Oligocene age indicated by diatoms for a primitive desmostylians mammal *Behemotops* from eastern Hokkaido, Japan. *Proc Jpn Acad Ser B*. **64**, 269–273.
34 . Gladenkov, AY. 1998 Oligocene and lower Miocene diatom zonation in the North Pacific. *Stratigr Geol Correl*. **6**, 150–163.
35 . Gladenkov, AY. 2006 The Cenozoic diatom zonation and its significance for stratigraphic correlations in the North Pacific. *Paleontol J*. **40**, S571-S583. (10.1134/s0031030106110049)
36 . Gladenkov, AY. 2008 The North Pacific advanced Oligocene to lower Miocene diatom stratigraphy. *Bull Geol Surv Jpn*. **59**, 309–318.

37 . Matsui, M, Ganzawa, Y. 1987 The Oligocene-Miocene Kawakami Group in eastern Hokkaido, with reference to the host strata and the age of the Ashoro fauna. In *Commemorative Volume for Prof. M. Matsui* (ed.^eds. pp. 137–143. Sapporo: Committee for the Publication of the Commemorative Volume for Prof. Matsui.

38 . Ganzawa, Y, Honda, T, Nozaki, T. 1990 Absolute measurements of thermal neutron fluence and fission track dating. *Int J Radiat Appl Instrum Part D Nucl Tracks Radiat Meas*. **17**, 273-276. (http://dx.doi.org/10.1016/1359-0189(90)90046-Z)

39 . Lydekker, R. 1894 Cetacean skulls from Patagonia. *Anal Mus La Plata*. **2**, 1-13.

40 . Cabrera, A. 1926 Cetáceos fósiles del Museo de La Plata. *Rev Mus La Plata*. **29**, 363–411.

41 . Kellogg, R. 1934 The Patagonian fossil whalebone whale, *Cetotherium moreni* (Lydekker). *Carnegie Inst Wash Publ*. **447**, 64–81.

42 . Cione, AL, Cozzuol, MA. 1990 Reidentification of *Portheus patagonicus* Ameghino, 1901, a supposed fish from the middle Tertiary of Patagonia, as a delphinoid cetacean. *J Paleontol*. **64**, 451-453.

43 . Scasso, RA, Castro, LN. 1999 Cenozoic phosphatic deposits in North Patagonia, Argentina: Phosphogenesis, sequence-stratigraphy and paleoceanography. *J South Am Earth Sci*. **12**, 471-487. (http://dx.doi.org/10.1016/S0895-9811(99)00035-8)

44 . Cozzuol, MA. 1996 The record of the aquatic mammals on southern South America. *Münchner Geowiss Abh*. **30**, 321-342.

45 . Cione, AL, Cozzuol, MA, Dozo, MT, Acosta Hospitaleche, C. 2011 Marine vertebrate assemblages in the southwest Atlantic during the Miocene. *Biol J Linn Soc*. **103**, 423-440. (10.1111/j.1095-8312.2011.01685.x)

46 . Dunn, RE, Kohn, MJ, Madden, RH, Strömberg, CE, Carlini, AA. High Precision U/Pb Geochronology of Eocene-Miocene South American Land Mammal Ages at Gran Barranca, Argentina. *Fall Meeting of American Geophysical Union*. San Francisco 2009:Abstract GP23B-0791.

47 . Kellogg, R. 1968 Fossil marine mammals from the Miocene Calvert Formation of Maryland and Virginia, part 7: a sharp-nosed cetothere from the Miocene Calvert. *US Nat Mus Bull*. **247**, 163–173.

48 . Browning, JV, Miller, KG, McLaughlin, PP, Kominz, MA, Sugarman, PJ, Monteverde, D, Feigenson, MD, Hernández, JC. 2006 Quantification of the effects of eustasy, subsidence, and sediment supply on Miocene sequences, mid-Atlantic margin of the United States. *Geol Soc Am Bull*. **118**, 567-588. (10.1130/b25551.1)

49 . Vogt, PR, Parrish, M. 2012 Driftwood dropstones in Middle Miocene Climate Optimum shallow marine strata (Calvert Cliffs, Maryland Coastal Plain): Erratic pebbles no certain

proxy for cold climate. *Palaeogeogr Palaeoclimatol Palaeoecol*. **323–325**, 100-109. (http://dx.doi.org/10.1016/j.palaeo.2012.01.035)

50 . Westgate, JW, Whitmore, FC. 2002 *Balaena ricei*, a new species of bowhead whale from the Yorktown Formation (Pliocene) of Hampton, Virginia. *Smithson Contrib Paleobiol*. **93**, 295–312.

51 . Blow, WH. 1969 Late Middle Eocene to Recent planktonic foraminiferal biostratigraphy. In *Proceedings of the First International Conference on Planktonic Microfossils*. (ed.^eds. P. Brönnimann, H. H. Renz), pp. 199–422. Leiden: E. J. Brill.

52 . Hazel, JE. 1971 Ostracode biostratigraphy of the Yorktown Formation (Upper Miocene and Lower Pliocene) of Virginia and North Carolina. *US Geol Surv Prof Pap*. **704**, 1–13.

53 . Hazel, JE. 1983 Age and correlation of the Yorktown (Pliocene) and Croata (Pliocene and Pleistocene) Formations at the Lee Creek Mine. *Smithson Contrib Paleobiol*. **53**, 81–200.

54 . Hilgen, FJ, Lourens, LJ, Van Dam, JA. 2012 The Neogene Period. In *The Geologic Time Scale 2012*. (ed.^eds. F. M. Gradstein, J. G. Ogg, M. Schmitz, G. Ogg), pp. 923–978. Oxford: Elsevier.

55 . Browning, JV, Miller, KG, McLaughlin, PP, Edwards, LE, Kulpecz, AA, Powars, DS, Wade, BS, Feigenson, MD, Wright, JD. 2009 Integrated sequence stratigraphy of the postimpact sediments from the Eyreville core holes, Chesapeake Bay impact structure inner basin. *Geol Soc Am Spec Pap*. **458**, 775-810. (10.1130/2009.2458(33))

56 . Bisconti, M. 2005 Skull morphology and phylogenetic relationships of a new diminutive balaenid from the Lower Pliocene of Belgium. *Palaeontology*. **48**, 793-816. (10.1111/j.1475-4983.2005.00488.x)

57 . De Schepper, S, Head, MJ, Louwye, S. 2009 Pliocene dinoflagellate cyst stratigraphy, palaeoecology and sequence stratigraphy of the Tunnel-Canal Dock, Belgium. *Geol Mag*. **146**, 92-112. (doi:10.1017/S0016756808005438)

58 . Portis, A. 1885 Catalogo descrittivo dei Talassoterii rinvenuti nei terreni terziari del Piemonte e della Liguria. *Mem R Accad Sci Torino*. **1885**, 247–365.

59 . Deméré, TA, Berta, A, McGowen, MR. 2005 The taxonomic and evolutionary history of fossil and modern balaenopteroid mysticetes. *J Mamm Evol*. **12**, 99-143. (10.1007/s10914-005-6944-3)

60 . Morgan, GA. 1994 Miocene and Pliocene marine mammal faunas from the Bone Valley Formation. *Proc San Diego Soc Nat Hist*. **29**, 239-268.

61 . Ferrero, E, Pavia, G. 1996 La successione marina previllanoviana. *Il Quaternario* **9**, 36–38

62 . Bisconti, M. 2008 Morphology and phylogenetic relationships of a new eschrichtiid genus (Cetacea : Mysticeti) from the Early Pliocene of northern Italy. *Zool J Linn Soc Lond*. **153**, 161-186. (10.1111/j.1096-3642.2008.00374.x)
63 . Monegatti, P, Raffi, S. 2001 Taxonomic diversity and stratigraphic distribution of Mediterranean Pliocene bivalves. *Palaeogeogr Palaeoclimatol Palaeoecol*. **165**, 171-193. (http://dx.doi.org/10.1016/S0031-0182(00)00159-0)
64 . Raffi, S, Monegatti, P. 1993 Bivalve taxonomic diversity throughout the Italian Pliocene as a tool for climatic-oceanographic and stratigraphic inferences. *Ciênc Terra*. **12**, 45–50.
65 . Violanti, D. 2012 Pliocene Mediterranean foraminiferal biostratigraphy: a synthesis and application to the paleoenvironmental evolution of northwestern Italy. In *Stratigraphic Analysis of Layered Deposits*. (ed.^eds. O. Elitok), pp. 123–160. Rijeka: InTech.
66 . Wagner, HM, Riney, BO, Deméré, TA, Prothero, DR. 2001 Magnetic stratigraphy and land mammal biochronology of the nonmarine facies of the Pliocene San Diego Formation, San Diego County, California. *Pacific Section SEPM Book*. **91**, 359–368.
67 . Hanna, GD, McLellan, M. 1924 A new species of whale from the type locality of the Monterey Group. *Proc Calif Acad Sci*. **13**, 237–241.
68 . Barron, JA. 1976 Marine diatom and silicoflagellate biostratigraphy of the type Delmontian Stage and the type *Bolivina obliqua* Zone, California. *Journal of Research, United States Geological Survey*. **4**, 339-351.
69 . Obradovich, JD, Naeser, CW. 1981 Geochronology bearing on the age of the Monterey Formation and siliceous rocks in California. *SEPM Spec Publ*. **15**, 87-95.
70 . Beyer, LA, McCulloh, TH, Denison, RE, Morin, RW, Enrico, RJ, Barron, JA, Fleck, RJ. 2009 Post-Miocene right separation on the San Gabriel and Vasquez Creek Faults, with supporting chronostratigraphy, western San Gabriel Mountains, California. *US Geol Surv Prof Pap*. **1759**, 1-44.
71 . Cody, RD, Levy, RH, Harwood, DM, Sadler, PM. 2008 Thinking outside the zone: high-resolution quantitative diatom biochronology for the Antarctic Neogene. *Palaeogeogr Palaeoclimatol Palaeoecol*. **260**, 92-121. (http://dx.doi.org/10.1016/j.palaeo.2007.08.020)
72 . Pilleri, G. 1989 *Balaenoptera siberi*, ein neuer spätmiozäner Bartenwal aus der Pisco-Formation Perus. In *Beiträge zur Paläontologie der Cetaceen Perus*. (ed.^eds. G. Pilleri), pp. 63–84. Bern: Hirnanatomisches Institut Ostermundingen.
73 . Pilleri, G. 1990 Paratypus von *Balaenoptera siberi* (Cetacea: Mysticeti) aus der Pisco Formation Perus. In *Beiträge zur Paläontologie der Cetaceen und Pinnipedier der Pisco Formation Perus II*. (ed.^eds. G. Pilleri), pp. 205–215. Bern: Hirnanatomisches Institut Ostermundingen.

74 . Bouetel, V, de Muizon, C. 2006 The anatomy and relationships of *Piscobalaena nana* (Cetacea, Mysticeti), a Cetotheriidae s.s. from the early Pliocene of Peru. *Geodiversitas*. **28**, 319-395.
75 . de Muizon, C, Devries, TJ. 1985 Geology and paleontology of late Cenozoic marine deposits in the Sacaco area (Peru). *Geol Rundsch*. **74**, 547-563. (10.1007/bf01821211)
76 . Trevisan, L. 1941 Una nuova specie di *Balaenula* Pliocenica. *Palaeontogr Ital*. **40**, 1-13.
77 . Violanti, D. 2005 Pliocene foraminifera of Piedmont (north-western Italy): a synthesis of recent studies. *Annali dell'Università degli Studi di Ferrara Museologia Scientifica e Naturalistica*. **Volume speciale 2005**, 75–88.
78 . Kimura, T. 2009 Review of the fossil balaenids from Japan with a re-description of *Eubalaena shinshuensis* (Mammalia, Cetacea, Mysticeti). *Quad Mus Stor Nat Livorno*. **22**, 3-21.
79 . Excavation Research Group for the Fukagawa Whale Fossil. Excavation Research Group for the Fukagawa Whale Fossil 1982 *Research report on the Pliocene Balaenula (Suborder Mysticoceti [sic]) collected from Fukagawa City, Hokkaido* Fukagawa City: City Board of Education.
80 . Wada, N, al., e. 1985 Stratigraphy of the Pliocene Fukagawa Group in the northern part of Fukagawa City, Central Hokkaido. *Earth Sci (Chikyu Kagaku)*. **39**, 243–257.
81 . Maeda, T. 1984 Tuff. In *Research Report of Takikawa Sea Cow*. (ed.^eds. R. G. f. T. S. Cow), pp. 43–53. Takikawa: Takikawa City Board of Education.
82 . Ganzawa, Y. 1984 Fission Track age determining. In *Research Report of Takikawa Sea Cow*. (ed.^eds. R. G. f. T. S. Cow), pp. 59–63. Takikawa: Takikawa City Board of Education.
83 . Nakashima, R, Watanabe, M. 2000 First occurrence age of *Fortipecten takahashii* (Yokoyama) (Bivalvia: Pectinidae) from the lower part of the upper Miocene Horokaoshirarika Formation in Numata-cho, central Hokkaido. *J Geol Soc Jpn*. **106**, 578–581.
84 . Yanagisawa, Y, Akiba, F. 1998 Refined Neogene diatom biostratigraphy for the northwest Pacific around Japan, with an introduction of code numbers for selected diatom biohorizons. *J Geol Soc Jpn*. **104**, 395-414.
85 . Gol'din, P, Startsev, D. 2014 *Brandtocetus*, a new genus of baleen whales (Cetacea, Cetotheriidae) from the Late Miocene of Crimea, Ukraine. *J Vertebr Paleontol*. **34**, 419-433. (10.1080/02724634.2013.799482)
86 . Harzhauser, M, Piller, WE. 2004 Integrated stratigraphy of the Sarmatian (Upper Middle Miocene) in the western Central Paratethys. *Stratigraphy*. **1**, 65-86.
87 . Piller, WE, Harzhauser, M, Mandic, O. 2007 Miocene Central Paratethys stratigraphy – current status and future directions. *Stratigraphy*. **4**, 151-168.

88 . Vangengeim, EA, Tesakov, AS. 2008 Late Sarmatian mammal localities of the Eastern Paratethys: Stratigraphic position, magnetochronology, and correlation with the European continental scale. *Stratigr Geol Correl*. **16**, 92-103. (10.1007/s11506-008-1007-x)

89 . Radionova, E, Golovina, L, Filippova, N, Trubikhin, V, Popov, S, Goncharova, I, Vernigorova, Y, Pinchuk, T. 2012 Middle-Upper Miocene stratigraphy of the Taman Peninsula, Eastern Paratethys. *Centr Eur J Geosci*. **4**, 188-204. (10.2478/s13533-011-0065-8)

90 . Cope, ED. 1896 Sixth contribution to the knowledge of the Miocene fauna of North Carolina. *Proc Am Philos Soc*. **35**, 139–146.

91 . Kellogg, R. 1941 On the cetotheres figured by Vandelli. *Bol Mus Lab Miner Geol Fac Cienc Univ Lisb*. **7-8**, 1-12.

92 . Mocho, P, Póvoas, L. 2010 Contribucao para revisao sistematica de um cranio de *Cephalotropis* Cope, 1896 (Cetacea: Cetotheriidae) do Miocenico superior (Tortoniano inferior) da Adica (Sesimbra, Portugal). *Publ Semin Paleontol Zaragoza*. **9**, 180-183.

93 . Kellogg, R. 1931 Pelagic mammals of the Temblor Formation of the Kern River region, California. *Proc Calif Acad Sci*. **19**, 217-397.

94 . Verteuil, Ld, Norris, G. 1996 Miocene dinoflagellate stratigraphy and systematics of Maryland and Virginia. *Micropaleontol*. **42 (Suppl.)**, 1-172.

95 . Cotter, JB. 1956 O Miocénico marinho de Lisboa. *Com Serv Geol Portugal*. **36**, 1-170.

96 . Estevens, M, Antunes, MT. 2004 Fragmentary remains of odontocetes (Cetacea, Mammalia) from the Miocene of the Lower Tagus Basin (Portugal). *Rev Esp Paleontol*. **19**, 93-108.

97 . Pais, J, Legoinha, P, Estevens, M. 2008 Património paleontológico do Concelho de Almada. In *A Terra: Conflitos e Ordem. Livro homenagem Prof. António Ferreira Soares*. (ed.^eds. P. M. Callapez, R. B. Rocha, J. F. Marques, L. Cunha, P. Dinis), pp. 143-158. Coimbra: Museu Mineralógico e Geológico da Universidade de Coimbra.

98 . Cope, ED. 1895 Fourth contribution to the marine fauna of the Miocene period of the United States. *Proc Am Philos Soc*. **34**, 135-155.

99 . Brandt, JF. 1873 Untersuchungen über die fossilen und subfossilen Cetaceen Europa's. *Mém Acad Imp Sci St Pétersbourg*. **20**, 1-371. (10.5962/bhl.title.39524)

100 . Gol'din, P. 2014 The anatomy of the Late Miocene baleen whale *Cetotherium riabinini* from Ukraine. *Acta Palaeontol Pol*. **59**, 795-814. (http://dx.doi.org/10.4202/app.2012.0107)

101 . Barnes, LG, Sanders, AE. 1996 The transition from archaeocetes to mysticetes: Late Oligocene toothed mysticetes from near Charleston, South Carolina. *Paleontol Soc Spec Publ*. **8**, 24.

102 . Fitzgerald, EMG. 2010 The morphology and systematics of *Mammalodon colliveri* (Cetacea: Mysticeti), a toothed mysticete from the Oligocene of Australia. *Zool J Linn Soc Lond*. **158**, 367-476. (10.1111/j.1096-3642.2009.00572.x)

103 . Hazel, JE, Bybell, LM, Christopher, RA, Fredericksen, NO, May, FE, McLean, DM, Poore, RZ, Smith, CC, Sohl, NF, Valentine, PC*, et al.* 1977 Biostratigraphy of the deep corehole (Clubhouse Crossroads corehole 1) near Charleston, South Carolina. *US Geol Surv Prof Pap*. **1028-F**, 71-89.

104 . Köthe, A. 2012 A revised Cenozoic dinoflagellate cyst and calcareous nannoplankton zonation for the German sector of the southeastern North Sea Basin. *Newsl Stratigr*. **45**, 189-220. (10.1127/0078-0421/2012/0021)

105 . Weems, RE, Harris, WB. Major change in depositional style and paleoclimate in the southeastern United States at or very near the Oligocene-Miocene boundary. *33rd International Geological Congress*. Oslo 2008:Abstract HPS-08.

106 . Prothero, DR, Streig, A, Burns, C. 2001 Magnetic stratigraphy and tectonic rotation of the Upper Oligocene Pysht Formation, Clallam County, Washington. *Pacific Section SEPM Book*. **91**, 224-233.

107 . Russell, LS. 1968 A new cetacean from the Oligocene Sooke Formation of Vancouver Island, British Columbia. *Can J Earth Sci*. **5**, 929-933. (10.1139/e68-089)

108 . Prothero, DR, Draus, E, Cockburn, TC, Nesbitt, EA. 2008 Paleomagnetism and counterclockwise tectonic rotation of the Upper Oligocene Sooke Formation, southern Vancouver Island, British Columbia. *Can J Earth Sci*. **45**, 499-507. (10.1139/e08-012)

109 . Yoshida, K, Kimura, T, Hasegawa, Y. 2003 New cetothere (Cetacea: Mysticeti) from the Miocene Chichibumachi Group, Japan. *Bull Saitama Mus Nat Hist*. **20-21**, 1-10.

110 . Yagi, N, Ishigaki, T. 1993 Miocene smaller foraminiferal assemblages from the Chichibumachi Group in the Chichibu Basin, Saitama Prefecture, Japan. *Mem Fac Lib Arts Educ Yamanashi Univ II, Math Nat Sci*. **44**, 55-60.

111 . Kimura, T, Hasegawa, Y. 2004 An outline of the Miocene cetotheres of Japan. *Bull Gunma Mus Nat Hist*. **8**, 79-88.

112 . Kellogg, R. 1968 Fossil marine mammals from the Miocene Calvert Formation of Maryland and Virginia, part 6: a hitherto unrecognized Calvert cetothere. *US Nat Mus Bull*. **247**, 133-161.

113 . Otsuka, H, Ota, Y. 2008 Cetotheres from the early Middle Miocene Bihoku Group in Shobara District, Hiroshima Prefecture, West Japan. *Misc Rep Hiwa Mus Nat Hist*. **49**, 1-66.

114 . Yamamoto, Y, Sato, T. 1999 Miocene calcareous nannofossils of the Bihoku Group in the Shobara Area, Hiroshima Prefecture, Southwest Japan. *J Geosci Osaka City Univ*. **42**, 55-67.

115 . Yamamoto, Y. 1999 Lithofacies and calcareous nannofossils of the Miocene Bihoku Group in the Saijogawa area, Shobara, Hiroshima, Japan. *Earth Sci (Chikyu Kagaku)*. **53**, 202-216.
116 . Tsuchi, R. 1990 Neogene events in Japan and the Pacific. *Palaeogeogr Palaeoclimatol Palaeoecol*. **77**, 355-365. (http://dx.doi.org/10.1016/0031-0182(90)90186-B)
117 . Watanabe, M, Miyake, M, Nozaki, S, Yamamoto, Y, Takemura, A, Nishimura, T. 1999 Miocene diatom fossils from the Bihoku Group in Koyamaichi area and the Katsuta Group in Tsuyama area, Okayama Prefecture, southwest Japan. *J Geol Soc Jpn*. **105**, 116-121. (10.5575/geosoc.105.116)
118 . Watanabe, M, Yanagisawa, Y. 2005 Refined Early to Middle Miocene diatom biochronology for the middle- to high-latitude North Pacific. *Isl Arc*. **14**, 91-101. (10.1111/j.1440-1738.2005.00460.x)
119 . Sanders, AE, Barnes, LG. 2002 Paleontology of the Late Oligocene Ashley and Chandler Bridge Formations of South Carolina, 3: Eomysticetidae, a new family of primitive mysticetes (Mammalia, Cetacea). *Smithson Contrib Paleobiol*. **93**, 313-356.
120 . Sanders, AE, Weems, RE, Lemon, EM, Jr. 1982 Chandler Bridge Formation – a new Oligocene stratigraphic unit in the lower coastal plain of South Carolina. *US Geol Surv Bull*. **1529-H**, H105-H124.
121 . Katuna, MP, Geisler, JH, Colquhoun, DJ. 1997 Stratigraphic correlation of oligocene marginal marine and fluvial deposits across the middle and lower coastal plain, South Carolina. *Sediment Geol*. **108**, 181-194. (http://dx.doi.org/10.1016/S0037-0738(96)00053-X)
122 . Plisnier-Ladame, F, Quinet, GE. 1969 *Balaena belgica* Abel 1938, cetace du merxemien d'Anvers. *Bull Inst R Sci Nat Belg Biol*. **45**, 1-6.
123 . Motoyama, I, Nagamori, H. 2006 Radiolarians from the Pliocene of the Hokushin district, Nagano Prefecture, Japan. *J Geol Soc Jpn*. **112**, 541-548. (10.5575/geosoc.112.541)
124 . Niitsuma, S, Niitsuma, N, Saito, K. 2003 Evolution of the Komiji Syncline in the North Fossa Magna, central Japan: Paleomagnetic and K–Ar age insights. *Isl Arc*. **12**, 310-323. (10.1046/j.1440-1738.2003.00397.x)
125 . Whitmore, FC, Jr., Kaltenbach, JA. 2008 Neogene Cetacea of the Lee Creek Phosphate Mine, North Carolina. *Va Mus Nat Hist Spec Publ*. **14**, 181-269.
126 . Whitmore, FC, Jr, Barnes, LG. 2008 The Herpetocetinae, a new subfamily of extinct baleen whales (Mammalia, Cetacea, Cetotheriidae). *Va Mus Nat Hist Spec Publ*. **14**, 141-180.

127 . Marx, F, Buono, M, Fordyce, RE, Boessenecker, RW. 2013 Juvenile morphology: a clue to the origins of the most mysterious of mysticetes? *Naturwissenschaften*. **100**, 257-261. (10.1007/s00114-013-1012-y)
128 . Madrid, VM, Stuart, RM, Verosub, KL. 1986 Magnetostratigraphy of the late Neogene purisima formation, Santa Cruz County, California. *Earth Planet Sci Lett*. **79**, 431-440. (http://dx.doi.org/10.1016/0012-821X(86)90198-6)
129 . Boessenecker, RW, Geisler, JH. 2008 New material of the bizarre whale *Herpetocetus bramblei* from the latest Miocene Purisima Formation of Central California. *J Vertebr Paleontol*. **28 (Suppl. to 3)**, 54A.
130 . Powell, CL, II, Barron, JA, Sarna-Wojcicki, AM, Clark, JC, Perry, FA, Brabb, EE, Fleck, RJ. 2007 Age, stratigraphy, and correlations of the late Neogene Purisima Formation, Central California Coast Ranges. *US Geol Surv Prof Pap*. **1740**, 1-32.
131 . Kimura, T, Ozawa, T. 2002 A new cetothere (Cetacea: Mysticeti) from the early Miocene of Japan. *J Vertebr Paleontol*. **22**, 684-702. (10.1671/0272-4634(2002)022[0684:anccmf]2.0.co;2)
132 . Yoshida, F. 1987 Planktonic foraminifera from the Miocene Awa Group, Mie Prefecture, central Japan. *Bull Geol Surv Jpn*. **38**, 473-483.
133 . Yoshida, F. 1991 Planktonic foraminifera from the Ichishi, Fujiwara, and Morozaki Groups in the eastern Setouchi geologic province, central Japan. *Bull Mizunami Fossil Mus*. **18**, 19-32.
134 . Itoigawa, J, Shibata, H. 1992 Miocene paleogeography of the Setouchi Geologic Province, Japan, a revision. *Bull Mizunami Fossil Mus*. **19**, 1-12.
135 . Kobayashi, T. 1989 Geology and uranium mineralization in the eastern part of the Kani basin, Gifu, Central Japan. *Mining Geology*. **39**, 79-94. (10.11456/shigenchishitsu1951.39.214_79)
136 . Fitzgerald, EMG. 2006 A bizarre new toothed mysticete (Cetacea) from Australia and the early evolution of baleen whales. *Proc R Soc B*. **273**, 2955-2963. (10.1098/rspb.2006.3664)
137 . Fitzgerald, EMG. 2012 Archaeocete-like jaws in a baleen whale. *Biol Lett*. **8**, 94-96. (10.1098/rsbl.2011.0690)
138 . McLaren, S, Wallace, MW, Gallagher, SJ, Dickinson, JA, McAllister, A. 2009 Age constraints on Oligocene sedimentation in the Torquay Basin, southeastern Australia. *Aust J Earth Sci*. **56**, 595-604. (10.1080/08120090902806347)
139 . Marx, F. 2011 The more the merrier? A large cladistic analysis of mysticetes, and comments on the transition from teeth to baleen. *J Mamm Evol*. **18**, 77-100. (10.1007/s10914-010-9148-4)

140 . Kimura, T, Hasegawa, Y. 2010 A new baleen whale (Mysticeti: Cetotheriidae) from the earliest late Miocene of Japan and a reconsideration of the phylogeny of cetotheres. *J Vertebr Paleontol*. **30**, 577-591. (10.1080/02724631003621912)

141 . Odin, GS, Takahashi, M, Cosca, M. 1995 40Ar/39Ar geochronology of biostratigraphically controlled Miocene tuffs from central Japan: Comparison with Italy and age of the Serravallian-Tortonian boundary. *Chem Geol*. **125**, 105-121. (http://dx.doi.org/10.1016/0009-2541(95)00075-W)

142 . Takahashi, M, Hayashi, H. 2004 Geology and integrated chronostratigraphy of the Miocene marine sequence in the Tomioka area, Gunma Prefecture, central Japan. *The Journal of the Geological Society of Japan*. **110**, 175-194. (10.5575/geosoc.110.175)

143 . Tarasenko, KK, Lopatin, AV. 2012 New baleen whale genera (Cetacea, Mammalia) from the Miocene of the northern Caucasus and Ciscaucasia: 1. *Kurdalagonus* gen. nov. from the middle-late Sarmatian of Adygea. *Paleontol J*. **46**, 531-542. (10.1134/s0031030112050115)

144 . Beluzhenko, EV, Volkodav, IG, Derkacheva, MG, Korsakov, SG, Sokolov, VV, Chernykh, VI. 2007 *Oligocene and Neogene Deposits of Belaya River Valley (Adygeya)*. Maikop: Izdatelstvo Adygeyskogo Gosudarstvennogo Universiteta.

145 . Fordyce, RE. 2003 Early crown group Cetacea in the Southern Ocean: the toothed archaic mysticete *Llanocetus*. *J Vertebr Paleontol*. **23**, 50A.

146 . Mitchell, ED. 1989 A new cetacean from the Late Eocene La Meseta Formation, Seymour Island, Antarctic Peninsula. *Can J Fish Aquat Sci*. **46**, 2219-2235. (10.1139/f89-273)

147 . Fordyce, RE. 2003 Cetacean evolution and Eocene-Oligocene oceans revisited. In *From Greenhouse to Icehouse - The Marine Eocene-Oligocene Transition*. (ed.^eds. D. R. Prothero, L. C. Ivany, E. A. Nesbitt), pp. New York: Columbia University Press.

148 . Ivany, LC, Lohmann, KC, Hasiuk, F, Blake, DB, Glass, A, Aronson, RB, Moody, RM. 2008 Eocene climate record of a high southern latitude continental shelf: Seymour Island, Antarctica. *Geol Soc Am Bull*. **120**, 659-678. (10.1130/b26269.1)

149 . Dingle, RV, Lavelle, M. 1998 Antarctic Peninsular cryosphere: Early Oligocene (c. 30 Ma) initiation and a revised glacial chronology. *Journal of the Geological Society*. **155**, 433-437. (10.1144/gsjgs.155.3.0433)

150 . Li, Q, Davies, PJ, McGowran, B. 1999 Foraminiferal sequence biostratigraphy of the Oligo-Miocene Janjukian strata from Torquay, southeastern Australia*. *Aust J Earth Sci*. **46**, 261-273. (10.1046/j.1440-0952.1999.00696.x)

151 . Kelly, JC, Webb, JA, Maas, R. 2001 Isotopic constraints on the genesis and age of autochthonous glaucony in the Oligo-Miocene Torquay Group, south-eastern Australia. *Sedimentology*. **48**, 325-338. (10.1046/j.1365-3091.2001.00365.x)

152 . Holdgate, GR, Gallagher, SJ. 2003 Tertiary: a period of transition to marine basin environments. *Geol Soc Aust Spec Publ*. **23**, 289-335.
153 . Fordyce, RE. 2005 New specimen of archaic baleen whale Mauicetus parki (Late Oligocene, New Zealand) elucidates early crown-Mysticeti. *J Vertebr Paleontol*. **25 (Suppl. to 3)**, 58A.
154 . Benham, WB. 1937 On Lophocephalus, a new genus of zeuglodont Cetacea *Trans Proc Roy Soc N Z*. **67**, 1-7.
155 . Dathe, F. 1983 *Megaptera hubachi* n. sp., ein fossiler Bartenwal aus marinen Sandsteinschichten des tieferen Pliozaens Chiles. *Z Geol Wiss*. **11**, 813-848.
156 . Le Roux, JP, Olivares, DM, Nielsen, SN, Smith, ND, Middleton, H, Fenner, J, Ishman, SE. 2006 Bay sedimentation as controlled by regional crustal behaviour, local tectonics and eustatic sea-level changes: Coquimbo Formation (Miocene–Pliocene), Bay of Tongoy, central Chile. *Sediment Geol*. **184**, 133-153. (http://dx.doi.org/10.1016/j.sedgeo.2005.09.023)
157 . Herm, D. 1969 Marines Pliozän und Pleistozän in Nord- und Mittel-Chile unter besonderer Berücksichtigung der Entwicklung der Mollusken-Faunen. *Zitteliana*. **2**, 1-159.
158 . Salinas, P. 1988 Hallazgo de cetáceos fósiles (Mysteceti, Balaenopteridae) en la ciudad de Coquimbo, Chile. *Revista Geológica de Chile*. **15**, 89-94.
159 . Kellogg, R. 1922 Description of the skull of *Megaptera miocaena*, a fossil humpback whale from the Miocene diatomaceous earth of Lompoc, California. *Proc US Nat Mus*. **61**, 1-18.
160 . Chang, AS, Grimm, KA. 1999 Speckled beds: distinctive gravity-flow deposits in finely laminated diatomaceous sediments, Miocene Monterey Formation, California. *J Sediment Res*. **69**, 122-134. (10.2110/jsr.69.122)
161 . Motoyama, I, Matuyama, T. 1998 Neogene diatom and radiolarian biochronology for the middle-to-high latitudes of the Northwest pacific region: Calibration to the Cande and Kent's geomagnetic polarity time scales (CK 92 and CK 95). *J Geol Soc Jpn*. **104**, 171-183.
162 . Akiba, F. 1986 Middle Miocene to Quaternary diatom biostratigraphy in the Nankai Trough and Japan Trench, and modified lower Miocene through Quaternary diatom zones for middle-to-high latitudes of the North Pacific. *Initial Rep Deep Sea Drill Proj*. **87**, 393-481.
163 . Kellogg, R. 1968 Fossil marine mammals from the Miocene Calvert Formation of Maryland and Virginia, part 5: Miocene Calvert mysticetes described by Cope. *US Nat Mus Bull*. **247**, 103-132.

164 . Case, EC. 1904 Mammalia. In *Miocene*. (ed.^eds. W. B. Clark), pp. 3-57. Baltimore: The John Hopkins Press.
165 . Sanders, AE, Barnes, LG. 2002 Paleontology of the Late Oligocene Ashley and Chandler Bridge Formations of South Carolina, 2: *Micromysticetus rothauseni*, a primitive cetotheriid mysticete (Mammalia: Cetacea). *Smithson Contrib Paleobiol*. **93**, 271-293.
166 . Bisconti, M. 2012 Comparative osteology and phylogenetic relationships of *Miocaperea pulchra*, the first fossil pygmy right whale genus and species (Cetacea, Mysticeti, Neobalaenidae). *Zool J Linn Soc Lond*. **166**, 876-911. (10.1111/j.1096-3642.2012.00862.x)
167 . Ehret, DJ, Macfadden, BJ, Jones, DS, Devries, TJ, Foster, DA, Salas-Gismondi, R. 2012 Origin of the white shark *Carcharodon* (Lamniformes: Lamnidae) based on recalibration of the Upper Neogene Pisco Formation of Peru. *Palaeontology*. **55**, 1139-1153. (10.1111/j.1475-4983.2012.01201.x)
168 . Sawamura, H, Ichishima, H, Ito, H, Ishikawa, H. 2006 Features implying the beginning of baleen growth in aetiocetids. *J Vertebr Paleontol*. **26 (Suppl. to 3)**, 120A.
169 . Kellogg, R. 1929 A new cetothere from southern California. *Univ Calif Publ Geol Sci*. **18**, 449-457.
170 . Domning, DD. 1978 Sirenian evolution in the North Pacific Ocean. *Univ Calif Publ Geol Sci*. **118**, 1-176.
171 . Fordyce, RE, Marx, FG. 2013 The pygmy right whale *Caperea marginata*: the last of the cetotheres. *Proc R Soc B*. **280**, (10.1098/rspb.2012.2645)
172 . Barron, JA, Isaacs, CM. 2001 Updated chronostratigraphic framework for the California Miocene. In *The Monterey Formation. From Rocks to Molecules*. (ed.^eds. C. M. Isaacs, J. Rullkötter), pp. 393-395. New York: Columbia University Press, New York.
173 . Repenning, CA, Tedford, RH. 1977 Otarioid seals of the Neogene. *US Geol Surv Prof Pap*. **992**, 1-93.
174 . Bearlin, RK. 1987 Neogene Mysticeti [PhD Thesis]. Dunedin: University of Otago.
175 . Beu, AG. 1995 Pliocene limestones and their scallops: lithostratigraphy, pectinid biostratigraphy and paleogeography of eastern North Island and late Neogene limestone. *Inst Geol Nucl Sci Monogr*. **10**, 1-243.
176 . Rivin, MA. 2010 Early Miocene cetacean diversity in the Vaqueros Formation, Laguna Canyon, Orange County, California [MSc Thesis]. Fullerton: California State University Fullerton.
177 . Calvano, G, Prothero, DR, Ludtke, J, Lander, EB. 2008 Magnetic stratigraphy of the Eocene to Miocene Sespe and Vaqueros formations, Los Angeles and Orange counties, California. *Nat Hist Mus Los Angel Cty Sci Ser*. **41**, 43-61.

178 . Fordyce, RE, Marx, FG. 2011 Toothed mysticetes and ecological structuring of Oligocene whales and dolphins from New Zealand. *Geol Surv W A Rec*. **2011/9**, 33.
179 . Nelson, CS, Lee, D, Maxwell, P, Maas, R, Kamp, PJJ, Cooke, S. 2004 Strontium isotope dating of the New Zealand Oligocene. *N Z J Geol Geophys*. **47**, 719-730. (10.1080/00288306.2004.9515085)
180 . Boessenecker, RW, Fordyce, RE. 2014 Trace fossil evidence of predation upon bone-eating worms on a baleen whale skeleton from the Oligocene of New Zealand. *Lethaia*. published online. (10.1111/let.12108)
181 . Fordyce, RE. 2002 Oligocene origins of skim-feeding right whales: a small archaic balaenid from New Zealand. . *J Vertebr Paleontol*. **22**, 54A.
182 . Morgans, HEG, Edwards, AR, Scott, GH, Graham, IJ, Kamp, PJJ, Mumme, TC, Wilson, GJ, Wilson, GS. 1999 Integrated stratigraphy of the Waitakian-Otaian Stage boundary stratotype, Early Miocene, New Zealand. *N Z J Geol Geophys*. **42**, 581-614.
183 . Keyes, IW. 1973 Early Oligocene squalodont cetacean from Oamaru, New Zealand. *N Z J Mar Freshwater Res*. **7**, 381-390.
184 . Edwards, AR. 1991 The Oamaru Diatomite. *N Z Geol Surv Paleontol Bull*. **64**, 1-260.
185 . Zeigler, CV, Chan, GL, Barnes, LG. 1997 A new Late Miocene balaenopterid whale (Cetacea: Mysticeti), *Parabalaenoptera baulinensis*, (new genus and species) from the Santa Cruz Mudstone, Point Reyes Peninsula, California. *Proc Calif Acad Sci*. **50**, 115-138.
186 . Kellogg, R. 1924 Description of a new genus and species of whalebone whale from the Calvert Cliffs, Maryland. *Proc US Nat Mus*. **63**, 1-14.
187 . Kellogg, R. 1968 Fossil marine mammals from the Miocene Calvert Formation of Maryland and Virginia, part 8: supplement to the description of *Parietobalaena palmeri US Nat Mus Bull*. **247**, 175-197.
188 . Kimura, T, Sakamoto, O, Hasegawa, Y. 1998 A cetothere from the Miocene Chichibumachi Group, Saitama Prefecture, Japan. *Bull Saitama Mus Nat Hist*. **16**, 1-13.
189 . Kimura, T, Hasegawa, Y, Ohzawa, H, Yamaoka, T, Ueda, T, Kiyoshi, T, Furukawa, Y, Sugihara, M. 2011 An additional fossil mysticete from the middle Miocene Bihoku Group, Hiroshima, Japan. *Bull Gunma Mus Nat Hist*. **15**, 81-92.
190 . Kimura, T, Hasegawa, Y, Ohzawa, H, Ueda, T, Yamaoka, T. 2010 A fossil mysticete from the middle Miocene Bihoku Group, Hiroshima, Japan. *Bull Gunma Mus Nat Hist*. **14**, 29-36.
191 . Kellogg, R. 1965 Fossil marine mammals from the Miocene Calvert Formation of Maryland and Virginia, part 1: a new whalebone whale from the Miocene Calvert Formation. *US Nat Mus Bull*. **247**, 1-45.

192 . Steeman, EM. 2007 Cladistic analysis and a revised classification of fossil and recent mysticetes. *Zool J Linn Soc Lond*. **150**, 875-894.
193 . Prothero, DR, Sanchez, F, Denke, LL. 2008 Magnetic stratigraphy of the Early to Middle Miocene Olcese Sand and Round Mountain Silt, Kern County, California. *Bull N M Mus Nat Hist Sci*. **44**, 357-364.
194 . Czyżewska, T, Ryziewicz, Z. 1976 *Pinocetus polonicus* gen. n. sp. n. (Cetacea) from the Miocene limestones of Pinczów, Poland. *Acta Palaeontol Pol*. **21**, 259-298.
195 . Studencki, W. 1988 Facies and sedimentary environment of the Pinczow limestones (Middle Miocene; Holy Cross Mountains, central Poland). *Facies*. **18**, 1-25. (10.1007/bf02536793)
196 . Stachacz, M. 2007 Remarks on age of the Middle Miocene deposits in the Szydłów area (southern margin of the Holy Cross Mountains). *Prz Geol*. **55**, 168-174.
197 . Studencka, B. 1999 Remarks on Miocene bivalve zonation in the Polish part of the Carpathian Foredeep. *Geol Q*. **43**, 467-477.
198 . Hohenegger, J, Wagreich, M. 2012 Time calibration of sedimentary sections based on insolation cycles using combined cross-correlation: dating the gone Badenian stratotype (Middle Miocene, Paratethys, Vienna Basin, Austria) as an example. *Int J Earth Sci*. **101**, 339-349. (10.1007/s00531-011-0658-y)
199 . Pilleri, G. 1993 New find of *Piscobalaena nana* (Cetacea: Mysticeti) from the Pisco Formation of Peru. *Investig Cetacea*. **24**, 289-313.
200 . Bisconti, M. 2010 A new balaenopterid whale from the late Miocene of the Stirone River, northern Italy (Mammalia, Cetacea, Mysticeti). *J Vertebr Paleontol*. **30**, 943-958. (Pii 922421979
10.1080/02724631003762922)
201 . Artoni, A, Papani, G, Rizzini, F, Calderoni, M, Bernini, M, Argnani, A, Roveri, M, Rossi, M, Rogledi, S, Gennari, R. 2004 The Salsomaggiore structure (Northwestern Apennine foothills, Italy): a Messinian mountain front shaped by mass-wasting products. *GeoActa*. **3**, 107-127.
202 . Kellogg, R. 1969 Cetothere skeletons from the Miocene Choptank Formation of Maryland and Virginia. *US Nat Mus Bull*. **294**, 1-40.
203 . Bisconti, M. 2006 *Titanocetus*, a new baleen whale from the Middle Miocene of northern Italy (Mammalia, Cetacea, Mysticeti). *J Vertebr Paleontol*. **26**, 344-354. (10.1671/0272-4634(2006)26[344:tanbwf]2.0.co;2)
204 . Amorosi, A. 1992 Correlazioni stratigrafiche e sequenze deposizionali nel Miocene epiligure delle Formazioni di Bismantova, San Marino e Fumaiolo (Appennino settentrionale). *G Geol*. **54**, 95-105.

205 . Bortolotti, V, Mannori, G, Principi, G, Sani, F. 2008 *Note illustrative della carta geologica d'Italiana alla scala 1:50.000 – foglio 278 Pieve Santo Stefano*. Firenze: Agenzia per la protezione dell'ambiente e per i servizi tecnici, Servizio Geologico d'Italia.
206 . Mazzei, R, Margiotta, S, Foresi, LM, Riforgiato, F, Salvatorini, G. 2009 Biostratigraphy and chronostratigraphy of the Miocene Pietra Leccese in the type area of Lecce (Apulia, southern Italy). *Boll Soc Paleontol Ital*. **48**, 129-145.
207 . Steeman, ME. 2009 A new baleen whale from the Late Miocene of Denmark and early mysticete hearing. *Palaeontology*. **52**, 1169-1190. (10.1111/j.1475-4983.2009.00893.x)
208 . Piasecki, S. 2005 Dinoflagellate cysts of the middle - upper Miocene Gram Formation, Denmark. *Palaeontos*. **7**, 29-45.
209 . Hansen, J, Hansen, T. 2003 Stratigraphy and sea-level fluctuations in the Upper Miocene Gram Formation, south-western Denmark. *Bull Geol Soc Den*. **50**, 157-169.
210 . Dybkjær, K, Piasecki, S. 2010 Neogene dinocyst zonation for the eastern North Sea Basin, Denmark. *Rev Palaeobot Palynol*. **161**, 1-29. (http://dx.doi.org/10.1016/j.revpalbo.2010.02.005)
211 . Goedert, JL, Crowley, BJ, Barnes, LG. 2001 Very primitive, Early Oligocene mysticetes (Cetacea: Mysticeti) from the eastern North Pacific. *J Vertebr Paleontol*. **21 (Suppl. to 3)**, 55A.
212 . Prothero, DR, Draus, E, Burns, C. 2009 Magnetostratigraphy and tectonic rotation of the Eocene-Oligocene Makah and Hoko River formations, Northwest Washington, USA. *Int J Geophys*. **2009**, 1-15. (10.1155/2009/930612)
213 . Okazaki, Y. 2012 A new mysticete from the upper Oligocene Ashiya Group, Kyushu, Japan and its significance to mysticete evolution. *Bull Kitakyushu Mus Nat Hist Hum Hist Ser A Nat Hist*. **10**, 129-152.
214 . Ozaki, M, Hamasaki, S. 1991 Fission track ages of the Paleogene strata in the northern part of Fukuoka Prefecture, Southwest Japan. *J Geol Soc Jpn*. **97**, 251-254.
215 . Tsuchi, R, Shuto, T, Ibaraki, M. 1987 Geologic ages of the Ashiya Group, North Kyushu from viewpoint of planktonic foraminifera. *Rep Fac Sci Shizuoka Univ*. **21**, 109-119.
216 . Ibaraki, M. 1994 Ages and paleoenvironments of the Tertiary in north-western Kyushu on the basis of planktonic foraminifers. *Gekkan Chikyu*. **16**, 150-153.
217 . Ishikawa, N. 1990 Differential rotation of the western part of Southwest Japan: paleomagnetic study in the northern part of the Kyushu Island. *Rock Magn Paleogeoph*. **17**, 73-79.
218 . Morgans, HEG. 2004 Palaeogene. *Inst Geol Nucl Sci Monogr*. **22**, 125-161.

219 . Fordyce, RE. 2006 An unexpected diversity of basal baleen whales: Late Oligocene Eomysticetidae from New Zealand. *J Vertebr Paleontol*. **26 (Suppl. to 3)**, 62A.
220 . Ichishima, H. 2002 What were archaic mysticetes like? An example of some latest Oligocene to earliest Miocene New Zealand whales. *Geol Soc N Z Misc Publ*. **114A**, 24-25.

Morphological characters

Cranium

1. Length of rostral portion of maxilla anterior to antorbital notch: less than bizygomatic width (0); equal to or greater than bizygomatic width (1); more than one and a half times the bizygomatic width (2). ORDERED

2. Portion of rostrum anterior to nasals in lateral view: below the level of the supraoccipital (0); raised to or above the supraoccipital (1).

3. Lateral edge of maxilla in cross section: forms an angle of more than 45 degrees (0); lateral edge is flattened with an angle of less than 45 degrees (1).

4. Lateral border of maxilla anterior to antorbital notch (or homologous point on rostrum) in dorsal view: concave (0); straight or slightly convex (1); broadly convex (2). ORDERED

5. Transverse width of maxilla at midpoint: distinctly less than that of the premaxilla (0); roughly equal to or up to twice the width of the premaxilla (1); more than twice the width of the premaxilla (2). ORDERED

6. Premaxilla in dorsal view: widens at anterior end (0); portion anterior to nasal opening narrows or remains the same width anteriorly (1).

7. Premaxilla adjacent to and anterior to narial fossa: elevated above the maxilla and forming a distinct lateral face (0); continuous or nearly continuous with the maxilla (1).

8. Premaxilla adjacent to and at posterior edge of nasal opening: does not clearly overhang maxilla (0); premaxilla overhangs maxilla (1).

9. Anterior portions of premaxillae: firmly contact each other (0); premaxillae are separated or only loosely contact along their entire length (1).

10. Suture between maxilla and premaxilla on rostrum: firmly articulated (0); loose (1).

11. Antorbital process: absent (0); present and defined by a steep face clearly separating the posterolateral corner of the maxilla from its more anterior rostral portion (1); present as a distinct anterior projection lateral to antorbital notch (2).

12. Anterior border of supraorbital process lateral to ascending process of the maxilla: bordered by lacrimal and maxilla (0); bordered by lacrimal only (1); as state 0, but with the antorbital process of the maxilla and the anterior border of the supraorbital process

separated by a basin (2); as state 0 but with maxilla overriding the anteriormost border of the supraorbital process (3).

13. Distinct pocket between the ascending process of the maxilla dorsally and the supraorbital process ventrally: absent (0); present (1).

14. Lateral process of maxilla: absent (0); present and clearly distinct from ascending process of maxilla (1); present and confluent with ascending process of maxilla (2).

15. Posterior portion of palatal surface of maxilla: flattened or slightly concave (0); medial portion of maxilla forms a longitudinal keel bordered laterally by a shallow longitudinal trough (1); medial portion forms a keel without any adjacent trough (2).

16. Palatal surface of anterior part of rostrum: flat or gently concave (0); bears pronounced longitudinal keel formed by the vomer and the medial edges of the maxillae (1).

17. Exposure of premaxilla on palate: exposed along at least one third of the medial border of the maxilla (0); limited in extent to less than one third of the medial border of the maxilla (1).

18. Palatal window exposing vomer: absent (0); present (1); narrow and variable exposure of vomer along most or all of the midline of the rostrum (2); vomer broadly exposed along the midline of the rostrum (3).

19. Palatal nutrient foramina and sulci: absent (0); present (1).

20. Outline of suture between maxillae and palatines: roughly straight transversely or bowed anteriorly (0); forms a posteriorly pointing V shape (1); anterior margins of palatines form two separate and posteriorly pointing U shapes (2).

21. Anteriormost point of palatine: located in line with or posterior to the level of the antorbital notch or equivalent point on rostrum (0); located anterior to the level of the antorbital notch (1).

22. Anterior portion of palatine distinctly concave transversely and forming a sharp median crest: absent (0); present (1).

23. Anterior edge of narial fossa: located in posterior three quarters of rostrum (0); located in anterior quarter of rostrum (1).

24. Facial portion of rostrum in lateral view: step-like (0); straight (1); concave (2). ORDERED

25. Rostrum shape: width at antorbital notches or equivalent point on rostrum less than 80% the length of the rostrum, as measured from its tip to the antorbital notches (0); width at antorbital notches or equivalent point more than 80% the length of the rostrum (1).

26. Teeth in adult individuals: present (0); absent or vestigial (1).

27. Upper dentition: comprises 10 teeth including M2 (0); includes 11 teeth including M3 or is polydont (1).

28. Large diastemata between posterior upper cheek teeth: absent (0); present (1).

29. Enamel ornamentation on premolars: vertical striations present on lingual surface only (0); heavy vertical striations present on both lingual and labial surfaces (1); vertical striations are poorly developed or absent (2); enamel is absent (3).

30. Shape of upper molars in lingual or labial view: crown base distinctly wider than crown height (0); crown base is shorter than or roughly equal to crown height (1).

31. Ectocingulum on P3 and P4: present (0); absent (1).

32. Entocingulum on P3 and P4: present (0); absent (1).

33. Proximal portions of posterior upper premolar and molar roots: separate (0); fused (1).

34. Posteriormost upper tooth: situated below anterior portion of orbit (0); situated anterior to anterior border of orbit (1).

35. Heavy planar wear resulting in loss of most or all of tooth crown: absent (0); present (1).

36. Outline of upper premolar crowns in anterior view: straight (0); curved with the apex pointing ventrally (1).

37. Heterodonty: present, with accessory denticles being well developed (0); absent or greatly reduced, with accessory denticles absent or tiny compared to main cusp (1).

38. Skull length about one third of total body length: absent (0); present (1).

39. Cranial asymmetry: present (0); absent (1).

40. Diameter of orbit as measured between the distalmost points of the preorbital and postorbital processes: less than 25% of bizygomatic width (0); 25% or more (1).

41. Anterior edge of supraorbital process lateral to ascending process of maxilla: oriented transversely or pointing anteriorly (0); pointing posteriorly (1); linguiform and tapering to a point (2).

42. Outline of anterior edge of supraorbital process in dorsal view: roughly straight or concave (0); distinctly sinusoidal (1).

43. Transverse width of anterior edge of supraorbital process lateral to ascending process of maxilla: longer than or equal to the combined transverse width of the adjacent rostral bones, as measured from the sagittal plane to the lateral border of the ascending process of the maxilla (0); shorter than the combined transverse width of the adjacent rostral bones (1).

44. Posterior border of supraorbital process in dorsal view: concave (0); straight (1).

45. Supraorbital process of frontal in anterior view: horizontal or nearly horizontal (0); gradually slopes away lateroventrally from the skull vertex (1); as state 1 but with the lateral portion of the supraorbital being nearly horizontal, thus causing the latter to appear concave in anterior view (2); abruptly depressed to a level noticeably below the vertex, with the lateral skull wall above the supraorbital formed by both parietal and frontal (3). ORDERED

46. Anterior and posterior borders of supraorbital process in dorsal view: roughly parallel or converging medially (0); converging laterally (1).

47. Width of supraorbital process as measured in a straight line from the lateralmost point of the postorbital process to the intertemporal constriction: equal to or shorter than the anteroposterior length of the supraorbital process above the orbit (0); up to twice the length above the orbit (1); more than twice the length above the orbit (2). ORDERED

48. Postorbital process in dorsal view: oriented posteriorly (0); oriented laterally (1); oriented posterolaterally (2); short and not markedly projecting in any direction (3).

49. Postorbital process in lateral view: pointed or rounded (0); forms and anteroposteriorly elongate triangle with a flattened posterior face (1).

50. Orbital rim of supraorbital process of frontal in lateral view: dorsoventrally thin (0); thickened with a flat lateral surface (1); thickened with a rounded lateral surface (2).

51. Position of anteriormost point of supraorbital process in dorsal view: in line with the posterior extremity of the nasals or passing through the nasals (0); at the same level as the anterior extremity of the nasals (1); anterior to the anterior extremity of the nasals (2). ORDERED

52. Dorsalmost point of orbit relative to lateral edge of rostrum (in lateral view, with skull resting on a horizontal surface): elevated above or roughly in line with the lateral edge of the rostrum (0); located well below the lateral edge of the rostrum (1).

53. Lacrimal in dorsal view: situated entirely lateral to the ascending process of the maxilla (0); lacrimal extends medially and separates the lateral corner of the ascending process from the more anterior portion of the maxilla (1).

54. Contact of jugal with zygomatic process of squamosal: the two bones overlap dorsoventrally (0); little or no overlap (1).

55. Anteriormost portion of jugal broadly underlapped by maxilla: absent (0); present (1).

56. Optic canal in ventral view: ventrally open (0); medial portion is enclosed by anterior and/or posterior bony laminae (1).

57. Postorbital ridge along medial portion of optic canal: absent or anteroposteriorly thin, with the optic canal running adjacent to the posterior border of the supraorbital process (0); well developed and thickened, thus resulting in the displacement of the optic canal away from the posterior border of the supraorbital process (1).

58. Maxillary infraorbital plate: absent (0); present (1).

59. Maxillary window originating from posterior border of infraorbital plate: absent (0); present (1).

60. Anteromedial corner of supraorbital process extending to a point medial to antorbital notch: absent (0); present (1).

61. Preorbital region of frontal in lateral view: thickened compared to more central portions of the orbit (0); dorsoventrally flat (1).

62. Enlarged dorsal infraorbital foramen on ascending process of maxilla, opening into a posterodorsally directed sulcus: absent (0); present (1).

63. Premaxillary sac fossa: absent (0); present (1).

64. Premaxillary foramen: absent (0); present (1).

65. Suture between maxilla and frontal: contact between the bones is straight or maxilla overrides anteromedial corner of the frontal (0); maxilla overrides half or more of the frontal (1).

66. Lateral borders of ascending process of maxilla: lateral edges convergent with the process tapering to a point (0); lateral edges parallel or divergent posteriorly (1).

67. Posterior end of ascending process of premaxilla: no contact with frontal or contacts the frontal with the posteriormost tip only (0); forms a robust contact with the frontal (1).

68. Triangular wedge of frontal separating ascending process of maxilla from nasal or premaxilla: absent (0); present (1).

69. Posterior ends of ascending processes of maxillae in dorsal view: separated by either frontals or both nasals and premaxillae (0); converging towards the midline and separated by nasals only (1); contact each other medially (2). ORDERED

70. Relative position of posteriormost edge of ascending process of maxilla in dorsal view: approximately in transverse line with, or posterior to, posterior edge of nasal (0); anterior to posterior edge of nasal (1).

71. Shape of posterior border of ascending process of maxilla: pointed or rounded (0); squared off (1).

72. Lateral profile of cranium along exposure of parietals on vertex: dorsal edge of parietal ascends steeply towards posterior edge of skull at an angle of 10 degrees or more, measured relative to the lateral edge of rostrum (0); dorsal edge of parietal is low to flat with an angle of less than 10 degrees (1).

73. Length of nasal relative to bizygomatic width: less than 50% of bizygomatic width (0); more than 50% of bizygomatic width (1).

74. Lateral margins of nasal: parallel (0); posteriorly convergent (1).

75. Anterior margins of nasals: roughly straight or U-shaped (0); form a distinct, posteriorly pointing W-shape (1); with point on midline and a gap on each side between premaxilla and nasal (2); form an anteriorly pointing W shape (3).

76. Dorsal surface of nasals: flattened (0); developed into a sagittal keel (1).

77. Separation of posterior portions of nasals along sagittal plane by narial process of frontal: present (0); absent (1).

78. Zygomatic process of squamosal and exoccipital in dorsal view: clearly separated by an angle (0); posterior border of zygomatic process and lateral edge of exoccipital are confluent, forming a continuous or nearly continuous lateral skull border (1).

79. Orbitotemporal crest: positioned along posterior border of supraorbital process, with the origin of the temporal muscle facing posteriorly or posteroventrally (0); absent or positioned on the dorsal surface of the supraorbital process, with the origin of the temporal muscle facing posterodorsally or dorsally (1).

80. Area enclosed by orbitotemporal crest on supraorbital process of frontal: forms less than half of the dorsal surface of the supraorbital process (0); covers half or more of the dorsal surface of the supraorbital process (1).

81. Outline and orientation of orbitotemporal crest: subparallel to posterior border of supraorbital process (0); distal half oriented distinctly posterolaterally and approaching the posterolateral corner of the supraorbital process (1); as state 1, but with the crest terminating halfway along the posterior border of the supraorbital process (2); as state 2, but with the crest being distinctly U-shaped (3).

82. Shape of temporal fossa: longer anteroposteriorly than wide transversely, or as wide as long (0); wider than long (1).

83. Intertemporal constriction: longer anteroposteriorly than wide transversely (0); as state 1, but with the temporal fossa forming a large parasagittal oval (1); wider transversely than long anteroposteriorly (2).

84. Exposure of frontal on skull vertex: broadly exposed (0); anteroposteriorly compressed or absent (1).

85. Parietal and interparietal: anteriormost point located no further forward than postorbital process (0); anteriormost point in line with supraorbital process (1).

86. Outline of fronto-parietal suture: straight or lobate (0); frontals projects posteriorly along the sagittal plane and separate the left and right parietal anteriorly (1); highly irregular (2).

87. Parietal in lateral view: as long or longer anteroposteriorly than high dorsoventrally (0); higher dorsoventrally than long anteroposteriorly (1).

88. Spreading of anterolateral portion of parietal on to posteromedial corner of supraorbital process of frontal: absent (0); present (1).

89. Anteriormost point of parietal and interparietal: more posterior than the posterior border of the ascending process of the maxilla (0); more anterior than or in line with the posterior border of the ascending process of the maxilla (1).

90. Anteriormost point of supraoccipital in dorsal view: located posterior to or in line with the anterior border of the squamosal fossa (0); in line with temporal fossa, but posterior to the apex of the zygomatic process of the squamosal (1); in line with or located anterior to the level of the apex of the zygomatic process of the squamosal (2); in line with the anterior half or anterior edge of the supraorbital process (3). ORDERED

91. Anteroposterior position of posterior apex of nuchal crest: posterior to the occipital condyle (0); anterior to or in line with the posteriormost point of the occipital condyle (1).

92. Mediolateral position of posterior apex of nuchal crest: aligned with the medial half or halfway point of the temporal fossa (0); approaching the level of the lateral border of the temporal fossa (1).

93. Distinct nuchal tubercle at junction of parieto-squamosal suture and supraoccipital: absent (0); present (1).

94. Exposure of alisphenoid within or at ventral border of temporal fossa: exposed on temporal wall of skull and contributing to orbital fissure (0); alisphenoid separated from orbital fissure or not exposed on temporal skull wall (1).

95. Postparietal foramen located at junction of parietal and squamosal: absent (0); present (1).

96. Zygomatic process of squamosal extremely well developed and robust in dorsal view: absent (0); present (1).

97. Zygomatic process of squamosal dorsoventrally expanded in lateral view: absent (0); present, with the zygomatic process being distinctly higher dorsoventrally than wide transversely (1).

98. Orientation of zygomatic process of squamosal in dorsal view: directed anteromedially (0); directed anteriorly or slightly anterolaterally (1); directed anterolaterally (2). ORDERED

99. Zygomatic process of squamosal in lateral or ventral view: tapering anteriorly (0); expanded anteriorly, thus forming a central constriction (1).

100. Twisting of zygomatic process of squamosal: absent (0); present, with the zygomatic process being partially twisted clockwise on the left and anticlockwise on the right (1); as state 1, but with the process twisted almost 90 degrees (2).

101. Position of apex of zygomatic process of squamosal: situated entirely posterior to the postorbital process (0); closely apposed to the postorbital process, or situated ventral to the latter (1).

102. Apex of zygomatic process of squamosal deflected anteroventrally: absent (0); present (1).

103. Supramastoid crest of zygomatic process of squamosal: present (0); absent (1).

104. Size of squamosal including zygomatic and postglenoid processes: longer anteroposteriorly than high dorsoventrally, or about as high as long (0); distinctly higher than long (1).

105. Parieto-squamosal suture shaped like a crest or ridge: absent or low (0); present and distinctly elevated (1).

106. Squamosal prominence: present as a projection on the crest delimiting the lateral or posterolateral edge of the squamosal fossa (0); absent (1).

107. Transverse width of squamosal lateral to exoccipital: width equal to or greater than 15% of the distance between the sagittal plane and the lateral edge of the exoccipital (0); exposed portion of squamosal is less than 15% of that distance (1).

108. Length of squamosal fossa relative to maximum transverse width of temporal fossa, as measured in a straight line from the posteriormost point of the temporal fossa to the posteriormost point of the nuchal crest: length of squamosal fossa is three quarters the width of the temporal fossa or longer (0); length of squamosal fossa is less than three quarters the width of the temporal fossa (1).

109. Squamosal cleft: absent (0); present (1).

110. Squamosal crease: absent (0); present (1).

111. Paired tubercles on supraoccipital: absent (0); limited to low ridges forming the lateral edges of a medial fossa (1); present (2). ORDERED

112. Lateral edge of supraoccipital in dorsal view: convex (0); straight (1); concave (2); sigmoidal (3).

113. Anterior border of supraoccipital shield: rounded or pointed (0); squared (1).

114. Overall outline of supraoccipital in dorsal view: rounded (0); triangular (1).

115. Anterior half of dorsal surface of supraoccipital: concave (0); flat or convex (1).

116. External occipital crest: absent or faint (0); restricted to anterior half of supraoccipital shield (1); present and running all the way along the supraoccipital shield (2). ORDERED

117. Tip of postglenoid process in lateral view: curving anteriorly (0); pointing ventrally (1); pointing posteriorly (2).

118. Ventral edge of postglenoid process in lateral view: approximately in line with or dorsal to the ventral edge of the exoccipital (0); extending well ventral to the ventral edge of the exoccipital (1).

119. Orientation of postglenoid process in posterior view: ventrolateral (0); ventral (1); ventromedial (2). ORDERED

120. Outline of postglenoid process in anterior or posterior view: parabolic (0); as state 0, but with lateral and medial edges parallel or concave (1); as state 1, but distinctly wider transversely than high dorsoventrally (2); triangular (3); trapezoidal, with a ventrally directed medial border (4).

121. Twisting of postglenoid process in ventral view: absent (0); twisted clockwise on the left side and anticlockwise on the right side so that the glenoid cavity faces anteromedially (1).

122. Position of base of postglenoid process in ventral or posterior view: in line with the lateral edge of the skull (0); shifted away medially from the lateral edge of the skull (1).

123. Medial border of postglenoid process in ventral view: confluent with more medial portion of squamosal (0); offset from remainder of squamosal by a distinct ridge (1).

124. Choanal margin of palatine in ventral view: absent (0); straight or convex (1); concave (2); forms a longitudinal notch (3).

125. Pterygoid in ventral view: partially or entirely exposed (0); palatine almost completely covers pterygoid and extends on to the hamular process (1).

126. Anteriormost point of pterygoid sinus fossa: located anterior to foramen pseudovale (0); approximately in line with anterior edge of foramen pseudovale (1); located posterior to anterior edge of foramen pseudovale (2). ORDERED

127. Superior lamina of pterygoid: absent or restricted to anteromedial quarter of pterygoid sinus fossa (0); present and covering half or more of ventral exposure of alisphenoid within pterygoid sinus fossa (1).

128. Shape of pterygoid hamulus: finger-like (0); expanded into a dorsoventrally flattened plate flooring the pterygoid sinus fossa (1); triangular and wing-like (2); reduced in size or almost absent (3).

129. Position of pterygoid hamuli in ventral view: located directly adjacent to the sagittal plane and almost contacting each other (0); well separated from each other (1).

130. Position of foramen pseudovale: foramen located within squamosal or between squamosal and pterygoid, and opening anterolaterally or laterally (0); as state 0, but with foramen opening posteriorly (1); foramen lies within pterygoid (2).

131. Foramen pseudovale raised above more lateral portions of squamosal in ventral view: absent (0); present (1).

132. Fossa on squamosal for reception of sigmoid process of tympanic bulla: present (0); absent or poorly defined (1).

133. Base of postglenoid process in ventral view: in transverse line with or located posterior to the posterior half of the tympanic bulla (0); in transverse line with or located anterior to the anterior half of the tympanic bulla (1); in transverse line with the anteroposterior centre of the tympanic bulla (2). ORDERED

134. Ventral border of sagittal part of vomer (nasals septum) in ventral view: posteriormost portion projects beyond the posterior border of the palatines and is visible in ventral view (0); completely covered by palatines (1).

135. Basioccipital crest: narrow transversely (0); wide and bulbous (1).

136. Lateral border of basioccipital crest in ventral view: straight (0); concave (1).

137. Orientation of basioccipital crests in ventral view: diverging posteriorly (0); parallel or subparallel (1).

138. Ventromedial corner of paroccipital process in posterior view: located more ventrally than the basioccipital crest (0); level with or more dorsal than the basioccipital crest (1).

139. Posteriormost point of exoccipital in dorsal view: located more anteriorly than posterior edge of occipital condyle (0); level with or posterior to posterior edge of condyle (1).

Hyoid

140. Outline of stylohyal in cross section: cylindrical (0); flattened (1).

141. Orientation of thyrohyal in dorsal or ventral view: oriented posteriorly (0); oriented laterally (1).

142. Shape of thyrohyal: cylindrical (0); flattened and wing-like (1); plate-like (2).

143. Ankylosed basihyal and thyrohyals: absent (0); present (1).

Periotic

144. Dorsal and medial elongation of pars cochlearis towards cranial cavity: absent (0); present (1); as state 1, but with only the anterior side of the pars cochlearis being elongated (2).

145. Attachment of anterior process to pars cochlearis: absent (0); present (1).

146. Anterior process of periotic in lateral view: squared off or rounded (0); triangular (1); anterior border of process is two-bladed and L-shaped (2).

147. Shape of anteroventral angle of anterior process of periotic in medial or lateral view: rounded or forms a relatively blunt angle (0); slender and tapering to a point (1).

148. Ventral edge of anterior process of periotic in medial view: at the same level or dorsal to ventral edge of pars cochlearis (0); ventral to ventral profile of pars cochlearis (1).

149. Dorsal deflection of anterodorsal corner of anterior process: absent (0); present (1).

150. Anterior process transversely compressed and blade-like: absent (0); present (1).

151. Length of anterior process of periotic: shorter than the anteroposterior length of the pars cochlearis, as measured from the anterior border of the pars cochlearis to the medial border of the fenestra rotunda (0); same length or longer than the pars cochlearis (1).

152. Anteroexternal sulcus: forms an oblique or vertical groove on lateral side of anterior process, immediately anterior to lateral tuberosity (0); absent (1).

153. Pyramidal process: absent (0); present (1).

154. Articulation of anterior process of periotic and tympanic bulla: no contact, or contact with accessory ossicle via fovea epitubaria on the anterior process of the periotic (0); accessory ossicle or homologous region on periotic fused to bulla (1).

155. Anterior bullar facet: present (0); absent (1).

156. Lateral tuberosity of anterior process: absent or relatively small and rounded (0); well-developed and distinctly triangular (1); hypertrophied and blade-like (2); forms a distinct shelf (3).

157. Position of lateral tuberosity: situated posterolateral to anterior pedicle of tympanic bulla or fovea epitubaria (0); situated lateral or anterolateral to anterior pedicle of tympanic bulla or fovea epitubaria (1).

158. Body of periotic lateral to pars cochlearis hypertrophied: absent (0); present laterally and ventrally (1); present laterally only (2).

159. Mallear fossa: well excavated and possessing a clearly defined rim (0); present only as a depression with diffuse edges (1).

160. Distinct ridge delimiting insertion surface of tensor tympani on medial side of anterior process: absent (0); absent, but insertion surface distinctly excavated (1); present (2).

161. Dorsal extension of attachment area for tensor tympani on medial side of anterior process: absent or indistinct (0); present as a deeply excavated canal (1).

162. Anteromedial corner of pars cochlearis in ventral view: developed as a rounded, anteroposterior ridge (0); angular and projecting medially, resulting in a flattened ventral surface of the pars cochlearis (1); smooth and rounded (2).

163. Promontorial groove on medial side of pars cochlearis: present, but relatively shallow (0); present and deeply excavated (1); present and forming a distinct constriction, separating a smooth and rounded ventral portion of the pars cochlearis from a flattened and striated dorsal one (2); absent (3).

164. Caudal tympanic process in posteromedial view: well separated from crista parotica (0); narrow separation or contact (1).

165. Posteromedial corner of pars cochlearis medial to fenestra rotunda: rounded and level with fenestra rotunda (0); inflated and projecting posteriorly beyond fenestra rotunda (1).

166. Morphology of caudal tympanic process: developed as a posteriorly extending triangular shelf (0); as state 0, but with the ventral border bulging ventrally (1); as state 0 but pointing posterodorsally (2); developed as a robust, ventrally directed projection (3); absent or poorly developed (4).

167. Elongate lobe and fossa extending posteromedially from fenestra ovalis in ventral view: absent (0); present (1).

168. Anteroposterior alignment of proximal opening of facial canal, internal acoustic meatus and aperture for cochlear aqueduct: present (0); absent (1).

169. Anteroposterior alignment of aperture for cochlear aqueduct and aperture for vestibular aqueduct: absent (0); present (1).

170. Prominent septum dividing foramina for vestibular and cochlear nerves within internal acoustic meatus: present (0); absent (1).

171. Shape of aperture for cochlear aqueduct: round with sharply defined dorsal margins (0); slit-like (1).

172. Size of aperture for cochlear aqueduct: smaller than aperture for vestibular aqueduct (0); approximately the same size (1).

173. Aperture for cochlear aqueduct and fenestra rotunda: separate (0); confluent (1).

174. Superior process: present as a distinct crest forming the lateral wall of the suprameatal fossa (0); the lateral border of the suprameatal fossa is low and not clearly defined (1).

175. Suprameatal area hypertrophied: absent (0); present (1).

176. Development of crista transversa: depressed well below the rim of the internal acoustic meatus (0); well developed and reaching the cerebral surface of the pars cochlearis (1).

177. Morphology of crista transversa: developed as a septum of varying thickness (0); proximal opening of facial canal is widely separated from internal acoustic meatus and connected to the latter via a distinct sulcus (1).

178. Hiatus Fallopii: absent or small opening located anterior or anteroventral to proximal opening of facial canal (0); as state 0, but with the hiatus Fallopii being well developed and large (1); anterior border of proximal opening of facial canal is continuous with the hiatus Fallopii and shaped like a fissure (2).

179. Size of proximal opening of facial canal: no more than half the size of the internal acoustic meatus (0); more than half the size of the internal acoustic meatus (1).

180. Squamosal flange located posterior to lateral tuberosity: absent (0); present (1).

181. Articulation surfaces on posterior processes of tympanic bulla and periotic: unfused (0); fused in adults to form compound posterior process (1).

182. Morphology of facial sulcus on distal half of compound posterior process: absent or relatively shallow sulcus with equally defined anterior and posterior borders (0); marked groove with an elevated anterior border (1); deeply incised canal (2); as state 2, but with the facial sulcus being partially or entirely floored, tubular and present along the entire ventral surface of the compound posterior process (3).

183. Position of facial sulcus on compound posterior process in ventral view: facial sulcus runs close to or along the posterior border of the compound posterior process (0); facial sulcus located centrally on the ventral surface of the compound posterior process (1).

184. Orientation of compound posterior process in ventral view, with periotic being in situ: oriented posterolaterally with respect to the longitudinal axis of the anterior process of the periotic (0); oriented at a right angle to the axis of the anterior process (1).

185. Shape of compound posterior process: cylindrical or slightly conical (0); short and stocky (1); flattened anteroposteriorly (2); forms a distinct plug (3).

186. Exposure of compound posterior process on lateral skull wall: external surface of compound posterior process is absent or poorly defined (0); external surface is present but distinct from lateral skull wall (1); lateral surface is expanded and firmly integrated into the

lateral skull wall (2); as state 2, but with the exposed lateral surface of the compound posterior process being concave and defined by a distinct ridge separating it from the ventral surface (3). ORDERED

187. Bony texture of ventral surface of compound posterior process: massive (0); fibrous (1).

188. Neck of compound posterior process markedly constricted: absent (0); present (1).

Tympanic bulla

189. Anterior border of bulla in dorsal or ventral view: obliquely truncated (0); squared (1); rounded (2); pointed (3).

190. Anterior portion of bulla transversely wider than posterior portion in ventral view: absent (0); present (1).

191. In situ orientation of main axes of tympanic bullae in ventral view: diverging posteriorly (0); parallel (1); diverging anteriorly (2). ORDERED

192. Position of dorsal origin of lateral furrow: located along posterior two thirds of the anteroposterior length of the bulla (0); located at roughly one third of the anteroposterior length of the bulla (1).

193. Orientation of lateral furrow in lateral view: ventral (0); distinctly anteroventral (1).

194. Orientation of ventral keel of lateral lobe of bulla: faces ventrally (0); faces ventromedially or medially (1).

195. Anteroposterior outline of lateral lobe or main ridge of bulla: concave (0); straight or convex (1).

196. Position of involucral ridge in dorsal view: coincident with medial edge of the bulla (0); laterally retracted (1).

197. Sigmoid process deflected laterally in anterior or posterior view: absent (0); present (1); as state 1, but with the sigmoid process being nearly horizontal (2). ORDERED

198. Dorsomedial corner of sigmoid process in anterior view: separated from the pedicle of the malleus (0); confluent with the pedicle of the malleus (1).

199. Ventral margin of sigmoid process in lateral view: present (0); absent, with the lateral margin of the sigmoid process turning smoothly into a sulcus on the lateral side of the bulla (1).

200. Shape of conical process in lateral view: well developed and dorsally convex (0); reduced to a low ridge or absent (1).

201. Elliptical foramen: present (0); absent (1).

202. Medial lobe of tympanic bulla: present as distinct lobe and transversely wider than its lateral counterpart (0); present but subequal in width to the lateral lobe or smaller (1); absent or indistinct (2). ORDERED

203. Crest connecting medial and lateral lobes of tympanic bulla in posterior view: present (0); absent (1).

204. Anteriormost point of involucral ridge: extends anteriorly to form the anteriormost point of the bulla (0); in line with or posterior to the anterior border of the bulla (1).

205. Dorsolateral surface of involucrum: divided into a low anterior and an elevated posterior portion, separated by a clearly defined step (0); forms a continuous rim (1).

206. Transverse creases on dorsal surface of involucrum: poorly developed or absent (0); well defined and deep (1).

207. Ridge on inside of bulla: extends laterally from involucrum and partially divides cavum tympani into anterior and posterior portions (0); absent (1).

208. Development of tympanic sulcus: developed as a faint line (0); forms a distinct crest or sulcus (1).

209. Outline and position of tympanic sulcus: forms a semicircular and ventrally curved line well separated from the intersection of the conical and sigmoid processes (0); forms a roughly horizontal line at or close to the level of the intersection of the conical and sigmoid processes (1).

210. Anteromedial portion of ventral surface of tympanic bulla: transversely convex (0); distinctly flattened or slightly concave (1).

211. Anterolateral corner of bulla: broadly rounded (0); inflated and forming a distinct lobe (1); flattened (2).

212. Anterolateral ridge or shelf: absent (0); present (1).

213. Position of posterior pedicle of tympanic bulla in dorsal view: situated at or near the posterior border of the bulla (0); located far anterior to the posterior end of the bulla (1).

Mandible

214. Posterior mandibular cheek teeth: oriented vertically or anteriorly (0); reclined posteriorly (1).

215. Medial surface of central part of mandible: similar to lateral surface (0); distinctly flattened relative to lateral surface (1).

216. Dorsomedial surface of posterior portion of mandibular body: flat or convex (0); distinctly excavated (1).

217. Mandibular symphysis: sutured or fused (0); unfused (1).

218. Outline of posterior portion of mandible in dorsal or ventral view: follows a straight line or simple curve (0); sigmoidal owing to a laterally reflexed neck and condyle (1).

219. Mandibular body in dorsal view: bowed medially (0); straight (1); bowed laterally, but with curvature mainly confined to anterior portion of mandible (2); evenly bowed laterally (3). ORDERED

220. Anterior extremity of mandible: vertical or slightly twisted, with the ventral edge shifted medially (0); apex of mandible shifted to an almost horizontal position (1).

221. Mandibular body in medial or lateral view: height of ramus remains roughly constant throughout (0); arched dorsally (1); increases in height anteroposteriorly (2); dorsoventrally constricted near the centre (3); decreasing in height anteroposteriorly, with the anteriormost portion being distinctly expanded (4).

222. Height of mandibular foramen: dorsoventral height approximately that of the horizontal ramus, thus forming a mandibular fossa (0); dorsoventral height about half that of the horizontal ramus or less (1).

223. Anterior border of mandibular foramen: rounded (0); sharply triangular (1).

224. Dorsal border of mandibular foramen projected medially and developed into a roof: absent (0); present (1).

225. Satellite process: absent or limited to a low rugosity (0); present (1).

226. Ridge on posteromedial portion of coronoid process running close and parallel to its posterior border: absent (0); present (1).

227. Relative position of anterior border of mandibular foramen: in line with coronoid process (0); posterior to coronoid process (1).

228. Subcondylar furrow: absent or extremely shallow (0); present as a well-defined groove medially only (1); as state 1, but with the dorsal border of the furrow being accentuated by a medially well-developed condyle (2); extends across the posterior surface of the condyle, separating it from the angular process both medially and laterally (3). ORDERED

229. Coronoid process in lateral or medial view: forms a broad plate (0); distinctly triangular (1); shaped like a finger and pointing posteriorly (2).

230. Shape of coronoid process (if triangular) in lateral or medial view: sharply triangular and about as high dorsoventrally as long anteroposteriorly (0); bluntly triangular and considerably longer than high (1).

231. Anterior outline of coronoid process: vertical (0); bent laterally (1).

232. Postcoronoid elevation: absent (0); present (1).

233. Development of angular process in medial view: hollowed out (0); robust (1).

234. Anteroposterior position of angular process: located below the condyle, or slightly anterior (0); projects posteriorly beyond the condyle (1).

235. Angular process deflected ventrally absent (0); present (1).

236. Fossa on medial side of angular process absent (0); present (1).

237. Orientation of articular surface of mandibular condyle: posterior (0); posterodorsal (1); dorsal, with the condyle being confluent with a dorsoventrally expanded angular process (2); dorsal, with the condyle being larger than and clearly offset from the angular process (3).

238. Sulcus for attachment of mylohyoid muscle on ventromedial surface of mandible: absent (0); present (1).

Vertebral column

239. Height of transverse process of atlas at base: more than half the height of the articular surface (0); equal to half the height of the articular surface or less (1).

240. Foramen transversarium in axis: absent (0); present (1).

241. Development of parapophysis and diapophysis on axis in anterior or posterior view: parapophysis considerably more robust than diapophysis (0); parapophysis and diapophysis are similar in size (1).

242. Cervical vertebrae: separate (0); partially fused starting from the axis (1); completely fused (2). ORDERED

243. Parapophysis on seventh cervical vertebra: present (0); absent (1).

244. Centra of cervical vertebrae in anterior or posterior view: rounded (0); squared (1).

245. Orientation of transverse processes of anterior lumbar vertebrae in anterior or posterior view: oriented distinctly ventrolaterally (0); oriented slightly ventrolaterally or subhorizontally (1); oriented laterally and horizontally (2). ORDERED

246. Outline of transverse processes of lumbar vertebrae in anterior or posterior view: flattened or slightly thickened (0); robust and triangular, with a dorsoventrally thickened base reaching up to the ventral border of the neural arch (1).

247. Apices of neural spines of posterior thoracic and anterior lumbar vertebrae anteroposteriorly expanded and squared off: absent (0); present (1).

248. Number of lumbar vertebrae: more than 12 (0); 10 to 12 (1); fewer than 10 (2). ORDERED

249. Metapophyses on posterior thoracic and anterior lumbar vertebrae in lateral view: oriented dorsally (0); oriented anterodorsally or anteriorly (1).

Ribs and sternum

250. Sternum: composed of several bones (0); composed of one bone (1).

251. Posterior ribs transversely expanded: absent (0); present (1).

252. Outline of central ribs in lateral view: evenly curved (0); sigmoidal (1).

Forelimb

253. Proportions of scapula: anteroposterior length of scapula approximately equals or is less than its maximum dorsoventral height (0); maximum anteroposterior length clearly exceeds its maximum dorsoventral height (1).

254. Coracoid process of scapula: present (0); absent (1).

255. Acromion process of scapula: present (0); absent (1).

256. Supraspinous fossa of scapula: present (0); absent or nearly absent, with acromion process located near anterior edge of scapula (1).

257. Deltoid crest of humerus: present as a distinct crest (0); absent or reduced to a variably developed rugosity (1).

258. Humerus: longer than or roughly the same length as radius and ulna (0); distinctly shorter than radius and ulna (1).

259. Orientation of humeral head in medial or lateral view: angled (0); vertical (1).

260. Distal portion of humerus in medial or lateral view: distal epiphysis narrower than shaft (0); distal epiphysis equal to or flared compared to shaft (1).

261. Articulation facet for radius on humerus in medial or lateral view: radial and ulnar facets are subequal in size (0); radial facet is distinctly larger than ulnar facet, excluding the olecranon (1).

262. Olecranon process: present as a distinct process (0); absent (1).

263. Manus: pentadactyl (0); tetradactyl (1).

Hind limb

264. Femur: present as a relatively well-developed bone (0); absent or reduced to a barely recognisable lump with an extremely rough surface texture (1).

265. Tibia: present (0); absent (1).

Soft tissue

266. Ventral throat grooves: absent (0); present and terminate well anterior to umbilicus (1); present and extend to umbilicus (2).

267. Ventral throat pouch: absent (0); present (1).

268. Tongue: muscular (0); reduced and predominantly connective tissue (1).

269. Temporomandibular joint: synovial (0); fibrocartilagenous mass originates in the glenoid fossa and envelopes the mandibular condyle (1).

270. Longitudinal ridges on rostrum: absent or indistinct (0); single median ridge (1); three longitudinal ridges (2). ORDERED

271. Dorsal fin: present as fin or dorsal hump (0); absent (1).

272. Chromosome number: 42 (0); 44 (1).
